# Association between obstructive sleep apnea and chronic kidney disease: A cross-sectional and Mendelian randomization study

**DOI:** 10.1097/MD.0000000000041437

**Published:** 2025-02-07

**Authors:** Shaokang Wang, Yupei Cheng, Zhe Zhang, Wei Liu, Mi Ou, Tianlong Yin, Yalu Meng, Haipeng Ban, Wenlong Gu, Xianggang Meng, Lili Zhang, Yuzheng Du

**Affiliations:** a First Teaching Hospital of Tianjin University of Traditional Chinese Medicine, Tianjin, China; b National Clinical Research Center for Chinese Medicine Acupuncture and Moxibustion, Tianjin, China; c College of Integrative Medicine, Dalian Medical University, Dalian, China.

**Keywords:** chronic kidney disease, mediators, Mendelian randomization, NHANES, obstructive sleep apnea

## Abstract

Previous observational studies have shown that obstructive sleep apnea (OSA) was associated with chronic kidney disease(CKD). Early diagnosis of OSA usually helps better prevent the occurrence of CKD. This cross-sectional investigation was conducted using data from the National Health and Nutrition Examination Survey, which was carried out between 2007 to 2008 and 2015 to 2016. Logistic regression model was employed to assess the impact of OSA on CKD. We did a mediation analysis to assess how much of the effect of OSA on CKD was mediated through mediators. Additionally, Mendelian randomization (MR) analysis assessed the causal link between OSA and various measures of renal impairment and possible mediators: obesity, hypertension and type 2 diabetes mellitus. In the cross-sectional study, the results of unadjusted model showed that participants with OSA had a higher risk of CKD compared to non-OSA (OR = 1.14, 95% confidence intervals [CI]: 1.01–1.28, *P* < .05). In mediation analysis, the proportion of hypertension and obesity mediating the effect of OSA on CKD was 41.83% and 30.74%, respectively. Univariate MR analysis results showed that: genetically predicted OSA was associated with decreased estimated glomerular filtration rate_cystatin c_ (eGFR_cystatin c_) level (OR = 0.997, 95% CI: 0.995–0.999, *P* < .05), increased blood urea nitrogen (BUN) levels (OR = 1.023, 95% CI: 1.008–1.038, *P* < .05), increased serum creatinine levels (OR = 1.010, 95% CI: 1.002–1.018, *P* < .05), increased serum cystatin C levels (OR = 1.015, 95% CI: 1.005–1.026, *P* < .05). Multivariable MR results showed that obesity mediated the causal effect of OSA on eGFR_cystatin c_, BUN levels and serum cystatin C levels. The cross-sectional study revealed a positive relationship between OSA and CKD, which was mediated by hypertension and obesity. The MR analysis suggest that OSA was associated with several measures of renal impairment, which was mediated by obesity. These findings may inform prevention and intervention strategies against CKD.

## 1. Introduction

Obstructive sleep apnea (OSA) was a disorder characterized by the complete or partial collapse of the upper airway, accompanied by repetitive drops in blood oxygen saturation and nocturnal awakenings.^[1]^ Epidemiology showed that among adults aged 65 and above, the prevalence of OSA in males was as high as 90%, in females was as high as 78% and in preschool aged children was high as 20.4%.^[2,3]^ Untreated OSA was associated with various adverse outcomes, including atrial fibrillation, stroke, and even mortality.^[4,5]^ A meta-analysis indicated that severe OSA increased the relative risk of major cardiac events by 2.04 and all-cause mortality by 1.54.^[6]^ Chronic kidney disease (CKD) was widely recognized as a global public health issue, with about an estimated 119.5 million cases in China, exerting a substantial impact on socioeconomic factors.^[7]^ CKD was typically assessed using various indicators such as estimated glomerular filtration rate (eGFR), microalbuminuria, urinary protein-to-creatinine ratio, blood urea nitrogen (BUN), serum creatinine and serum cystatin C.

Multiple observational studies have shown a higher prevalence of renal impairment among OSA patients. The severity of OSA was directly proportional to the prevalence of CKD.^[8,9]^ In CKD patients, even after adjusting for factors such as age, gender, body mass index (BMI), etc, the proportion of OSA was notably high in moderate to severe of CKD.^[10]^ Therefore, these 2 diseases may act as mutually reinforcing risk factors, demonstrating a synergistic effect.^[8,11]^ Recognizing risk factors promptly was essential for the prevention and early therapeutic intervention of both diseases, including hypertension, type 2 diabetes mellitus (T2DM), obesity and coronary heart disease.^[12–14]^ However, due to the substantial overlap in risk factors between the 2, establishing a causal relationship between OSA and renal impairment proved challenging.

The National Health and Nutrition Examination Survey (NHANES) is a comprehensive, nationwide, publicly available medical database. This database features high-quality data and long-term follow-up results, making it particularly suitable for discussing the relationship between OSA and renal impairment. Mendelian randomization (MR) has been proven a method that utilized genetic variants as instrumental variables (IVs) to investigate the potential causal relationship between exposure and outcome. In this study, we employed a 2-sample MR analysis to explore the potential causal relationship between OSA and renal impairment. Additionally, we conducted multivariable MR (MVMR) and mediation analysis to assess the mediating effects of obesity.

## 2. Materials and methods

### 2.1. Overall study design

**Figure 1.**
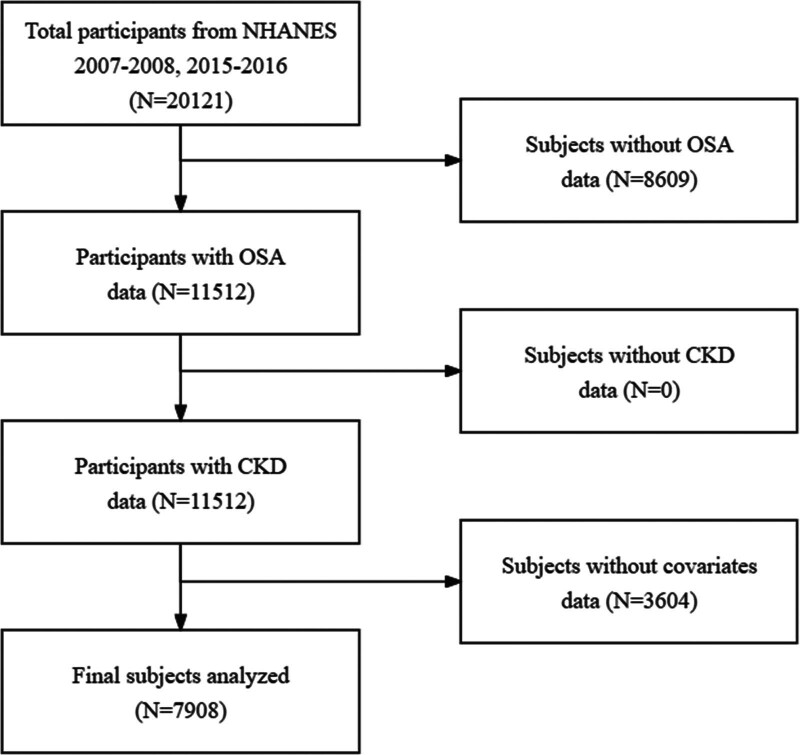
Participant selection process of cross-sectional study.

This study is divided into 2 main parts. The first part utilized data from the NHANES to investigate the association between OSA and renal impairment after adjusting for several potential confounding factors. The NHANES database samples representative noninstitutionalized populations in the United States every 2 years. The data used in this study can be accessed at https://www.cdc.gov/nchs/index.htm. We utilized data from 2 cycles spanning from 2007 to 2008 and 2015 to 2016. The framework of the cross-sectional study was shown in Figure 1. We used univariate and multivariate logistic regression to investigate the impact of OSA on CKD.

**Figure 2.**
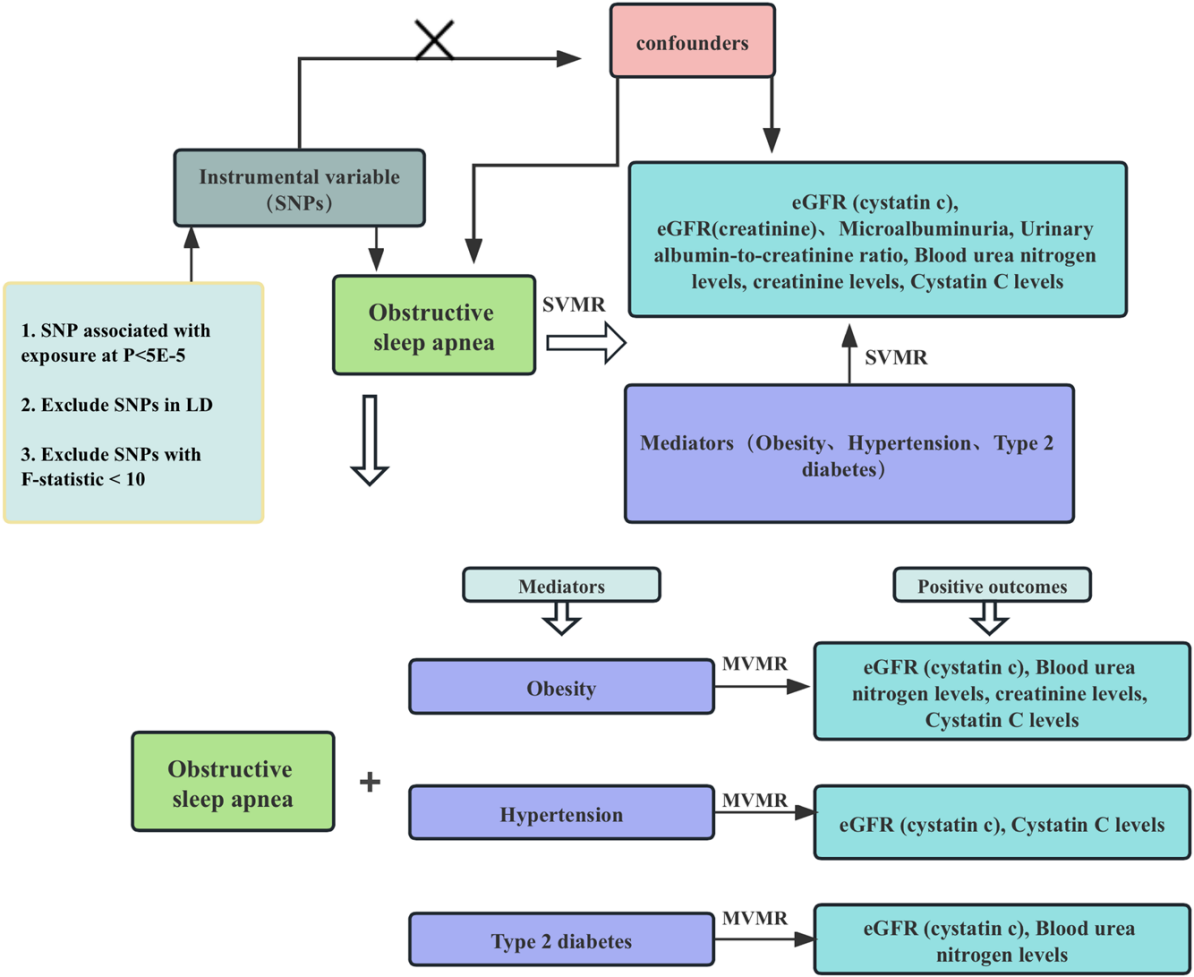
Flowchart of MR analysis conducted in this study. Notes: Univariate MR analysis investigates the effect of OSA on renal impairment and mediators, MVMR analysis evaluates the roles of mediators mediating the association between OSA and renal impairment. MR = Mendelian randomization, MVMR = multivariate Mendelian randomization, OSA = obstructive sleep apnea.

We employed a 2-sample MR analysis to explore the potential causal relationship between OSA and various measures of renal impairment, as well as MVMR and mediation analysis to assess the mediating effects. The framework of the current MR study was shown in Figure 2. This study was divided into 2 parts. In the first part, we explored the association between OSA and indicators of renal impairment (eGFR_cystatin c_, eGFR_creatinine_, microalbuminuria, urinary albumin-to-creatinine ratio, BUN levels, creatinine levels, cystatin C levels) by using univariate MR, and with candidate mediators (hypertension, T2DM, and obesity). We further explored the causal relationship between the candidate mediators and the positive outcomes. In the second part, we used MVMR to screen out the mediating factors between OSA and renal impairment, excluded the interference of confounders, and identified the mediating factors. In this MR study, we used genetic variants as IVs for MR analysis. Our MR effectiveness research hypotheses based on the following 3 core assumptions: correlation assumption: genetic variation is closely related to exposure; independence assumption: genetic variation is not associated with any confounders that may mediate the way from exposure to outcome; exclusion-restriction hypothesis: genetic variation may only affect the outcome through exposure. To exclude the effect of heterogeneity, Inverse variance weighting (IVW) was used as the main method, and MR-Egger and weighted median methods were used for validation.^[15]^

This study strictly adheres to the guidelines provided in the Strengthening the Reporting of Observational Studies in Epidemiology using MR (STROBE-MR checklist) and the Strengthening the Reporting of Observational Studies in Epidemiology (STROBE-cross-sectional studies checklist). This research did not require IRB approval because it does not involve any animal or human experiments. Additionally, the data used in this study all come from public databases. All studies that make up the GWAS and NHANES have existing ethical clearances from their respective institutional review boards, and participants provided informed consent.

### 2.2. Data sources

#### 2.2.1. Data for NHANES study

The present cross-sectional study utilized NHANES data from 2007 to 2008 and 2015 to 2016, obtained from the National Center for Health Statistics. Our study included individuals aged 20 years or older who completed the interview, excluding those missing data on OSA, CKD, or covariates.

The primary outcome, CKD, was considered a binary variable, defined as an estimated glomerular filtration rate (eGFR) below 60 mL/min/1.73m² and/or a urine albumin-to-creatinine ratio above 30 mg/g. The exposure variable, OSA, was also a binary variable, defined based on responses to 3 dichotomous questions. These included: how often do you snore? How often do you stop breathing while asleep? How often do you feel excessively sleepy during the day? Individuals who reported snoring 3 or more times per week, stopping breathing 3 or more times per week, and feeling excessively sleepy 16 to 30 times per month were classified as having OSA symptoms.

To reduce bias, potential covariates assessed in this study were based on existing literature, including age, gender, race, household income, education level, marital status, BMI, hypertension, high cholesterol, diabetes, and coronary heart disease. Racial categories included Non-Hispanic White, Non-Hispanic Black, Other Hispanic, Mexican American, and other races. Household income was categorized into 3 groups based on the poverty income ratio (PIR): low (≤1.3), middle (1.3–3.5), and high (>3.5). Education level was categorized as: <9th grade, 9 to 11th grade, high school graduate, some college or associate degree, and college graduate or above. Marital status was categorized as: married, widowed, divorced, separated, never married, or living with a partner. BMI (kg/m²) was calculated using standardized techniques based on weight and height measurements. Hypertension and high cholesterol were diagnosed based on responses of “yes” to the questions, “ever told you had high blood pressure” and “Doctor told you – high cholesterol level,” respectively. Diabetes was diagnosed based on the question, “have you ever been told by a doctor or health professional that you have diabetes or sugar diabetes?” with responses categorized as “yes,” “no,” or “borderline.” Coronary heart disease was diagnosed based on a “yes” response to the question, “ever told you had coronary heart disease.” The detailed information is shown in Table 1.

**Table 1:** The baseline characteristics of the study population.

Variable	Total (N = 7908)	No OSA (N = 5465)	OSA (N = 2443)	*P*-value*
Age (yr), N (%)				
<60	4989 (63.09%)	3436 (62.87%)	1553 (63.57%)	.553
≥60	2919 (36.91%)	2029 (37.13%)	890 (36.43%)	
Gender, N (%)				
Male	3760 (47.55%)	2461 (45.03%)	1299 (53.17%)	**<.001**
Female	4148 (52.45%)	3004 (54.97%)	1144 (46.83%)	
Race, N (%)				
Mexican American	1227 (15.52%)	842 (15.41%)	385 (15.76%)	.104
Other Hispanic	927 (11.72%)	639 (11.69%)	288 (11.79%)	
Non-Hispanic White	3329 (42.10%)	2283 (41.77%)	1046 (42.82%)	
Non-Hispanic Black	1604 (20.28%)	1099 (20.11%)	505 (20.67%)	
Other race	821 (10.38%)	602 (11.02%)	219 (8.96%)	
Education, N (%)				
<9th grade	881 (11.14%)	641 (11.73%)	240 (9.82%)	**<.001**
9th to 11th grade	1009 (12.76%)	671 (12.28%)	338 (13.84%)	
High school grade/GED or equivalent	1772 (22.41%)	1208 (22.10%)	564 (23.09%)	
Some College or AA degree	2293 (29.00%)	1532 (28.03%)	761 (31.15%)	
College graduate or above	1953 (24.70%)	1413 (25.86%)	540 (22.10%)	
Marital				
Married	4333 (54.79%)	2909 (53.23%)	1424 (58.29%)	**<.001**
Widowed	646 (8.17%)	487 (8.91%)	159 (6.51%)	
Divorced	894 (11.31%)	599 (10.96%)	295 (12.08%)	
Separated	260 (3.29%)	181 (3.31%)	79 (3.23%)	
Never married	1170 (14.80%)	879 (16.08%)	291 (11.91%)	
Living with partner	605 (7.65%)	410 (7.50%)	195 (7.98%)	
PIR, N (%)				
<1.3	2261 (28.59%)	1585 (29.00%)	676 (27.67%)	.442
≥1.3, <3.5	3119 (39.44%)	2136 (39.09%)	983 (40.24%)	
≥3.5	2528 (31.97%)	1744 (31.91%)	784 (32.09%)	
BMI (kg/m^2^), mean (SD)	29.56 ± 6.89	28.56 ± 6.39	31.80 ± 7.43	**<.001**
Hypertension, N (%)				
Yes	3116 (39.40%)	1965 (35.96%)	1151 (47.11%)	**<.001**
No	4792 (60.60%)	3500 (64.04%)	1292 (52.89%)	
High cholesterol, N (%)				
Yes	3123 (39.49%)	1981 (36.25%)	1142 (46.75%)	**<.001**
No	4785 (60.51%)	3484 (63.75%)	1301 (53.25%)	
Diabetes, N (%)				
Yes	1158 (14.64%)	695 (12.72%)	463 (18.95%)	**<.001**
No	6582 (83.23%)	4678 (85.60%)	1904 (77.94%)	
Borderline	168 (2.12%)	92 (1.68%)	76 (3.11%)	
Coronary heart disease, N (%)				
Yes	365 (4.62%)	215 (3.93%)	150 (6.14%)	**<.001**
No	7543 (95.38%)	5250 (96.07%)	2293 (93.86%)	
Chronic kidney disease, N (%)				
No	6331 (80.06%)	4411 (80.71%)	1920 (78.59%)	**.029**
Yes	1577 (19.94%)	1054 (19.29%)	523 (21.41%)	

The bold text signifies a statistically significant distinction with a *P* < .05.

AA = associate of arts degree, BMI = body mass index, GED = general educational development, OSA = obstructive sleep apnea, PIR = poverty income ratio, SD = standard deviation.

* For continuous variables, the Kruskal–Wallis rank sum test was used, while for count variables with theoretical numbers less than 10, Fisher exact probability test was used to obtain the results.

#### 2.2.2. Data for Mendelian randomization

All variables from the largest publicly available genetic tools GWAS summary statistics (https://gwas.mrcieu.ac.uk),^[16]^ to avoid the crowd heterogeneity bias, we mainly adopted the European population summary data. Data sets for OSA, hypertension, and T2DM were obtained from FinnGen biobank analysis round 5, data on eGFR_cystatin c_, BUN, creatinine, and cystatin C were obtained from EBI database, the data sets of obesity, microalbuminuria and urinary albumin-to-creatinine ratio were from the IEU database. The respective sample sizes were as Table 2. All studies that make up the GWAS have existing ethical clearances from their respective institutional review boards, and participants provided informed consent.

**Table 2:** Detailed information for the GWAS data.

Phenotype	Consortium or author	Sample size	GWAS ID
Exposure			
OSA	NA	16,380,465 SNPs	finn-b-G6_SLEEPAPNO
Outcomes			
EGFR (cystatin c)	Stanzick	460,826 individuals	ebi-a-GCST90103635
EGFR (creatinine)	Stanzick	1004,040 individuals	ebi-a-GCST90103634
Microalbuminuria	CKDGen	54,116 individuals	ieu-a-1097
Urinary albumin-to-creatinine ratio	CKDGen	54,450 individuals	ieu-a-1107
Blood urea nitrogen	Sakaue	148,767 individuals	ebi-a-GCST90018728
Creatinine	Sakaue	344,104 individuals	ebi-a-GCST90018979
Cystatin C	Neale lab	13,586,047 individuals	ukb-d-30720_irnt
Mediator			
Obesity	GIANT	9889 cases, 62,657 controls	ieu-a-91
Hypertension	NA	55,917 cases, 162,837 controls	finn-b-I9_HYPTENS
T2DM	NA	29,166 cases, 183,185 controls	finn-b-E4_DM2_STRICT

EGFR (creatinine) = estimated glomerular filtration rate creatinine, EGFR (cystatin c) = estimated glomerular filtration rate cystatin c, OSA = obstructive sleep apnea, T2DM = type 2 diabetes mellitus.

### 2.3. Selection of genetic instrumental variables

In order to screen eligible genetic IVs that met the MR Hypothesis, a series of tests were required. First, to obtain a sufficient number of IVs and increase statistical power, we set the *P*-value threshold of IVs at 5 × 10^−05^ in this MR study to screen for single-nucleotide polymorphisms (SNPS) that were strongly associated with exposure. Second, to set independence to eliminate linkage disequilibrium (LD; r2 < 0.001, window size = 10,000 kb), and calculate the statistical strength (*F*-statistic), *F* value >10 indicates that there was no weak IV bias. *F*-statistic values for each instrument-exposure association ranged from 19.57 to 27.89 (Supplementary document 1, Supplemental Digital Content, http://links.lww.com/MD/O349). In addition, coordination of exposure and results data sets was required to ensure that the same alleles have the same effect. SNPS screened through these rigorous procedures can be used as IVs for subsequent analysis.

### 2.4. Statistical analysis

In the NHANES study, we first described the baseline characteristics of the overall sample, the OSA group, and the non-OSA group. Continuous variables were expressed as means and standard deviations, and categorical variables as frequencies and percentages. Differences between the OSA and non-OSA groups were tested using t-tests for continuous variables and chi-square tests for categorical variables. Multivariable logistic regression analysis was used to examine the relationship between OSA and CKD. Odds ratios (OR) and 95% confidence intervals (CI) were calculated to assess the strength of the association. The unadjusted model was not adjusted for any factors; Model 1 was adjusted for gender, age, and race; Model 2 added household income, education level, and marital status as covariates to Model 1; and Model 3 further adjusted for comorbid conditions, including hypertension, high cholesterol, diabetes, and coronary heart disease. Parallel mediation analysis was used to estimate the potential mediating effect of hypertension and obesity in the association between OSA and CKD. We defined obesity as having a BMI of 30 or higher. The parallel mediation model used individual indicators as mediators. The direct effect (DE) was the effect of OSA on CKD without the mediator. The indirect effect (i.e.) was the effect of OSA on CKD through the mediator. The proportion of the mediating effect was estimated by dividing the i.e. by the total effect. All analyses were performed using R 3.6.1 software (R Foundation for Statistical Computing, Vienna, Austria; http://www.rproject.org/) and EmpowerStats (http://www.empowerstats.com). A *P*-value of <.05 was considered statistically significant.

Two sample MR MR-PRESSO, MVMR, LASSO packages in R software were used for analysis package, IVW was employed as the primary method to assess causal estimates,^[17]^ with MR-Egger and weighted median methods used for validation. The IVW approach employed IVs to study causality and address confounding factors. In this context, SNPs served as IVs, and their effect size (beta value) and standard error (SE value) were essential data for MR analysis. This method’s strength lies in its ability to derive accurate causal estimates if SNPs satisfy the 3 principles of MR research: association, consistency, and independence. The MR-Egger method checked the externality of IVs by calculating the regression coefficient’s slope and intercept. If the slope is significantly nonzero, bias exists, indicating that the IV is correlated with the error term, necessitating correction using alternative methods. The weighted median method calculated the weighted median of all SNPs as the final causal estimate by multiplying each SNP’s effect size by its corresponding weight. This approach is advantageous in handling scenarios with numerous SNPs and has less impact on outliers. However, since weight selection can affect results, careful consideration is required when choosing appropriate weights. A *P*-value <.05 indicates statistical significance. To ensure the reliability of findings, sensitivity analyses (heterogeneity and pleiotropy tests), MR-Egger intercept test, leave-one-out test, and MR-PRESSO test were performed for comparison.

## 3. Results

### 3.1. Cross-sectional study by NHANES

In the NHANES 2007 to 2008 and 2015 to 2016 dataset, out of a total of 20,121 participants, after excluding those lacking data on OSA and related covariates, a total of 7980 participants were included in this study. Table 1 compares the baseline characteristics of the total sample, participants with OSA and those non-OSA. The results show significant differences (*P* < .001) between participants with OSA and non-OSA in terms of gender, education level, marital, BMI, hypertension, high cholesterol, diabetes, and coronary heart disease. Compared to others, individuals with OSA are more likely to be males under 60 years old, Non-Hispanic Black, have a 9th to 11th-grade education, be divorced, have a middle income, and have hypertension, high cholesterol, diabetes, and coronary heart disease.

Table 3 presents the logistic regression models for different regions of OSA and CKD. The results of the unadjusted model showed that with non-OSA as a reference, participants with OSA had a high risk of CKD (OR = 1.14, 95% CI: 1.01–1.28, *P* < .05). Furthermore, the trends remained the same after adjustment for age, gender, and race (model I). When further adjusted for age, gender, and race, education, PIR and marital (model II), the OR for participants with OSA was 1.20 and 95% CI: 1.06–1.36 (*P* < .05). However, in the model adjusted solely for comorbidities, this association was not significant (OR = 0.90, 95% CI: 0.79–1.02, *P* = .105). To further explore the role of hypertension in the relationship between OSA and CKD, we conducted a mediation analysis.

**Table 3:** The logistic regression analysis of the association between OSA and CKD.

	Crude model	Adjusted model 1	Adjusted model 2	Adjusted model 3
	OR (95% CI) *P*	OR (95% CI) *P*	OR (95% CI) *P*	OR (95% CI) *P*
OSA	1.14 (1.01–1.28)	1.18 (1.04–1.34)	1.20 (1.06–1.36)	0.90 (0.79–1.02)
	**.0292**	**.0087**	**.0044**	.1050

The bold text signifies a statistically significant distinction with a *P < *.05. Crude model: no covariates were adjusted. Adjusted model 1: AGE, GENDER, and RACE were adjusted. Adjusted model 2: AGE, GENDER, RACE, EDUCATION, PIR, and MARITAL were adjusted. Adjusted model 3: hypertension, high cholesterol, diabetes, coronary heart disease were adjusted.

CI = confidence intervals, CKD = chronic kidney disease, OR = odds ratio, OSA = obstructive sleep apnea, PIR = poverty income ratio.

**Figure 3.**
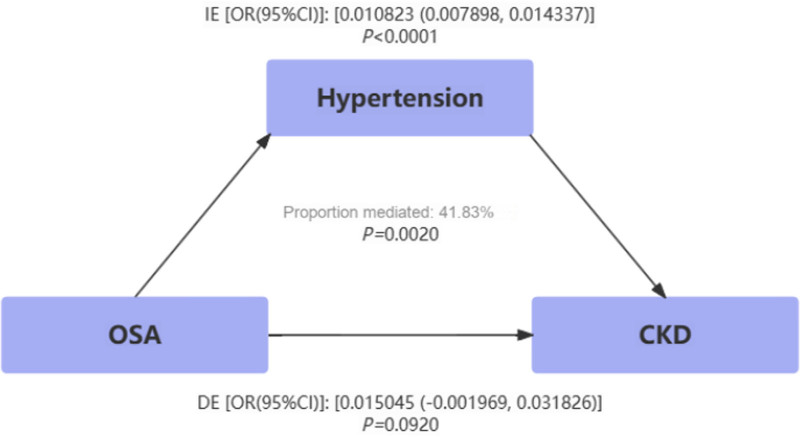
Estimated proportion of the association between OSA and CKD mediated by hypertension. CKD = chronic kidney disease, OSA = obstructive sleep apnea.

**Figure 4.**
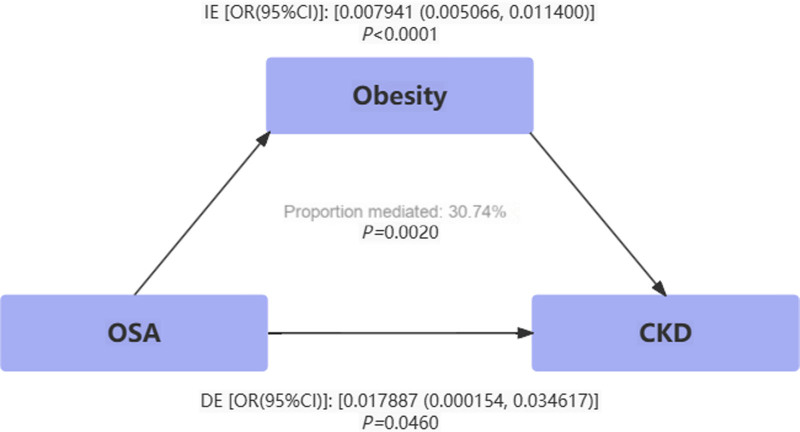
Estimated proportion of the association between OSA and CKD mediated by obesity. CKD = chronic kidney disease, OSA = obstructive sleep apnea.

The mediation analysis investigated whether and to what extent hypertension and obesity mediated the association between OSA and CKD. Table 4 shows the TE, which is the effect of OSA on CKD without considering the influence of the mediator; the DE, which is the effect of OSA on CKD, not mediated by hypertension or obesity; and the indirect effect, which is the effect of OSA on CKD, mediated by hypertension or obesity. In general, the DE greatly exceeded the indirect effect, although the statistical significance of the latter was significant. The proportion of hypertension and obesity mediating the effect of OSA on CKD was 41.83% and 30.74%, respectively (Figs. 3 and 4).

**Table 4:** Hypertension and obesity as mediators in the associations of OSA with CKD.

Mediation effect (OSA-hypertension/obesity-CKD)	Hypertension OR (95% CI)	*P*-value	Obesity OR (95% CI)	*P*-value
Total effect	0.026 (0.008–0.042)	**.0020**	0.026 (0.008–0.042)	**.0020**
Direct effect	0.015 (−0.002 to 0.032)	.0920	0.018 (0.000–0.035)	**.0460**
Indirect effect	0.011 (0.008–0.014)	**<.0001**	0.008 (0.005–0.011)	**<.0001**
Mediated (%)	41.83%	**.0020**	30.74%	**.0020**

The bold text signifies a statistically significant distinction with a *P* < .05. Model was adjusted for gender, age, race, education, marital, PIR; mediation proportion = IE/DE + IE.

CKD = chronic kidney disease, OSA = obstructive sleep apnea.

### 3.2. Univariate Mendelian randomization

#### 3.2.1. Causal relationship between OSA and renal impairment

**Figure 5.**
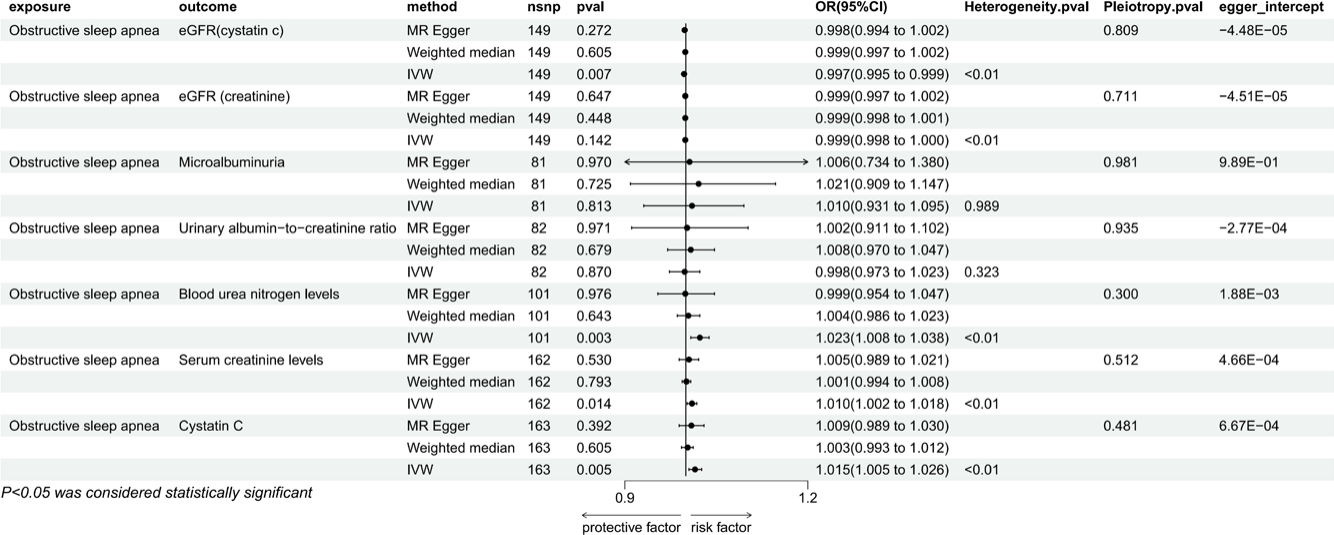
Mendelian randomization result of the effect of OSA on renal impairment. OSA = obstructive sleep apnea.

In the IVW method, genetically predicted OSA was associated with reduced eGFR_cystatin c_ levels (OR = 0.997, 95% CI: 0.995–0.999, *P* < .05). BUN levels (OR = 1.023, 95% CI: 1.008–1.038, *P* < .05), creatinine levels (OR = 1.010, 95% CI: 1.002–1.018, *P* < .05) and cystatin C levels (OR = 1.015, 95% CI: 1.005–1.026, *P* < .05) were associated with increased risk; but it was not significant in MR-Egger method and weighted median method (Fig. 5). In the sensitivity analysis, there was heterogeneity, but no pleiotropy, and the leave-one-out method also showed no SNPS that independently drove their causal relationship with renal impairment, the egger intercept did not deviate significantly from 0 (Supplementary document 2, Supplemental Digital Content, http://links.lww.com/MD/O350). The results of MR-PRESSO of them showed the presence of outlier SNPS, and the results were still significant after excluding the outlier SNPS (Supplementary document 3, Supplemental Digital Content, http://links.lww.com/MD/O351).

#### 3.2.2. Causal relationship between OSA and potential intermediaries

**Figure 6.**
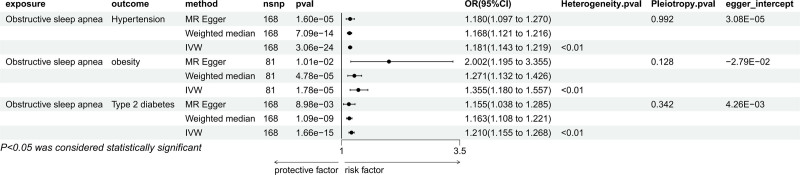
Mendelian randomization result of the effect of OSA on mediators. OSA = obstructive sleep apnea.

The IVW results showed that OSA was genetically predicted to be associated with obesity (OR = 1.355, 95% CI: 1.180–1.557, *P* < .05), hypertension (OR = 1.181, 95% CI: 1.143–1.219, *P* < .05) and T2DM (OR = 1.210, 95% CI: 1.155–1.268, *P* < .05). Moreover, they were still significant in MR-Egger method and weighted median method (Fig. 6). In the sensitivity analysis, there was no evidence of heterogeneity or pleiotropy. In the leave-one-out method of OSA to obesity, the results were still significant after the exclusion of the outlier snp rs937053, for hypertension and T2DM, the leave-one-out method also revealed that no SNP independently influenced their causal relationship (Supplementary document 2, Supplemental Digital Content, http://links.lww.com/MD/O350). The Egger intercept did not deviate significantly from 0. Additionally, the results of MR-PRESSO of them showed the presence of outlier SNPS, and the results were still significant after excluding the outlier SNPS (Supplementary document 3, Supplemental Digital Content, http://links.lww.com/MD/O351).

#### 3.2.3. Causal relationship between potential mediators and positive outcome

**Figure 7.**
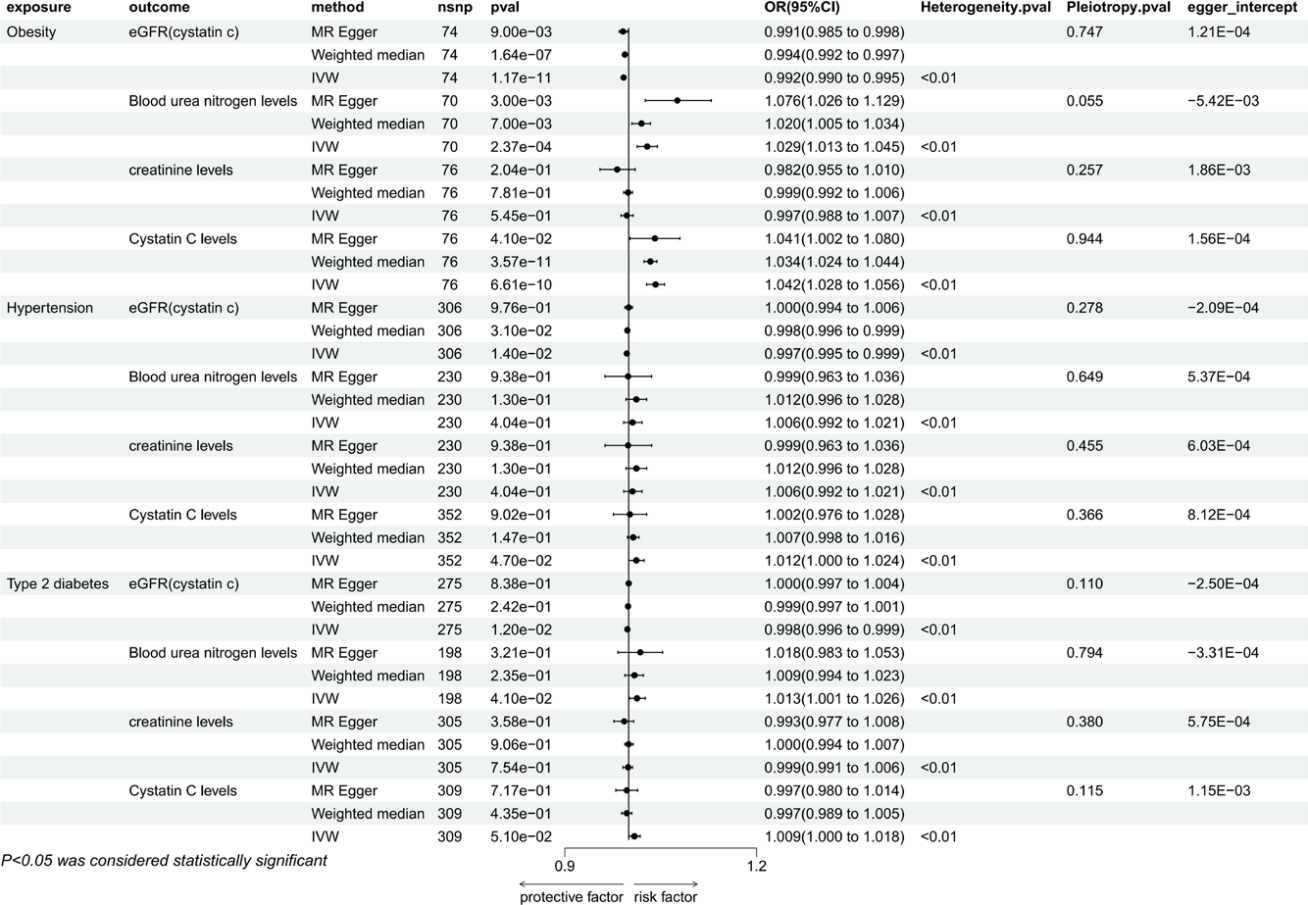
Mendelian randomization result of the effect of mediators (hypertension, T2DM, and obesity) on positive outcomes. T2DM = Type 2 diabetes mellitus.

IVW results showed that genetic prediction of obesity was associated with eGFR_cystatin c_ (OR = 0.992, 95% CI: 0.990–0.995, *P* < .05), BUN levels (OR = 1.029, 95% CI: 1.013–1.045, *P* < .05), and cystatin C levels (OR = 1.042, 95% CI: 1.028–1.056, *P* < .05) and these causal relationships were also verified in the other 2 methods. The causal relationship between hypertension and eGFR_cystatin c_ was significant in both the IVW method (OR = 0.997, 95% CI: 0.995–0.999, *P* < .05) and the weighted median method (OR = 0.998, 95% CI: 0.996–0.999, *P* < .05), but not in the MR-Egger method, and the causal relationship of cystatin C levels were significantly correlated in IVW method (OR = 1.012, 95%CI: 1.000–1.024, *P* < .05). In IVW method, there was a causal relationship between T2DM and eGFR_cystatin c_ (OR = 0.998, 95% CI: 0.996–0.999, *P* < .05) and BUN levels (OR = 1.013, 95% CI: 1.001–1.026, *P* < .05), but it was not significant in the weighted median method and MR-Egger method (Fig. 7). In the sensitivity analysis, there was no indication of heterogeneity or pleiotropy. The leave-one-out method also indicated that no SNP had a significant independent effect on their causal relationship with positive exposure (Supplemental Digital Content 2, http://links.lww.com/MD/O350). Furthermore, the Egger intercept did not deviate significantly from 0. For the causal relationship between T2DM and eGFR_cystatin c_, in MR-PRESSO method, the causal relationship between them disappeared after the removal of outliers, so follow-up MVMR analysis was not performed, but for other results of MR-PRESSO showed the presence of outlier SNPS, and the results were still significant after excluding the outlier SNPS (Supplemental Digital Content 3, http://links.lww.com/MD/O351).

### 3.3. Multivariate Mendelian randomization

#### 3.3.1. Mediating effects of obesity in the association between OSA and renal impairment

**Figure 8.**
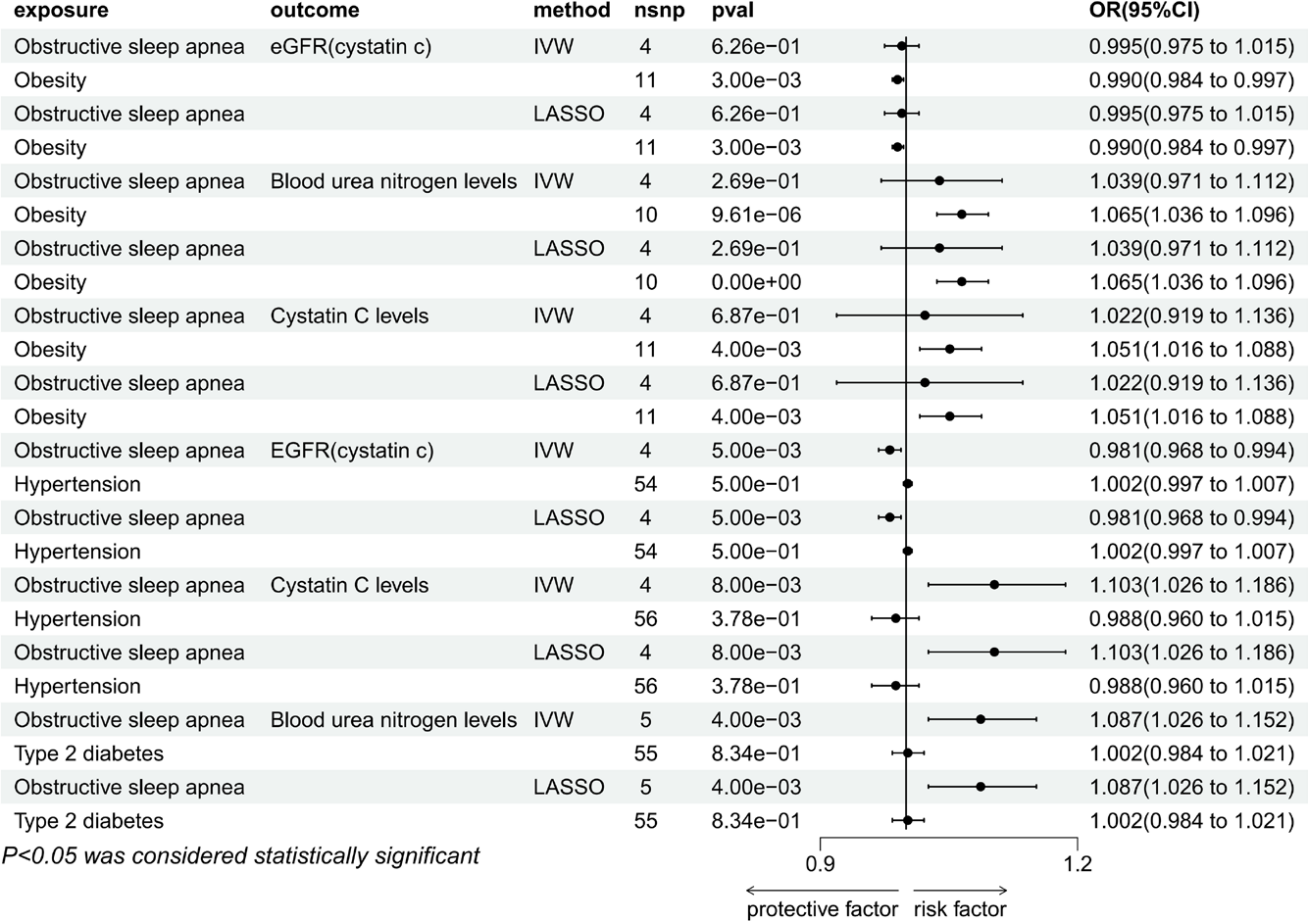
Multivariable MR result of causal relationships of OSA and mediators (hypertension, T2DM, and obesity) on renal impairment. MR = Mendelian randomization, OSA = obstructive sleep apnea, T2DM = type 2 diabetes mellitus.

In the IVW approach, after adjusting for obesity, the casual relationship of OSA and eGFR_cystatin c_ (OR = 0.995, 95% CI: 0.975–1.015, *P* = .626), BUN levels (OR = 1.039, 95% CI: 0.971–1.112, *P* = .269), and cystatin C levels (OR = 1.022, 95% CI: 0.919–1.136, *P* = .687) disappeared. On the contrary, obesity was still associated with eGFR_cystatin c_ (OR = 0.990, 95% CI: 0.984–0.997, *P* < .05), BUN levels (OR = 1.065, 95% CI: 1.036–1.096, *P* < .05), and cystatin C levels (OR = 1.051, 95% CI: 1.016–1.088, *P* < .05), the same result was also shown in LASSO (Fig. 8).

#### 3.3.2. Mediating effects of hypertension in the association between OSA and renal impairment

After adjusting for hypertension, the link of hypertension and eGFR_cystatin c_ (OR = 1.002, 95%CI: 0.997–1.007, *P* = .500) and cystatin C levels (OR = 0.988, 95% CI: 0.960–1.015, *P* = .378) lost statistical significance. However, the association between OSA and eGFR_cystatin c_ (OR = 0.981, 95% CI: 0.968–0.994, *P* < .05) and cystatin C levels (OR = 1.103, 95% CI: 1.026–1.186, *P* < .05) remained statistically significant even after adjusting for hypertension, as illustrated in Figure 8.

#### 3.3.3. Mediating effects of T2DM in the association between OSA and renal impairment

After adjusting for T2DM, the relationship between OSA and BUN levels (OR = 1.087, 95% CI: 1.026–1.152, *P* < .05) was still significant. However, the causal relationship of T2DM on BUN levels (OR = 1.002, 95% CI: 0.984–1.021, *P* = .834) was disappeared, the same results were both obtained in LASSO. These results indicated that the causal relationship between OSA and renal impairment was not affected by T2DM (Fig. 8).

## 4. Discussion

To our knowledge, this is the first study to combine a cross-sectional study with MR analysis to investigate the association between OSA and CKD. The cross-sectional results suggested a positive relationship between OSA and CKD, mediated by hypertension and obesity. The 2-sample MR indicated a potential causal association between OSA and renal impairment, and the MVMR results demonstrated that obesity mediates the causal relationship between OSA and several measures of renal impairment, such as eGFR_cystatin c_, BUN, and serum cystatin C.

This cross-sectional study shows that compared to individuals without OSA, those with OSA are more likely to develop CKD. A significant positive correlation between OSA and CKD was observed across 3 different models. As the risk of OSA increased, the prevalence of CKD also rose. MR analysis further confirmed this conclusion, indicating that OSA is a potential risk factor for renal impairment. Consistent with previous research, individuals with OSA often exhibited more severe renal impairment compared to healthy populations. However, the underlying mechanisms mediating this association remain uncertain. A retrospective study found a significantly higher incidence of CKD in individuals with moderate to severe OSA compared to those with no or mild OSA.^[8]^ Conversely, another retrospective cohort study did not find an association between the severity of OSA and renal impairment, particularly in eGFR.^[18]^ Differences in outcomes may be attributed to mild OSA patients often being classified as non-OSA individuals, which is easily overlooked. Additionally, we found that after including comorbidities in the adjusted model, the association between OSA and CKD disappeared. This may be related to the limited awareness of underlying conditions during the 2 survey cycles, suggesting there may still be a substantial undiagnosed population.^[19]^

This MR study revealed a potential causal relationship between genetic predisposition to OSA and decreased eGFR_cystatin c_, increased BUN, increased serum creatinine, and higher levels of serum cystatin C – indicators reflecting severity of renal impairment. GFR was a crucial parameter determining kidney function, reflecting the kidney’s ability to eliminate certain substances from the bloodstream, and it could be estimated based on levels of serum creatinine or serum cystatin C.^[20,21]^ Cystatin C, being a non-glycosylated protein with a relatively stable concentration unaffected by factors such as gender, age, or muscle mass, was considered an ideal endogenous marker reflecting changes in GFR.^[22,23]^ The previous MR confirmed this viewpoint.^[24]^ Therefore, eGFR_cystatin c_ may be a more accurate indicator of renal function. At the same time, more alternative indicators were needed to supplement and verify renal function. BUN and serum creatinine were major end products of protein metabolism, and their excretion depended on the kidneys.^[25]^ When the GFR dropped below 50% of normal, BUN and serum creatinine concentrations rapidly increase. However, BUN and serum creatinine had lower sensitivity and were often used as supplementary indicator in assessing kidney function.^[26]^

We were the first to investigate the mediating relationship between OSA and CKD, finding that hypertension and obesity may mediate this association, accounting for 41.83% and 30.74%, respectively. Currently, there was no consensus on the mechanistic explanation for the association between OSA and CKD. The 2 most convincing explanations currently were hypoxia and hypertension.^[27,28]^ Hypoxia has been identified as a key process through which OSA led to CKD. Renal hypoxia was determined by a reduction in renal perfusion and an increase in oxygen consumption by renal tissue.^[29]^ However, owing to the unique structure and organization of renal arterioles and venules in the cortex and medulla, renal oxygen consumption constituted only 10% of the total body oxygen consumption.^[30]^ Therefore, the key to renal hypoxia was the decrease in blood oxygen concentration caused by OSA. Furthermore, hypoxia induced epithelial-to-mesenchymal transition in renal tubular cells, activates fibroblasts, and led to interstitial fibrosis and tubulointerstitial damage, which were considered common pathways in the progression to end-stage renal failure.^[31]^ In the NHANES study, we found that the mediating effect of hypertension was 41.83%. However, MVMR results revealed that the association between OSA adjusted for hypertension and renal impairment disappeared. It was noteworthy that OSA through the activation of the renin-angiotensin system, excessive sympathetic nerve activation, and the impact on endothelial function, led to hypertension, subsequently impairing renal function.^[32–34]^ This represented an indirect influence. Additionally, the different racial compositions in the 2 databases may also contribute to bias. However, the absence of relevant databases prevented us from exploring the causative relationships within this context. In the future, we aim to enhance the available genetic data, laying a solid foundation for subsequent research.

In this study, we combined NHANES data with MR analysis to demonstrate that obesity was a mediating factor in the relationship between OSA and CKD. In MR analysis, genetic predisposition to OSA was associated with decreased eGFR_cystatin c_, increased BUN and serum cystatin C levels in individuals with obesity. At the genetic level, the aforementioned associations were driven by OSA SNPs that were correlated with obesity, suggesting that the pathway from OSA to renal impairment may be mediated through obesity. Mechanistically, individuals with OSA often experienced nocturnal intermittent hypoxia, leading to disruptions in the secretion of hormones such as leptin and subsequently resulting in increased appetite and obesity.^[27,35]^ Obesity was recognized as a risk factor for renal impairment, although the specific mechanisms remained inconclusive.^[36,37]^ Research indicated that an increase in adipose tissue could elevate circulating levels of molecules such as leptin and angiotensin II, impacting renal tubular cells and mesangial cells, thereby exacerbating renal impairment.^[38]^ A retrospective cross-sectional study showed that compared to the normal weight population, obese snoring patients had a nearly doubled risk of CKD (OR: 2.51).^[39]^

Our study has several strengths. To the best of our knowledge, this is the first study to combine a cross-sectional study with MR analysis to investigate the association between OSA and CKD. Previous studies explored the relationship between OSA and CKD in U.S. populations. Their findings were limited by sample size, raising questions about generalizability. NHANES, a nationwide survey conducted with rigorous quality control procedures, provided clinically relevant and highly generalizable findings. Additionally, we adjusted for multiple confounding factors, including age, gender, race, education, PIR, and marital, enhancing our results’ robustness. Unlike other studies, we also demonstrated a genetic association between OSA and renal impairment. This may offer clinicians more precise indicators for OSA patients. These findings hold significant implications for health care. Both OSA and CKD are associated with fatigue and cognitive decline,^[40–42]^ contributing to increased physical and psychological burdens on individuals. Furthermore, the high prevalence of these conditions places a substantial economic and healthcare resource burden. Our study highlights the importance of CKD screening in OSA patients, particularly obesity and hypertension patients. Additionally, there was a need for the development of more rational weight and hypertension management strategies, as they may potentially slow down renal impairment progression and improve patients’ life quality.

However, there were some limitations. First, we diagnosed OSA based on typical symptoms found in the NHANES questionnaire, such as daytime sleepiness, apnea, and snoring. However, the subjective self-reporting in the questionnaire introduces bias, as it is not as precise as diagnosis through sleep apnea monitoring. In this study, the prevalence of OSA among Americans under 60 was 31.13%, which is significantly higher than the global prevalence of OSA.^[5]^ Second, the influence of OSA on CKD via mediators like hypertension and obesity is primarily based on statistical models. This requires further validation through animal studies to clarify the underlying mechanisms. Third, in order to avoid the bias of population heterogeneity, we only based the GWAS summary statistics on the population of European descent, and whether these results were applicable to other ethnic groups needs to be further explored. Third, in order to obtain sufficient instrumental variables, IV selected a *P*-value threshold of 5 × 10^−05^ to compensate for the lack of SNPS with effect P-values lower than conventional genome-wide significance (*P* < 5 × 10^−8^), but this may introduce weak instrument bias to the overall estimate.

In conclusion, this study combined NHANES data with MR analysis to elaborate on the causal impact of OSA on the risk of CKD. It adds causal evidence to the etiology of CKD and identifies prevention and intervention targets to mitigate renal impairment and its related disease burden. Obesity and hypertension may mediate the pathway from OSA to CKD. These findings may inform strategies for preventing and managing CKD.

## Author contributions

**Conceptualization:** Shaokang Wang.

**Formal analysis:** Mi Ou, Tianlong Yin, Yalu Meng, Haipeng Ban, Wenlong Gu, Xianggang Meng, Lili Zhang.

**Funding acquisition:** Yuzheng Du.

**Methodology:** Yupei Cheng, Zhe Zhang.

**Resources:** Yuzheng Du.

**Supervision:** Yuzheng Du.

**Writing – original draft:** Shaokang Wang, Yupei Cheng, Zhe Zhang.

**Writing – review & editing:** Wei Liu, Mi Ou, Tianlong Yin, Yalu Meng, Haipeng Ban, Wenlong Gu, Xianggang Meng, Lili Zhang.

## Supplementary Material


