# Supplementary material for: Association between obstructive sleep apnea and chronic kidney disease: A cross-sectional and Mendelian randomization study
Source: Medicine (Baltimore). 2025 Feb 7;104(6):e41437. doi: 10.1097/MD.0000000000041437 (PMC11812998; doi:10.1097/MD.0000000000041437)
Supplement: Supplementary file 1 [file medi-104-e41437-s001.pdf]

Supplementary document 1

Adjust P-values and LD raw data-hypertension

	chr.exposure	pos.exposure	beta.exposure	se.exposure	pval.exposure	id.exposure	SNP	effect_allele.exposure	other_allele.exposure	eaf.exposure	exposure	mr_keep.exposure	pval_origin.exposure	data_source.exposure	F statistics
1	1	6652718	0.0443	0.0096	4.20301E-06	finn-b-I9_HYPTENS	rs2235567	A	G	0.34	|| id:finn-b-I9_HYPTENS	TRUE	reported	igd	21.29437934
2	1	10798489	0.1033	0.0093	1.04304E-28	finn-b-I9_HYPTENS	rs34071855	G	C	0.415	|| id:finn-b-I9_HYPTENS	TRUE	reported	igd	123.3771534
3	1	156160266	0.0669	0.0163	0.00003879	finn-b-I9_HYPTENS	rs12756315	T	G	0.08744	|| id:finn-b-I9_HYPTENS	TRUE	reported	igd	16.84523317
4	1	62020058	0.1195	0.0273	1.24701E-05	finn-b-I9_HYPTENS	rs11207770	G	A	0.02884	|| id:finn-b-I9_HYPTENS	TRUE	reported	igd	19.16066229
5	1	208249359	-0.0517	0.0125	3.27997E-05	finn-b-I9_HYPTENS	rs6659931	T	C	0.84	|| id:finn-b-I9_HYPTENS	TRUE	reported	igd	17.106496
6	1	210029185	0.1914	0.0463	3.52501E-05	finn-b-I9_HYPTENS	rs11580973	A	C	0.00981	|| id:finn-b-I9_HYPTENS	TRUE	reported	igd	17.089206
7	1	113046879	0.1321	0.0121	1.40314E-27	finn-b-I9_HYPTENS	rs3790604	A	C	0.1709	|| id:finn-b-I9_HYPTENS	TRUE	reported	igd	119.1886483
8	1	115788614	0.2088	0.0412	4.062E-07	finn-b-I9_HYPTENS	rs150863140	A	G	0.01242	|| id:finn-b-I9_HYPTENS	TRUE	reported	igd	25.68423037
9	1	3327032	0.0701	0.0147	1.78999E-06	finn-b-I9_HYPTENS	rs2493296	T	C	0.109	|| id:finn-b-I9_HYPTENS	TRUE	reported	igd	22.74057106
10	1	22302082	-0.0493	0.0117	0.00002362	finn-b-I9_HYPTENS	rs11591033	C	T	0.1899	|| id:finn-b-I9_HYPTENS	TRUE	reported	igd	17.75505881
11	1	27485635	-0.1266	0.0295	1.74799E-05	finn-b-I9_HYPTENS	rs191328307	A	G	0.02487	|| id:finn-b-I9_HYPTENS	TRUE	reported	igd	18.41719046
12	1	179527183	0.0402	0.0094	2.01502E-05	finn-b-I9_HYPTENS	rs6425577	T	C	0.6258	|| id:finn-b-I9_HYPTENS	TRUE	reported	igd	18.28927116
13	1	195634961	-0.1137	0.0248	4.79104E-06	finn-b-I9_HYPTENS	rs76059172	C	G	0.0355	|| id:finn-b-I9_HYPTENS	TRUE	reported	igd	21.01926704
14	1	228406455	0.0506	0.0096	1.46899E-07	finn-b-I9_HYPTENS	rs7521297	G	A	0.3403	|| id:finn-b-I9_HYPTENS	TRUE	reported	igd	27.78168403
15	1	230873488	0.0701	0.0112	4.03999E-10	finn-b-I9_HYPTENS	rs1317181	T	G	0.2099	|| id:finn-b-I9_HYPTENS	TRUE	reported	igd	39.17418686
16	1	51123315	-0.0892	0.0217	3.98896E-05	finn-b-I9_HYPTENS	rs12117653	A	C	0.04675	|| id:finn-b-I9_HYPTENS	TRUE	reported	igd	16.89702478
17	1	148547251	0.2005	0.0427	2.65699E-06	finn-b-I9_HYPTENS	rs146988330	A	T	0.98454	|| id:finn-b-I9_HYPTENS	TRUE	reported	igd	22.0481931
18	1	150409850	-0.104	0.0228	5.05697E-06	finn-b-I9_HYPTENS	rs116140174	G	C	0.04227	|| id:finn-b-I9_HYPTENS	TRUE	reported	igd	20.80640197
19	1	204397901	-0.098	0.0241	4.69602E-05	finn-b-I9_HYPTENS	rs61758011	A	G	0.03734	|| id:finn-b-I9_HYPTENS	TRUE	reported	igd	16.53552797
20	1	27260783	0.098	0.0176	2.73602E-08	finn-b-I9_HYPTENS	rs7545442	T	C	0.07246	|| id:finn-b-I9_HYPTENS	TRUE	reported	igd	31.00464876
21	1	48064052	0.069	0.0143	1.34999E-06	finn-b-I9_HYPTENS	rs11211570	C	A	0.1169	|| id:finn-b-I9_HYPTENS	TRUE	reported	igd	23.28231209
22	1	57900404	0.0525	0.011	1.85498E-06	finn-b-I9_HYPTENS	rs11806059	A	C	0.2207	|| id:finn-b-I9_HYPTENS	TRUE	reported	igd	22.77892562
23	1	217739036	0.0449	0.0107	2.66502E-05	finn-b-I9_HYPTENS	rs2042525	C	T	0.238	|| id:finn-b-I9_HYPTENS	TRUE	reported	igd	17.60861211
24	1	89441642	0.0464	0.0093	6.24195E-07	finn-b-I9_HYPTENS	rs12120164	G	A	0.4017	|| id:finn-b-I9_HYPTENS	TRUE	reported	igd	24.89258874
25	1	25136203	0.0668	0.0111	1.865E-09	finn-b-I9_HYPTENS	rs6668768	T	C	0.2144	|| id:finn-b-I9_HYPTENS	TRUE	reported	igd	36.21654087
26	1	11981282	-0.1373	0.0175	5.00956E-15	finn-b-I9_HYPTENS	rs143439093	G	A	0.07436	|| id:finn-b-I9_HYPTENS	TRUE	reported	igd	61.55523265
27	1	39969059	0.0467	0.0109	1.94899E-05	finn-b-I9_HYPTENS	rs72663503	T	C	0.2253	|| id:finn-b-I9_HYPTENS	TRUE	reported	igd	18.35611481
28	1	118268561	0.0399	0.0092	1.32401E-05	finn-b-I9_HYPTENS	rs10923405	C	G	0.4654	|| id:finn-b-I9_HYPTENS	TRUE	reported	igd	18.80919187
29	1	157069134	0.1614	0.0368	1.12001E-05	finn-b-I9_HYPTENS	rs61730132	A	G	0.01592	|| id:finn-b-I9_HYPTENS	TRUE	reported	igd	19.23585184
30	1	56944045	0.0392	0.0093	2.49597E-05	finn-b-I9_HYPTENS	rs11206825	G	A	0.4072	|| id:finn-b-I9_HYPTENS	TRUE	reported	igd	17.76667823
31	2	61889346	0.0606	0.0117	2.20999E-07	finn-b-I9_HYPTENS	rs78185579	C	G	0.1892	|| id:finn-b-I9_HYPTENS	TRUE	reported	igd	26.82708744
32	2	65267950	-0.2321	0.0545	2.08901E-05	finn-b-I9_HYPTENS	rs138843384	T	G	0.007323	|| id:finn-b-I9_HYPTENS	TRUE	reported	igd	18.13665853
33	2	79333115	0.1529	0.0365	2.80602E-05	finn-b-I9_HYPTENS	rs535999138	T	C	0.01588	|| id:finn-b-I9_HYPTENS	TRUE	reported	igd	17.5480653
34	2	37047651	-0.0428	0.0096	8.86503E-06	finn-b-I9_HYPTENS	rs2110882	G	A	0.649	|| id:finn-b-I9_HYPTENS	TRUE	reported	igd	19.87673611
35	2	156622426	0.0446	0.0105	2.34698E-05	finn-b-I9_HYPTENS	rs142839853	G	T	0.2497	|| id:finn-b-I9_HYPTENS	TRUE	reported	igd	18.04226757
36	2	164447403	0.0587	0.0107	3.539E-08	finn-b-I9_HYPTENS	rs10185395	T	G	0.243	|| id:finn-b-I9_HYPTENS	TRUE	reported	igd	30.09599092
37	2	43165179	-0.0747	0.0171	1.29999E-05	finn-b-I9_HYPTENS	rs80258496	A	C	0.07757	|| id:finn-b-I9_HYPTENS	TRUE	reported	igd	19.08310249
38	2	104037903	-0.5549	0.1282	0.00001501	finn-b-I9_HYPTENS	rs13421017	G	C	0.001387	|| id:finn-b-I9_HYPTENS	TRUE	reported	igd	18.73498714
39	2	141587228	0.1431	0.0331	1.55299E-05	finn-b-I9_HYPTENS	rs2380897	C	T	0.01938	|| id:finn-b-I9_HYPTENS	TRUE	reported	igd	18.69060158
40	2	164999883	-0.0603	0.0092	6.94704E-11	finn-b-I9_HYPTENS	rs1374264	C	A	0.4146	|| id:finn-b-I9_HYPTENS	TRUE	reported	igd	42.95947543
41	2	242212773	-0.1055	0.0232	5.64898E-06	finn-b-I9_HYPTENS	rs78615520	G	C	0.04084	|| id:finn-b-I9_HYPTENS	TRUE	reported	igd	20.67897221
42	2	161662617	0.0497	0.0119	2.84898E-05	finn-b-I9_HYPTENS	rs489354	A	G	0.821	|| id:finn-b-I9_HYPTENS	TRUE	reported	igd	17.44290657
43	2	183093601	-0.0579	0.0136	2.09701E-05	finn-b-I9_HYPTENS	rs9784041	T	C	0.1302	|| id:finn-b-I9_HYPTENS	TRUE	reported	igd	18.12505407
44	2	227077377	0.0457	0.0096	2.01799E-06	finn-b-I9_HYPTENS	rs2943635	T	C	0.6574	|| id:finn-b-I9_HYPTENS	TRUE	reported	igd	22.66156684
45	2	241378339	-0.101	0.0245	3.70502E-05	finn-b-I9_HYPTENS	rs76959112	T	C	0.0363	|| id:finn-b-I9_HYPTENS	TRUE	reported	igd	16.99458559
46	2	121232720	-0.1065	0.0249	1.89701E-05	finn-b-I9_HYPTENS	rs74752894	A	G	0.03876	|| id:finn-b-I9_HYPTENS	TRUE	reported	igd	18.29365655
47	2	131013592	-0.2554	0.0596	0.00001798	finn-b-I9_HYPTENS	rs150315199	G	A	0.006332	|| id:finn-b-I9_HYPTENS	TRUE	reported	igd	18.36323814
48	2	119753196	0.0566	0.0135	0.0000277	finn-b-I9_HYPTENS	rs62157413	T	G	0.1346	|| id:finn-b-I9_HYPTENS	TRUE	reported	igd	17.57783265
49	2	134465553	0.1048	0.0239	0.00001181	finn-b-I9_HYPTENS	rs140271295	A	G	0.03757	|| id:finn-b-I9_HYPTENS	TRUE	reported	igd	19.22767459
50	2	45873914	0.0484	0.0092	1.47401E-07	finn-b-I9_HYPTENS	rs568943	A	C	0.5694	|| id:finn-b-I9_HYPTENS	TRUE	reported	igd	27.67674858
51	2	79794859	0.0471	0.0101	2.88098E-06	finn-b-I9_HYPTENS	rs1367400	G	C	0.707	|| id:finn-b-I9_HYPTENS	TRUE	reported	igd	21.74698559
52	2	290003	-0.042	0.01	2.83198E-05	finn-b-I9_HYPTENS	rs75141812	T	C	0.3008	|| id:finn-b-I9_HYPTENS	TRUE	reported	igd	17.64
53	2	8833100	0.1998	0.0482	3.36202E-05	finn-b-I9_HYPTENS	rs79287785	A	G	0.009175	|| id:finn-b-I9_HYPTENS	TRUE	reported	igd	17.18291696
54	2	202859663	-0.0546	0.0117	2.86999E-06	finn-b-I9_HYPTENS	rs13015399	A	C	0.19	|| id:finn-b-I9_HYPTENS	TRUE	reported	igd	21.77777778
55	2	182121130	0.0486	0.0097	4.93401E-07	finn-b-I9_HYPTENS	rs1371617	G	A	0.6628	|| id:finn-b-I9_HYPTENS	TRUE	reported	igd	25.10319906
56	2	209697637	-0.377	0.0898	2.65797E-05	finn-b-I9_HYPTENS	rs138241581	T	C	0.002755	|| id:finn-b-I9_HYPTENS	TRUE	reported	igd	17.62503658
57	2	43381248	-0.0429	0.0095	6.39499E-06	finn-b-I9_HYPTENS	rs11674418	A	G	0.6412	|| id:finn-b-I9_HYPTENS	TRUE	reported	igd	20.39235457
58	2	26911509	-0.0953	0.0092	3.37987E-25	finn-b-I9_HYPTENS	rs1275984	C	A	0.549	|| id:finn-b-I9_HYPTENS	TRUE	reported	igd	107.3025756
59	2	80194080	-0.0433	0.0095	4.70403E-06	finn-b-I9_HYPTENS	rs13001262	T	C	0.6259	|| id:finn-b-I9_HYPTENS	TRUE	reported	igd	20.77440443
60	2	159462894	-0.079	0.0134	3.56098E-09	finn-b-I9_HYPTENS	rs10206833	A	G	0.8661	|| id:finn-b-I9_HYPTENS	TRUE	reported	igd	34.75718423
61	2	180684788	-0.0441	0.0094	2.99999E-06	finn-b-I9_HYPTENS	rs55869281	A	G	0.3727	|| id:finn-b-I9_HYPTENS	TRUE	reported	igd	22.01007243
62	3	14536101	-0.1297	0.0314	3.62802E-05	finn-b-I9_HYPTENS	rs73031848	A	G	0.02206	|| id:finn-b-I9_HYPTENS	TRUE	reported	igd	17.06163536
63	3	2167184	0.0986	0.022	7.34497E-06	finn-b-I9_HYPTENS	rs12637200	G	T	0.04512	|| id:finn-b-I9_HYPTENS	TRUE	reported	igd	20.08669421
64	3	48225509	0.0484	0.0097	6.35594E-07	finn-b-I9_HYPTENS	rs3731503	C	T	0.33	|| id:finn-b-I9_HYPTENS	TRUE	reported	igd	24.8970135
65	3	27562988	0.0627	0.0093	1.75106E-11	finn-b-I9_HYPTENS	rs2643826	T	C	0.4047	|| id:finn-b-I9_HYPTENS	TRUE	reported	igd	45.45369407
66	3	123824333	0.0653	0.0134	0.000001024	finn-b-I9_HYPTENS	rs16835100	T	C	0.1355	|| id:finn-b-I9_HYPTENS	TRUE	reported	igd	23.74743818
67	3	183938857	0.0459	0.0105	1.20701E-05	finn-b-I9_HYPTENS	rs4447797	A	G	0.2581	|| id:finn-b-I9_HYPTENS	TRUE	reported	igd	19.10938776
68	3	113542122	0.0425	0.0093	4.51596E-06	finn-b-I9_HYPTENS	rs9647328	G	A	0.5378	|| id:finn-b-I9_HYPTENS	TRUE	reported	igd	20.88391722
69	3	104585106	0.0417	0.0102	4.07099E-05	finn-b-I9_HYPTENS	rs28697231	A	G	0.2791	|| id:finn-b-I9_HYPTENS	TRUE	reported	igd	16.71366782
70	3	168774754	-0.1096	0.0192	1.064E-08	finn-b-I9_HYPTENS	rs16853076	C	T	0.06089	|| id:finn-b-I9_HYPTENS	TRUE	reported	igd	32.58506944
71	3	185613214	-0.0437	0.0095	4.27002E-06	finn-b-I9_HYPTENS	rs60760789	T	C	0.6262	|| id:finn-b-I9_HYPTENS	TRUE	reported	igd	21.16
72	3	169104295	-0.0595	0.0113	1.55901E-07	finn-b-I9_HYPTENS	rs9859964	T	C	0.2044	|| id:finn-b-I9_HYPTENS	TRUE	reported	igd	27.72535046
73	3	167697355	0.1197	0.0243	8.22906E-07	finn-b-I9_HYPTENS	rs181836203	A	C	0.03751	|| id:finn-b-I9_HYPTENS	TRUE	reported	igd	24.26474623
74	3	133887637	0.0469	0.0091	2.844E-07	finn-b-I9_HYPTENS	rs7623609	T	G	0.5363	|| id:finn-b-I9_HYPTENS	TRUE	reported	igd	26.56213018
75	3	14894140	0.0984	0.0153	1.29799E-10	finn-b-I9_HYPTENS	rs4685218	T	C	0.09873	|| id:finn-b-I9_HYPTENS	TRUE	reported	igd	41.36255286
76	3	19791914	0.1101	0.0231	1.93602E-06	finn-b-I9_HYPTENS	rs114004624	G	C	0.04113	|| id:finn-b-I9_HYPTENS	TRUE	reported	igd	22.71698431
77	3	28828443	0.1455	0.0315	3.95003E-06	finn-b-I9_HYPTENS	rs116275484	A	T	0.02141	|| id:finn-b-I9_HYPTENS	TRUE	reported	igd	21.33560091
78	3	29373274	-0.0477	0.0103	3.33403E-06	finn-b-I9_HYPTENS	rs7374763	G	A	0.2747	|| id:finn-b-I9_HYPTENS	TRUE	reported	igd	21.44679046
79	3	53558012	0.0467	0.0098	1.72401E-06	finn-b-I9_HYPTENS	rs3821843	A	G	0.6592	|| id:finn-b-I9_HYPTENS	TRUE	reported	igd	22.70814244
80	3	134845693	0.0748	0.0152	8.7259E-07	finn-b-I9_HYPTENS	rs7625747	G	T	0.1001	|| id:finn-b-I9_HYPTENS	TRUE	reported	igd	24.216759
81	3	158214203	0.0419	0.0094	8.22205E-06	finn-b-I9_HYPTENS	rs67286798	A	G	0.3827	|| id:finn-b-I9_HYPTENS	TRUE	reported	igd	19.86883205
82	4	40609348	0.0633	0.0129	8.90697E-07	finn-b-I9_HYPTENS	rs11933597	T	C	0.148	|| id:finn-b-I9_HYPTENS	TRUE	reported	igd	24.07842077
83	4	106911742	0.0697	0.0121	9.34308E-09	finn-b-I9_HYPTENS	rs13112725	C	G	0.8285	|| id:finn-b-I9_HYPTENS	TRUE	reported	igd	33.18140837
84	4	185877562	-0.1328	0.0222	2.27002E-09	finn-b-I9_HYPTENS	rs149150643	C	T	0.04503	|| id:finn-b-I9_HYPTENS	TRUE	reported	igd	35.78410843
85	4	81182554	0.1157	0.0099	1.3369E-31	finn-b-I9_HYPTENS	rs12509595	C	T	0.3122	|| id:finn-b-I9_HYPTENS	TRUE	reported	igd	136.5828997
86	4	143770179	0.0463	0.0102	4.99505E-06	finn-b-I9_HYPTENS	rs4690730	C	T	0.2814	|| id:finn-b-I9_HYPTENS	TRUE	reported	igd	20.60447905
87	4	83779494	-0.0599	0.0123	0.000001048	finn-b-I9_HYPTENS	rs35180881	T	C	0.1675	|| id:finn-b-I9_HYPTENS	TRUE	reported	igd	23.71610814
88	4	156445180	-0.0578	0.012	1.57699E-06	finn-b-I9_HYPTENS	rs7660191	C	T	0.8265	|| id:finn-b-I9_HYPTENS	TRUE	reported	igd	23.20027778
89	4	26796406	-0.0532	0.0105	3.721E-07	finn-b-I9_HYPTENS	rs73249866	G	A	0.2598	|| id:finn-b-I9_HYPTENS	TRUE	reported	igd	25.67111111
90	4	48991540	-0.0481	0.0103	3.10599E-06	finn-b-I9_HYPTENS	rs936135	A	G	0.7317	|| id:finn-b-I9_HYPTENS	TRUE	reported	igd	21.80799321
91	4	111386198	-0.0548	0.0115	1.99802E-06	finn-b-I9_HYPTENS	rs6848906	C	T	0.8069	|| id:finn-b-I9_HYPTENS	TRUE	reported	igd	22.70729679
92	4	86709899	0.0518	0.0097	8.16601E-08	finn-b-I9_HYPTENS	rs7662174	G	T	0.3381	|| id:finn-b-I9_HYPTENS	TRUE	reported	igd	28.5178021
93	4	156639174	-0.0751	0.0096	4.48126E-15	finn-b-I9_HYPTENS	rs3796585	A	G	0.3465	|| id:finn-b-I9_HYPTENS	TRUE	reported	igd	61.19802517
94	4	6142049	-0.085	0.0173	8.82409E-07	finn-b-I9_HYPTENS	rs16838311	G	C	0.08548	|| id:finn-b-I9_HYPTENS	TRUE	reported	igd	24.14046577
95	4	38359317	-0.0385	0.0093	3.61501E-05	finn-b-I9_HYPTENS	rs6819003	A	G	0.4244	|| id:finn-b-I9_HYPTENS	TRUE	reported	igd	17.1378194
96	4	108940029	0.0786	0.0189	3.19602E-05	finn-b-I9_HYPTENS	rs1298268	G	T	0.06296	|| id:finn-b-I9_HYPTENS	TRUE	reported	igd	17.29503653
97	4	115969657	-0.1494	0.032	2.96599E-06	finn-b-I9_HYPTENS	rs114461628	A	T	0.02105	|| id:finn-b-I9_HYPTENS	TRUE	reported	igd	21.79722656
98	4	63370926	-0.0427	0.0103	3.34996E-05	finn-b-I9_HYPTENS	rs1000226	C	T	0.2682	|| id:finn-b-I9_HYPTENS	TRUE	reported	igd	17.18625695
99	4	3798805	-0.0663	0.0153	0.00001367	finn-b-I9_HYPTENS	rs28565380	A	G	0.1026	|| id:finn-b-I9_HYPTENS	TRUE	reported	igd	18.77777778
100	4	8734471	0.0755	0.0183	3.89996E-05	finn-b-I9_HYPTENS	rs148605125	C	T	0.07121	|| id:finn-b-I9_HYPTENS	TRUE	reported	igd	17.02126071
101	4	25057210	-0.1449	0.0342	2.24999E-05	finn-b-I9_HYPTENS	rs75177066	T	C	0.01829	|| id:finn-b-I9_HYPTENS	TRUE	reported	igd	17.95083102
102	5	65124578	0.0638	0.015	2.21401E-05	finn-b-I9_HYPTENS	rs10940053	G	T	0.1032	|| id:finn-b-I9_HYPTENS	TRUE	reported	igd	18.09084444
103	5	76302567	0.1427	0.0291	9.0099E-07	finn-b-I9_HYPTENS	rs9293700	A	G	0.02531	|| id:finn-b-I9_HYPTENS	TRUE	reported	igd	24.04705896
104	5	27970707	-0.0444	0.0105	2.32798E-05	finn-b-I9_HYPTENS	rs12187500	T	A	0.2707	|| id:finn-b-I9_HYPTENS	TRUE	reported	igd	17.88081633
105	5	32108852	-0.175	0.0426	3.99301E-05	finn-b-I9_HYPTENS	rs75449614	T	A	0.012	|| id:finn-b-I9_HYPTENS	TRUE	reported	igd	16.87550971
106	5	17270920	0.1597	0.036	9.32996E-06	finn-b-I9_HYPTENS	rs142499866	G	A	0.01654	|| id:finn-b-I9_HYPTENS	TRUE	reported	igd	19.67908179
107	5	147886157	0.0466	0.0094	6.42806E-07	finn-b-I9_HYPTENS	rs4554204	A	G	0.3863	|| id:finn-b-I9_HYPTENS	TRUE	reported	igd	24.57627886
108	5	127871750	0.0682	0.01	7.67892E-12	finn-b-I9_HYPTENS	rs6860901	T	C	0.2979	|| id:finn-b-I9_HYPTENS	TRUE	reported	igd	46.5124
109	5	96195280	0.0662	0.0124	1.006E-07	finn-b-I9_HYPTENS	rs1230366	G	A	0.8376	|| id:finn-b-I9_HYPTENS	TRUE	reported	igd	28.50182102
110	5	1263408	0.0923	0.0223	3.47096E-05	finn-b-I9_HYPTENS	rs34529095	C	T	0.04599	|| id:finn-b-I9_HYPTENS	TRUE	reported	igd	17.13143236
111	5	15891981	0.1298	0.0294	9.91608E-06	finn-b-I9_HYPTENS	rs77561631	A	G	0.02531	|| id:finn-b-I9_HYPTENS	TRUE	reported	igd	19.49192466
112	5	146426911	-0.061	0.0143	0.00001963	finn-b-I9_HYPTENS	rs77618062	A	G	0.1168	|| id:finn-b-I9_HYPTENS	TRUE	reported	igd	18.19648883
113	5	92681062	0.1383	0.0285	0.000001228	finn-b-I9_HYPTENS	rs148761812	T	C	0.0275	|| id:finn-b-I9_HYPTENS	TRUE	reported	igd	23.54803324
114	5	32832474	0.0651	0.0092	1.92398E-12	finn-b-I9_HYPTENS	rs10059884	A	C	0.5856	|| id:finn-b-I9_HYPTENS	TRUE	reported	igd	50.07100662
115	5	85765522	-0.0401	0.0091	1.09001E-05	finn-b-I9_HYPTENS	rs2962878	G	T	0.5038	|| id:finn-b-I9_HYPTENS	TRUE	reported	igd	19.41806545
116	5	114521710	0.0566	0.012	2.50398E-06	finn-b-I9_HYPTENS	rs4041	A	C	0.1733	|| id:finn-b-I9_HYPTENS	TRUE	reported	igd	22.24694444
117	5	66302605	-0.0529	0.0107	8.07198E-07	finn-b-I9_HYPTENS	rs10044452	T	C	0.2382	|| id:finn-b-I9_HYPTENS	TRUE	reported	igd	24.44239672
118	5	57438428	0.4251	0.0945	6.82606E-06	finn-b-I9_HYPTENS	rs76639689	T	C	0.002502	|| id:finn-b-I9_HYPTENS	TRUE	reported	igd	20.23571681
119	5	3714645	0.0515	0.0102	4.54999E-07	finn-b-I9_HYPTENS	rs60776549	G	A	0.2755	|| id:finn-b-I9_HYPTENS	TRUE	reported	igd	25.492599
120	5	122450771	0.0472	0.0109	0.00001502	finn-b-I9_HYPTENS	rs10077410	A	G	0.2257	|| id:finn-b-I9_HYPTENS	TRUE	reported	igd	18.75128356
121	5	157509770	0.0452	0.0109	0.00003472	finn-b-I9_HYPTENS	rs2025215	G	A	0.2254	|| id:finn-b-I9_HYPTENS	TRUE	reported	igd	17.19585893
122	5	157877453	-0.0527	0.0094	2.08598E-08	finn-b-I9_HYPTENS	rs4371736	C	G	0.3821	|| id:finn-b-I9_HYPTENS	TRUE	reported	igd	31.4315301
123	5	41890610	-0.0465	0.0104	0.000007172	finn-b-I9_HYPTENS	rs1175227	T	G	0.2634	|| id:finn-b-I9_HYPTENS	TRUE	reported	igd	19.99121672
124	5	60972519	0.1977	0.0303	7.04369E-11	finn-b-I9_HYPTENS	rs188155432	T	C	0.02363	|| id:finn-b-I9_HYPTENS	TRUE	reported	igd	42.57239486
125	5	90364101	-0.0518	0.0097	8.51491E-08	finn-b-I9_HYPTENS	rs6882081	G	A	0.6656	|| id:finn-b-I9_HYPTENS	TRUE	reported	igd	28.5178021
126	6	150984431	-0.1345	0.0172	6.23017E-15	finn-b-I9_HYPTENS	rs62434119	T	C	0.07595	|| id:finn-b-I9_HYPTENS	TRUE	reported	igd	61.14876284
127	6	153099296	-0.2289	0.0528	1.45101E-05	finn-b-I9_HYPTENS	rs137905783	C	G	0.00785	|| id:finn-b-I9_HYPTENS	TRUE	reported	igd	18.79419551
128	6	32891884	0.0738	0.015	8.67901E-07	finn-b-I9_HYPTENS	rs114463908	A	G	0.1051	|| id:finn-b-I9_HYPTENS	TRUE	reported	igd	24.2064
129	6	42071966	0.0487	0.0093	1.49001E-07	finn-b-I9_HYPTENS	rs60811587	G	A	0.4311	|| id:finn-b-I9_HYPTENS	TRUE	reported	igd	27.42155162
130	6	31587870	0.0497	0.0098	3.53997E-07	finn-b-I9_HYPTENS	rs2857694	T	A	0.3227	|| id:finn-b-I9_HYPTENS	TRUE	reported	igd	25.71938776
131	6	126218961	0.0574	0.0103	2.15402E-08	finn-b-I9_HYPTENS	rs6918791	G	C	0.728	|| id:finn-b-I9_HYPTENS	TRUE	reported	igd	31.05627298
132	6	167167501	-0.0542	0.01	6.92708E-08	finn-b-I9_HYPTENS	rs7740362	A	G	0.3002	|| id:finn-b-I9_HYPTENS	TRUE	reported	igd	29.3764
133	6	26114508	-0.0885	0.0146	1.18801E-09	finn-b-I9_HYPTENS	rs198833	A	G	0.8895	|| id:finn-b-I9_HYPTENS	TRUE	reported	igd	36.74352599
134	6	79658535	0.0476	0.0093	2.844E-07	finn-b-I9_HYPTENS	rs2063123	C	T	0.4105	|| id:finn-b-I9_HYPTENS	TRUE	reported	igd	26.19678576
135	6	137623186	0.0574	0.0123	3.14203E-06	finn-b-I9_HYPTENS	rs72981422	T	G	0.1667	|| id:finn-b-I9_HYPTENS	TRUE	reported	igd	21.77777778
136	6	143082403	-0.0932	0.0219	2.16601E-05	finn-b-I9_HYPTENS	rs137976777	A	G	0.04623	|| id:finn-b-I9_HYPTENS	TRUE	reported	igd	18.11104856
137	6	166242894	0.0549	0.0134	4.08301E-05	finn-b-I9_HYPTENS	rs903441	T	C	0.8635	|| id:finn-b-I9_HYPTENS	TRUE	reported	igd	16.7855313
138	6	12873791	0.2256	0.054	2.97701E-05	finn-b-I9_HYPTENS	rs114012120	T	C	0.007548	|| id:finn-b-I9_HYPTENS	TRUE	reported	igd	17.45382716
139	6	50646684	-0.0888	0.019	2.81903E-06	finn-b-I9_HYPTENS	rs147222641	A	G	0.06238	|| id:finn-b-I9_HYPTENS	TRUE	reported	igd	21.8433241
140	6	91649433	-0.0482	0.0114	2.35201E-05	finn-b-I9_HYPTENS	rs1875826	A	G	0.2015	|| id:finn-b-I9_HYPTENS	TRUE	reported	igd	17.87657741
141	6	127142458	0.0528	0.0091	7.13198E-09	finn-b-I9_HYPTENS	rs62426324	T	C	0.5009	|| id:finn-b-I9_HYPTENS	TRUE	reported	igd	33.66549934
142	6	52195452	0.0886	0.0212	2.95101E-05	finn-b-I9_HYPTENS	rs114171763	T	C	0.04992	|| id:finn-b-I9_HYPTENS	TRUE	reported	igd	17.46609114
143	6	118674668	0.117	0.0276	2.22998E-05	finn-b-I9_HYPTENS	rs117283965	A	G	0.02824	|| id:finn-b-I9_HYPTENS	TRUE	reported	igd	17.97022684
144	6	43352980	-0.0511	0.0099	2.39701E-07	finn-b-I9_HYPTENS	rs1214759	G	A	0.6952	|| id:finn-b-I9_HYPTENS	TRUE	reported	igd	26.6422814
145	6	95938150	0.0882	0.02	9.96896E-06	finn-b-I9_HYPTENS	rs76710640	A	T	0.0581	|| id:finn-b-I9_HYPTENS	TRUE	reported	igd	19.4481
146	6	31347538	0.0483	0.0104	3.18999E-06	finn-b-I9_HYPTENS	rs2523492	T	G	0.621	|| id:finn-b-I9_HYPTENS	TRUE	reported	igd	21.56887944
147	7	156311942	0.0405	0.0092	0.000009621	finn-b-I9_HYPTENS	rs62494194	A	G	0.4494	|| id:finn-b-I9_HYPTENS	TRUE	reported	igd	19.37913516
148	7	96155495	0.1189	0.0226	0.000000141	finn-b-I9_HYPTENS	rs117449842	G	A	0.04363	|| id:finn-b-I9_HYPTENS	TRUE	reported	igd	27.67877281
149	7	131323710	0.0908	0.0172	1.23501E-07	finn-b-I9_HYPTENS	rs75511781	G	A	0.0783	|| id:finn-b-I9_HYPTENS	TRUE	reported	igd	27.86857761
150	7	150690176	0.1721	0.0179	7.08435E-22	finn-b-I9_HYPTENS	rs3918226	T	C	0.0699	|| id:finn-b-I9_HYPTENS	TRUE	reported	igd	92.43909366
151	7	12286050	0.0439	0.0106	3.55697E-05	finn-b-I9_HYPTENS	rs10235395	G	A	0.2436	|| id:finn-b-I9_HYPTENS	TRUE	reported	igd	17.15210039
152	7	27985058	0.0896	0.0211	0.00002227	finn-b-I9_HYPTENS	rs11763181	G	T	0.9506	|| id:finn-b-I9_HYPTENS	TRUE	reported	igd	18.03229936
153	7	7332418	0.0614	0.0127	1.38899E-06	finn-b-I9_HYPTENS	rs1882599	C	G	0.8455	|| id:finn-b-I9_HYPTENS	TRUE	reported	igd	23.37379875
154	7	111571551	0.1716	0.0389	0.00001013	finn-b-I9_HYPTENS	rs78263057	G	A	0.01405	|| id:finn-b-I9_HYPTENS	TRUE	reported	igd	19.45966522
155	7	106414069	0.062	0.01	5.20703E-10	finn-b-I9_HYPTENS	rs2392929	G	T	0.2979	|| id:finn-b-I9_HYPTENS	TRUE	reported	igd	38.44
156	7	1896690	-0.0544	0.0104	1.72799E-07	finn-b-I9_HYPTENS	rs11763813	T	C	0.26	|| id:finn-b-I9_HYPTENS	TRUE	reported	igd	27.36094675
157	7	150075576	-0.0627	0.0104	1.37199E-09	finn-b-I9_HYPTENS	rs11773825	A	C	0.2659	|| id:finn-b-I9_HYPTENS	TRUE	reported	igd	36.34698595
158	7	69287445	0.0399	0.0098	4.64205E-05	finn-b-I9_HYPTENS	rs7801117	A	G	0.3203	|| id:finn-b-I9_HYPTENS	TRUE	reported	igd	16.57653061
159	7	70045931	-0.0598	0.0103	5.68303E-09	finn-b-I9_HYPTENS	rs62455829	G	A	0.2782	|| id:finn-b-I9_HYPTENS	TRUE	reported	igd	33.70760675
160	7	129663496	-0.0531	0.0097	5.07002E-08	finn-b-I9_HYPTENS	rs11556924	T	C	0.3261	|| id:finn-b-I9_HYPTENS	TRUE	reported	igd	29.9671591
161	7	899710	0.0483	0.0092	1.508E-07	finn-b-I9_HYPTENS	rs12700166	C	A	0.5539	|| id:finn-b-I9_HYPTENS	TRUE	reported	igd	27.5625
162	7	140012638	-0.0398	0.0093	1.99099E-05	finn-b-I9_HYPTENS	rs3924239	T	C	0.399	|| id:finn-b-I9_HYPTENS	TRUE	reported	igd	18.31471846
163	7	82342300	0.0904	0.022	3.96196E-05	finn-b-I9_HYPTENS	rs117958103	T	C	0.04552	|| id:finn-b-I9_HYPTENS	TRUE	reported	igd	16.8846281
164	7	27243221	0.1375	0.0157	2.01094E-18	finn-b-I9_HYPTENS	rs2023843	T	C	0.9044	|| id:finn-b-I9_HYPTENS	TRUE	reported	igd	76.7018946
165	7	150892560	0.0597	0.0124	1.32501E-06	finn-b-I9_HYPTENS	rs61733954	C	T	0.1751	|| id:finn-b-I9_HYPTENS	TRUE	reported	igd	23.17956556
166	7	151400595	0.2204	0.0471	2.86603E-06	finn-b-I9_HYPTENS	rs117647223	G	A	0.009939	|| id:finn-b-I9_HYPTENS	TRUE	reported	igd	21.89683602
167	7	114348557	-0.1558	0.0366	0.00002071	finn-b-I9_HYPTENS	rs117780887	T	A	0.0165	|| id:finn-b-I9_HYPTENS	TRUE	reported	igd	18.12060677
168	7	75686430	-0.1532	0.0321	1.86299E-06	finn-b-I9_HYPTENS	rs149523666	A	G	0.02127	|| id:finn-b-I9_HYPTENS	TRUE	reported	igd	22.77757398
169	7	27328187	0.0735	0.0133	3.38797E-08	finn-b-I9_HYPTENS	rs6961048	G	C	0.1355	|| id:finn-b-I9_HYPTENS	TRUE	reported	igd	30.5401662
170	8	51647961	0.0644	0.0121	9.8519E-08	finn-b-I9_HYPTENS	rs6992551	T	G	0.1737	|| id:finn-b-I9_HYPTENS	TRUE	reported	igd	28.32702684
171	8	102279670	0.0408	0.01	4.67305E-05	finn-b-I9_HYPTENS	rs615446	C	T	0.2996	|| id:finn-b-I9_HYPTENS	TRUE	reported	igd	16.6464
172	8	142332064	-0.0604	0.0131	4.30002E-06	finn-b-I9_HYPTENS	rs2082554	C	T	0.8605	|| id:finn-b-I9_HYPTENS	TRUE	reported	igd	21.25843482
173	8	76573711	0.0426	0.0103	3.41602E-05	finn-b-I9_HYPTENS	rs2922763	T	G	0.7297	|| id:finn-b-I9_HYPTENS	TRUE	reported	igd	17.10585352
174	8	130505863	0.2208	0.0449	8.65008E-07	finn-b-I9_HYPTENS	rs114906072	T	C	0.01066	|| id:finn-b-I9_HYPTENS	TRUE	reported	igd	24.18273719
175	8	101678943	-0.0505	0.0106	1.83202E-06	finn-b-I9_HYPTENS	rs60130955	A	G	0.2466	|| id:finn-b-I9_HYPTENS	TRUE	reported	igd	22.69713421
176	8	164050	0.0409	0.0092	9.36699E-06	finn-b-I9_HYPTENS	rs3008283	T	A	0.5694	|| id:finn-b-I9_HYPTENS	TRUE	reported	igd	19.76382325
177	8	43508916	0.148	0.0349	0.00002229	finn-b-I9_HYPTENS	rs117182261	A	G	0.01948	|| id:finn-b-I9_HYPTENS	TRUE	reported	igd	17.98343199
178	8	11838978	0.0857	0.0204	0.00002585	finn-b-I9_HYPTENS	rs28416371	G	A	0.05617	|| id:finn-b-I9_HYPTENS	TRUE	reported	igd	17.64823626
179	8	95958637	0.0464	0.0093	5.52701E-07	finn-b-I9_HYPTENS	rs1713669	G	C	0.4124	|| id:finn-b-I9_HYPTENS	TRUE	reported	igd	24.89258874
180	8	143903155	0.135	0.0318	2.25902E-05	finn-b-I9_HYPTENS	rs2572886	G	A	0.9785	|| id:finn-b-I9_HYPTENS	TRUE	reported	igd	18.02242791
181	8	143947416	0.086	0.021	4.33301E-05	finn-b-I9_HYPTENS	rs72693008	T	C	0.0501	|| id:finn-b-I9_HYPTENS	TRUE	reported	igd	16.77097506
182	8	144578727	0.1346	0.0321	2.77799E-05	finn-b-I9_HYPTENS	rs113284351	A	G	0.02117	|| id:finn-b-I9_HYPTENS	TRUE	reported	igd	17.58247688
183	8	75015097	0.3363	0.0756	8.67801E-06	finn-b-I9_HYPTENS	rs147219680	G	A	0.003892	|| id:finn-b-I9_HYPTENS	TRUE	reported	igd	19.78837554
184	8	56290516	-0.0531	0.0125	2.12799E-05	finn-b-I9_HYPTENS	rs2622554	A	G	0.1597	|| id:finn-b-I9_HYPTENS	TRUE	reported	igd	18.045504
185	8	29088056	-0.1959	0.0467	2.73899E-05	finn-b-I9_HYPTENS	rs76315288	T	C	0.01069	|| id:finn-b-I9_HYPTENS	TRUE	reported	igd	17.59685725
186	8	105966258	-0.0579	0.0126	4.36999E-06	finn-b-I9_HYPTENS	rs35783704	A	G	0.1562	|| id:finn-b-I9_HYPTENS	TRUE	reported	igd	21.11621315
187	8	14937009	-0.0648	0.0152	2.13201E-05	finn-b-I9_HYPTENS	rs62494574	T	C	0.1	|| id:finn-b-I9_HYPTENS	TRUE	reported	igd	18.17451524
188	8	136060584	-0.158	0.0371	2.03198E-05	finn-b-I9_HYPTENS	rs17579982	G	A	0.01665	|| id:finn-b-I9_HYPTENS	TRUE	reported	igd	18.13703766
189	8	25911415	-0.0528	0.0115	4.22503E-06	finn-b-I9_HYPTENS	rs11135914	C	A	0.8037	|| id:finn-b-I9_HYPTENS	TRUE	reported	igd	21.08007561
190	8	36352502	-0.1521	0.0345	1.01899E-05	finn-b-I9_HYPTENS	rs184386058	A	G	0.01819	|| id:finn-b-I9_HYPTENS	TRUE	reported	igd	19.43659735
191	8	87195838	-0.0957	0.0218	1.18599E-05	finn-b-I9_HYPTENS	rs62512386	T	C	0.04676	|| id:finn-b-I9_HYPTENS	TRUE	reported	igd	19.2712945
192	9	22096055	0.0507	0.0092	3.51803E-08	finn-b-I9_HYPTENS	rs10757274	G	A	0.432	|| id:finn-b-I9_HYPTENS	TRUE	reported	igd	30.36968336
193	9	112751571	0.0584	0.0099	3.87302E-09	finn-b-I9_HYPTENS	rs10217559	T	C	0.6936	|| id:finn-b-I9_HYPTENS	TRUE	reported	igd	34.79808183
194	9	34547536	0.0411	0.0095	1.47401E-05	finn-b-I9_HYPTENS	rs7874497	G	A	0.6255	|| id:finn-b-I9_HYPTENS	TRUE	reported	igd	18.71700831
195	9	90756684	0.255	0.0573	0.000008492	finn-b-I9_HYPTENS	rs151217323	G	A	0.006643	|| id:finn-b-I9_HYPTENS	TRUE	reported	igd	19.80482991
196	9	35906519	-0.0567	0.0105	6.80597E-08	finn-b-I9_HYPTENS	rs79284865	A	G	0.2511	|| id:finn-b-I9_HYPTENS	TRUE	reported	igd	29.16
197	9	37749508	-0.044	0.0103	1.93201E-05	finn-b-I9_HYPTENS	rs12345385	G	A	0.2706	|| id:finn-b-I9_HYPTENS	TRUE	reported	igd	18.2486568
198	9	4080864	-0.0422	0.0097	1.38599E-05	finn-b-I9_HYPTENS	rs3925025	G	T	0.3303	|| id:finn-b-I9_HYPTENS	TRUE	reported	igd	18.9269848
199	9	72024947	-0.3013	0.0698	1.58701E-05	finn-b-I9_HYPTENS	rs142888208	T	C	0.004427	|| id:finn-b-I9_HYPTENS	TRUE	reported	igd	18.63319882
200	9	75541227	0.0975	0.0225	1.44301E-05	finn-b-I9_HYPTENS	rs34878833	A	G	0.04339	|| id:finn-b-I9_HYPTENS	TRUE	reported	igd	18.77777778
201	9	6595511	0.2306	0.0477	0.000001359	finn-b-I9_HYPTENS	rs11999044	T	C	0.009431	|| id:finn-b-I9_HYPTENS	TRUE	reported	igd	23.37124498
202	9	5873024	0.0639	0.0156	4.37301E-05	finn-b-I9_HYPTENS	rs10815298	G	C	0.9059	|| id:finn-b-I9_HYPTENS	TRUE	reported	igd	16.77847633
203	9	36854809	-0.0933	0.0225	3.46298E-05	finn-b-I9_HYPTENS	rs72735603	G	A	0.044	|| id:finn-b-I9_HYPTENS	TRUE	reported	igd	17.19484444
204	9	23920300	0.1484	0.0314	2.35402E-06	finn-b-I9_HYPTENS	rs141336538	A	C	0.02225	|| id:finn-b-I9_HYPTENS	TRUE	reported	igd	22.33615968
205	9	136454663	-0.1222	0.0187	6.31539E-11	finn-b-I9_HYPTENS	rs72779268	A	C	0.06436	|| id:finn-b-I9_HYPTENS	TRUE	reported	igd	42.70307987
206	9	107720120	-0.0485	0.0107	5.69102E-06	finn-b-I9_HYPTENS	rs2487028	C	T	0.2397	|| id:finn-b-I9_HYPTENS	TRUE	reported	igd	20.54546249
207	9	78185004	0.1084	0.0264	4.01301E-05	finn-b-I9_HYPTENS	rs11998975	G	C	0.03145	|| id:finn-b-I9_HYPTENS	TRUE	reported	igd	16.8597337
208	9	131938913	-0.0549	0.0107	2.98099E-07	finn-b-I9_HYPTENS	rs1075650	C	T	0.2443	|| id:finn-b-I9_HYPTENS	TRUE	reported	igd	26.32553061
209	9	136535677	-0.0855	0.0177	1.28801E-06	finn-b-I9_HYPTENS	rs10993958	A	G	0.07394	|| id:finn-b-I9_HYPTENS	TRUE	reported	igd	23.33381212
210	9	129232031	0.3255	0.0748	0.00001341	finn-b-I9_HYPTENS	rs117457046	A	G	0.003829	|| id:finn-b-I9_HYPTENS	TRUE	reported	igd	18.93645979
211	10	105776033	0.076	0.0176	1.50501E-05	finn-b-I9_HYPTENS	rs12250162	C	T	0.07291	|| id:finn-b-I9_HYPTENS	TRUE	reported	igd	18.64669421
212	10	107409168	-0.0432	0.0101	0.000021	finn-b-I9_HYPTENS	rs12244751	C	T	0.2807	|| id:finn-b-I9_HYPTENS	TRUE	reported	igd	18.29467699
213	10	63515681	-0.0818	0.0151	5.82197E-08	finn-b-I9_HYPTENS	rs72831343	G	T	0.102	|| id:finn-b-I9_HYPTENS	TRUE	reported	igd	29.34625674
214	10	72491666	-0.104	0.0247	2.47503E-05	finn-b-I9_HYPTENS	rs78701156	A	G	0.03585	|| id:finn-b-I9_HYPTENS	TRUE	reported	igd	17.72853186
215	10	104849116	-0.1089	0.0158	5.48656E-12	finn-b-I9_HYPTENS	rs10786736	C	G	0.09173	|| id:finn-b-I9_HYPTENS	TRUE	reported	igd	47.50524756
216	10	132736217	0.101	0.0236	1.89701E-05	finn-b-I9_HYPTENS	rs184232358	A	G	0.03776	|| id:finn-b-I9_HYPTENS	TRUE	reported	igd	18.31549842
217	10	126733546	0.0393	0.0095	3.47096E-05	finn-b-I9_HYPTENS	rs11245482	C	T	0.364	|| id:finn-b-I9_HYPTENS	TRUE	reported	igd	17.1134626
218	10	18726054	-0.0539	0.0108	6.09102E-07	finn-b-I9_HYPTENS	rs7070847	A	G	0.2346	|| id:finn-b-I9_HYPTENS	TRUE	reported	igd	24.90749314
219	10	45727796	0.0786	0.0193	4.51097E-05	finn-b-I9_HYPTENS	rs10751381	A	G	0.9385	|| id:finn-b-I9_HYPTENS	TRUE	reported	igd	16.58557277
220	10	2147839	-0.1384	0.0322	0.00001761	finn-b-I9_HYPTENS	rs117776439	A	T	0.02074	|| id:finn-b-I9_HYPTENS	TRUE	reported	igd	18.47397863
221	10	123748802	-0.0457	0.0094	1.17401E-06	finn-b-I9_HYPTENS	rs7900585	A	G	0.3965	|| id:finn-b-I9_HYPTENS	TRUE	reported	igd	23.63614758
222	10	89990174	-0.0592	0.0136	0.00001431	finn-b-I9_HYPTENS	rs7922490	T	C	0.1308	|| id:finn-b-I9_HYPTENS	TRUE	reported	igd	18.94809689
223	10	57066496	-0.0954	0.0229	0.00003185	finn-b-I9_HYPTENS	rs72803844	C	T	0.04362	|| id:finn-b-I9_HYPTENS	TRUE	reported	igd	17.35504662
224	10	95903455	-0.0482	0.0101	1.88899E-06	finn-b-I9_HYPTENS	rs1223634	T	C	0.2848	|| id:finn-b-I9_HYPTENS	TRUE	reported	igd	22.77462994
225	10	96039597	-0.0751	0.0096	5.64287E-15	finn-b-I9_HYPTENS	rs2274224	C	G	0.3456	|| id:finn-b-I9_HYPTENS	TRUE	reported	igd	61.19802517
226	10	115781547	0.0799	0.0096	8.71365E-17	finn-b-I9_HYPTENS	rs2782981	C	T	0.6546	|| id:finn-b-I9_HYPTENS	TRUE	reported	igd	69.27094184
227	10	18440444	0.06	0.0094	1.45399E-10	finn-b-I9_HYPTENS	rs1888693	A	G	0.386	|| id:finn-b-I9_HYPTENS	TRUE	reported	igd	40.74241738
228	10	22839952	0.1494	0.033	5.99294E-06	finn-b-I9_HYPTENS	rs45469096	G	A	0.01971	|| id:finn-b-I9_HYPTENS	TRUE	reported	igd	20.49619835
229	10	23711654	-0.0385	0.0093	3.25499E-05	finn-b-I9_HYPTENS	rs76223480	T	C	0.4768	|| id:finn-b-I9_HYPTENS	TRUE	reported	igd	17.1378194
230	10	88748296	-0.0691	0.0148	3.27597E-06	finn-b-I9_HYPTENS	rs146181077	G	A	0.1074	|| id:finn-b-I9_HYPTENS	TRUE	reported	igd	21.79880387
231	10	76784150	-0.2064	0.0454	0.000005426	finn-b-I9_HYPTENS	rs72803452	T	C	0.01044	|| id:finn-b-I9_HYPTENS	TRUE	reported	igd	20.66843913
232	10	16927027	0.0472	0.0108	1.20199E-05	finn-b-I9_HYPTENS	rs809698	T	C	0.7675	|| id:finn-b-I9_HYPTENS	TRUE	reported	igd	19.10013717
233	10	65823446	0.0663	0.0132	5.54702E-07	finn-b-I9_HYPTENS	rs7922671	T	C	0.1362	|| id:finn-b-I9_HYPTENS	TRUE	reported	igd	25.22778926
234	10	130325915	-0.1186	0.0285	3.16497E-05	finn-b-I9_HYPTENS	rs182734648	A	T	0.0279	|| id:finn-b-I9_HYPTENS	TRUE	reported	igd	17.31727916
235	10	121839738	-0.0503	0.0107	0.000002319	finn-b-I9_HYPTENS	rs11593681	A	G	0.2416	|| id:finn-b-I9_HYPTENS	TRUE	reported	igd	22.09878592
236	11	46364386	-0.2185	0.0506	0.00001575	finn-b-I9_HYPTENS	rs145318637	G	A	0.008714	|| id:finn-b-I9_HYPTENS	TRUE	reported	igd	18.64669421
237	11	65566719	-0.0613	0.0093	5.41751E-11	finn-b-I9_HYPTENS	rs557675	G	T	0.3969	|| id:finn-b-I9_HYPTENS	TRUE	reported	igd	43.44652561
238	11	10155174	0.0682	0.0114	2.474E-09	finn-b-I9_HYPTENS	rs7123467	G	C	0.202	|| id:finn-b-I9_HYPTENS	TRUE	reported	igd	35.78978147
239	11	21620165	0.1313	0.032	4.18504E-05	finn-b-I9_HYPTENS	rs185811838	T	G	0.0211	|| id:finn-b-I9_HYPTENS	TRUE	reported	igd	16.83563477
240	11	105926628	-0.0396	0.0094	0.00002678	finn-b-I9_HYPTENS	rs11226908	A	G	0.39	|| id:finn-b-I9_HYPTENS	TRUE	reported	igd	17.74739701
241	11	127638978	0.2039	0.0491	3.27198E-05	finn-b-I9_HYPTENS	rs142278936	C	G	0.009384	|| id:finn-b-I9_HYPTENS	TRUE	reported	igd	17.24532833
242	11	29845001	0.0868	0.0212	4.29804E-05	finn-b-I9_HYPTENS	rs138955538	A	G	0.04873	|| id:finn-b-I9_HYPTENS	TRUE	reported	igd	16.76361695
243	11	76501557	-0.0562	0.012	0.000002689	finn-b-I9_HYPTENS	rs1837679	G	A	0.1793	|| id:finn-b-I9_HYPTENS	TRUE	reported	igd	21.93361111
244	11	4686295	-0.0522	0.0104	4.92198E-07	finn-b-I9_HYPTENS	rs6578514	C	T	0.7379	|| id:finn-b-I9_HYPTENS	TRUE	reported	igd	25.19267751
245	11	1920255	0.0759	0.0109	3.12176E-12	finn-b-I9_HYPTENS	rs7483477	G	T	0.2348	|| id:finn-b-I9_HYPTENS	TRUE	reported	igd	48.48758522
246	11	32900675	0.1143	0.0249	4.63202E-06	finn-b-I9_HYPTENS	rs72901223	A	G	0.03558	|| id:finn-b-I9_HYPTENS	TRUE	reported	igd	21.0714182
247	11	61270938	-0.1	0.0238	0.00002561	finn-b-I9_HYPTENS	rs56788999	C	T	0.03904	|| id:finn-b-I9_HYPTENS	TRUE	reported	igd	17.65412047
248	11	120153414	-0.0446	0.0106	2.60298E-05	finn-b-I9_HYPTENS	rs11217787	A	T	0.2486	|| id:finn-b-I9_HYPTENS	TRUE	reported	igd	17.70345319
249	11	116515557	0.0546	0.0124	1.13501E-05	finn-b-I9_HYPTENS	rs71480306	C	T	0.1622	|| id:finn-b-I9_HYPTENS	TRUE	reported	igd	19.3883975
250	11	22516207	0.0639	0.0148	1.56498E-05	finn-b-I9_HYPTENS	rs11026587	C	A	0.1077	|| id:finn-b-I9_HYPTENS	TRUE	reported	igd	18.6413897
251	11	43628749	-0.0394	0.0094	3.05598E-05	finn-b-I9_HYPTENS	rs4755720	T	C	0.6256	|| id:finn-b-I9_HYPTENS	TRUE	reported	igd	17.56858307
252	11	45559883	0.1237	0.0233	1.09099E-07	finn-b-I9_HYPTENS	rs112968778	T	G	0.04129	|| id:finn-b-I9_HYPTENS	TRUE	reported	igd	28.18561771
253	11	106446645	0.0896	0.0212	2.44399E-05	finn-b-I9_HYPTENS	rs61903121	C	T	0.05094	|| id:finn-b-I9_HYPTENS	TRUE	reported	igd	17.86258455
254	11	26274622	0.044	0.0094	2.68999E-06	finn-b-I9_HYPTENS	rs12806338	G	A	0.3859	|| id:finn-b-I9_HYPTENS	TRUE	reported	igd	21.91036668
255	11	116711141	-0.0453	0.0092	9.06108E-07	finn-b-I9_HYPTENS	rs2727784	T	C	0.5593	|| id:finn-b-I9_HYPTENS	TRUE	reported	igd	24.24491966
256	11	100610546	0.0632	0.0104	1.358E-09	finn-b-I9_HYPTENS	rs604723	C	T	0.741	|| id:finn-b-I9_HYPTENS	TRUE	reported	igd	36.92899408
257	11	57183218	0.1356	0.0264	2.83302E-07	finn-b-I9_HYPTENS	rs72923979	G	C	0.03293	|| id:finn-b-I9_HYPTENS	TRUE	reported	igd	26.3822314
258	12	50266164	0.0529	0.0103	2.52302E-07	finn-b-I9_HYPTENS	rs706798	A	G	0.7252	|| id:finn-b-I9_HYPTENS	TRUE	reported	igd	26.37769818
259	12	64779458	0.0525	0.0108	1.13999E-06	finn-b-I9_HYPTENS	rs7485037	C	T	0.2341	|| id:finn-b-I9_HYPTENS	TRUE	reported	igd	23.63040123
260	12	124572307	-0.1628	0.0339	1.60901E-06	finn-b-I9_HYPTENS	rs76484775	T	C	0.01852	|| id:finn-b-I9_HYPTENS	TRUE	reported	igd	23.06266044
261	12	73032573	-0.1219	0.0301	4.97302E-05	finn-b-I9_HYPTENS	rs9705508	C	G	0.02727	|| id:finn-b-I9_HYPTENS	TRUE	reported	igd	16.40115451
262	12	15507223	0.0435	0.0103	2.21498E-05	finn-b-I9_HYPTENS	rs11831208	A	G	0.2706	|| id:finn-b-I9_HYPTENS	TRUE	reported	igd	17.83627109
263	12	20351276	-0.0515	0.0111	3.42799E-06	finn-b-I9_HYPTENS	rs73073676	T	A	0.2182	|| id:finn-b-I9_HYPTENS	TRUE	reported	igd	21.52625599
264	12	21555672	0.0549	0.012	4.69797E-06	finn-b-I9_HYPTENS	rs77369115	T	A	0.1797	|| id:finn-b-I9_HYPTENS	TRUE	reported	igd	20.930625
265	12	50673484	-0.0421	0.0093	5.55495E-06	finn-b-I9_HYPTENS	rs7967954	A	G	0.5893	|| id:finn-b-I9_HYPTENS	TRUE	reported	igd	20.49265811
266	12	24678461	-0.0608	0.0125	1.21899E-06	finn-b-I9_HYPTENS	rs55741061	A	G	0.1599	|| id:finn-b-I9_HYPTENS	TRUE	reported	igd	23.658496
267	12	100740801	-0.0592	0.0141	2.80698E-05	finn-b-I9_HYPTENS	rs7981002	C	T	0.1181	|| id:finn-b-I9_HYPTENS	TRUE	reported	igd	17.62808712
268	12	79656114	0.0716	0.0175	4.32504E-05	finn-b-I9_HYPTENS	rs80203904	A	C	0.07306	|| id:finn-b-I9_HYPTENS	TRUE	reported	igd	16.73978776
269	12	12883570	-0.0606	0.0092	5.00956E-11	finn-b-I9_HYPTENS	rs12828438	G	A	0.5354	|| id:finn-b-I9_HYPTENS	TRUE	reported	igd	43.38799622
270	12	53566790	0.1715	0.0321	9.45192E-08	finn-b-I9_HYPTENS	rs7139122	A	G	0.02074	|| id:finn-b-I9_HYPTENS	TRUE	reported	igd	28.54422026
271	12	54441498	-0.0748	0.0094	2.13304E-15	finn-b-I9_HYPTENS	rs7134677	T	C	0.3819	|| id:finn-b-I9_HYPTENS	TRUE	reported	igd	63.32095971
272	12	90110782	0.0663	0.0106	4.58997E-10	finn-b-I9_HYPTENS	rs1980235	A	G	0.7541	|| id:finn-b-I9_HYPTENS	TRUE	reported	igd	39.12148451
273	12	118123929	0.0592	0.0143	3.31597E-05	finn-b-I9_HYPTENS	rs11068627	G	A	0.1197	|| id:finn-b-I9_HYPTENS	TRUE	reported	igd	17.13844198
274	12	115556307	-0.0592	0.0096	5.91398E-10	finn-b-I9_HYPTENS	rs35427	G	T	0.3644	|| id:finn-b-I9_HYPTENS	TRUE	reported	igd	38.02777778
275	12	25471228	0.0407	0.0093	1.17101E-05	finn-b-I9_HYPTENS	rs11047952	A	T	0.4138	|| id:finn-b-I9_HYPTENS	TRUE	reported	igd	19.15238756
276	12	111865049	-0.0816	0.0093	1.36616E-18	finn-b-I9_HYPTENS	rs7310615	G	C	0.5863	|| id:finn-b-I9_HYPTENS	TRUE	reported	igd	76.98647242
277	13	91084929	0.0926	0.0223	3.35699E-05	finn-b-I9_HYPTENS	rs144374353	T	C	0.04507	|| id:finn-b-I9_HYPTENS	TRUE	reported	igd	17.24297693
278	13	72365326	0.0849	0.0153	2.70402E-08	finn-b-I9_HYPTENS	rs303949	C	A	0.09934	|| id:finn-b-I9_HYPTENS	TRUE	reported	igd	30.79161861
279	13	97989911	-0.0525	0.0108	0.000001213	finn-b-I9_HYPTENS	rs9556704	G	A	0.2344	|| id:finn-b-I9_HYPTENS	TRUE	reported	igd	23.63040123
280	13	110699373	-0.0851	0.0168	4.07202E-07	finn-b-I9_HYPTENS	rs55761308	C	T	0.08298	|| id:finn-b-I9_HYPTENS	TRUE	reported	igd	25.65904904
281	13	22318506	0.0595	0.0093	1.358E-10	finn-b-I9_HYPTENS	rs629042	C	G	0.5795	|| id:finn-b-I9_HYPTENS	TRUE	reported	igd	40.93247774
282	13	30119500	-0.0494	0.0095	2.12598E-07	finn-b-I9_HYPTENS	rs9508490	T	C	0.6423	|| id:finn-b-I9_HYPTENS	TRUE	reported	igd	27.04
283	13	49556814	-0.4071	0.0787	2.27499E-07	finn-b-I9_HYPTENS	rs75448527	A	C	0.003445	|| id:finn-b-I9_HYPTENS	TRUE	reported	igd	26.75794397
284	13	110847217	0.0475	0.0093	3.34003E-07	finn-b-I9_HYPTENS	rs9521643	G	A	0.4175	|| id:finn-b-I9_HYPTENS	TRUE	reported	igd	26.08683085
285	14	26954505	0.1804	0.0371	0.000001128	finn-b-I9_HYPTENS	rs17582007	G	A	0.01571	|| id:finn-b-I9_HYPTENS	TRUE	reported	igd	23.64423391
286	14	24810401	0.0497	0.011	6.25706E-06	finn-b-I9_HYPTENS	rs3212242	T	C	0.2199	|| id:finn-b-I9_HYPTENS	TRUE	reported	igd	20.41396694
287	14	94987996	0.8572	0.1966	0.00001297	finn-b-I9_HYPTENS	rs8004587	T	G	0.0005638	|| id:finn-b-I9_HYPTENS	TRUE	reported	igd	19.01066451
288	14	68805995	-0.0922	0.0213	0.00001516	finn-b-I9_HYPTENS	rs140355828	T	G	0.04919	|| id:finn-b-I9_HYPTENS	TRUE	reported	igd	18.73711124
289	14	24483904	0.232	0.0565	0.00003992	finn-b-I9_HYPTENS	rs142554588	C	G	0.007337	|| id:finn-b-I9_HYPTENS	TRUE	reported	igd	16.86083483
290	14	61492969	-0.1187	0.0286	3.27198E-05	finn-b-I9_HYPTENS	rs143574562	A	T	0.02659	|| id:finn-b-I9_HYPTENS	TRUE	reported	igd	17.22540222
291	14	69293088	0.1124	0.023	9.94397E-07	finn-b-I9_HYPTENS	rs140503338	G	T	0.0412	|| id:finn-b-I9_HYPTENS	TRUE	reported	igd	23.88234405
292	14	65320013	-0.1374	0.0269	3.19198E-07	finn-b-I9_HYPTENS	rs112074516	G	A	0.03018	|| id:finn-b-I9_HYPTENS	TRUE	reported	igd	26.0896892
293	14	94192137	0.0419	0.0095	0.00001096	finn-b-I9_HYPTENS	rs10873436	T	C	0.6297	|| id:finn-b-I9_HYPTENS	TRUE	reported	igd	19.45274238
294	15	38502243	-0.0487	0.0116	2.77402E-05	finn-b-I9_HYPTENS	rs11854769	T	C	0.1928	|| id:finn-b-I9_HYPTENS	TRUE	reported	igd	17.62552021
295	15	80498420	0.0419	0.0096	1.37899E-05	finn-b-I9_HYPTENS	rs61568648	C	T	0.3362	|| id:finn-b-I9_HYPTENS	TRUE	reported	igd	19.04958767
296	15	40404351	-0.1463	0.0333	0.00001109	finn-b-I9_HYPTENS	rs72731428	A	G	0.0204	|| id:finn-b-I9_HYPTENS	TRUE	reported	igd	19.30190551
297	15	38115684	-0.1981	0.0479	3.56098E-05	finn-b-I9_HYPTENS	rs16965683	G	A	0.009411	|| id:finn-b-I9_HYPTENS	TRUE	reported	igd	17.10400931
298	15	25312254	-0.1473	0.0346	0.00002072	finn-b-I9_HYPTENS	rs62001998	T	G	0.01802	|| id:finn-b-I9_HYPTENS	TRUE	reported	igd	18.12396839
299	15	64279834	0.0393	0.0095	3.18302E-05	finn-b-I9_HYPTENS	rs8040233	T	C	0.6367	|| id:finn-b-I9_HYPTENS	TRUE	reported	igd	17.1134626
300	15	99168319	-0.5619	0.1181	1.95799E-06	finn-b-I9_HYPTENS	rs138798453	T	C	0.001588	|| id:finn-b-I9_HYPTENS	TRUE	reported	igd	22.63696863
301	15	61036561	0.6328	0.1525	3.34603E-05	finn-b-I9_HYPTENS	rs150265413	C	T	0.0009671	|| id:finn-b-I9_HYPTENS	TRUE	reported	igd	17.21841827
302	15	31617788	0.0572	0.0115	6.72496E-07	finn-b-I9_HYPTENS	rs6493604	C	A	0.8036	|| id:finn-b-I9_HYPTENS	TRUE	reported	igd	24.73981096
303	15	75114322	-0.055	0.0094	4.20901E-09	finn-b-I9_HYPTENS	rs11636952	C	T	0.6071	|| id:finn-b-I9_HYPTENS	TRUE	reported	igd	34.23494794
304	15	81010249	0.054	0.0093	7.32993E-09	finn-b-I9_HYPTENS	rs2062316	G	A	0.4006	|| id:finn-b-I9_HYPTENS	TRUE	reported	igd	33.71488033
305	15	91428955	0.0834	0.0103	4.96935E-16	finn-b-I9_HYPTENS	rs1894400	T	C	0.2713	|| id:finn-b-I9_HYPTENS	TRUE	reported	igd	65.56282402
306	16	4378272	0.062	0.013	1.66299E-06	finn-b-I9_HYPTENS	rs3888628	A	T	0.1486	|| id:finn-b-I9_HYPTENS	TRUE	reported	igd	22.74556213
307	16	7667965	-0.0538	0.0121	8.55303E-06	finn-b-I9_HYPTENS	rs4787049	C	A	0.8281	|| id:finn-b-I9_HYPTENS	TRUE	reported	igd	19.76941466
308	16	75368259	0.0386	0.0094	3.94303E-05	finn-b-I9_HYPTENS	rs10871309	T	C	0.3846	|| id:finn-b-I9_HYPTENS	TRUE	reported	igd	16.86238117
309	16	3573921	0.0382	0.0092	3.26002E-05	finn-b-I9_HYPTENS	rs2890116	A	G	0.436	|| id:finn-b-I9_HYPTENS	TRUE	reported	igd	17.2405482
310	16	51664579	-0.1191	0.028	2.12002E-05	finn-b-I9_HYPTENS	rs111956834	C	T	0.02771	|| id:finn-b-I9_HYPTENS	TRUE	reported	igd	18.0928699
311	16	19146108	0.0465	0.0101	4.11803E-06	finn-b-I9_HYPTENS	rs11074364	T	G	0.2914	|| id:finn-b-I9_HYPTENS	TRUE	reported	igd	21.19645133
312	16	20392332	-0.0578	0.0111	1.67699E-07	finn-b-I9_HYPTENS	rs77924615	A	G	0.2222	|| id:finn-b-I9_HYPTENS	TRUE	reported	igd	27.1150069
313	16	89857008	0.0518	0.0102	3.40596E-07	finn-b-I9_HYPTENS	rs11640316	G	A	0.7057	|| id:finn-b-I9_HYPTENS	TRUE	reported	igd	25.79046521
314	16	53826028	0.0617	0.0093	2.76376E-11	finn-b-I9_HYPTENS	rs28567725	C	T	0.4136	|| id:finn-b-I9_HYPTENS	TRUE	reported	igd	44.0153775
315	16	62936653	0.0516	0.0115	7.07001E-06	finn-b-I9_HYPTENS	rs2914486	C	T	0.1969	|| id:finn-b-I9_HYPTENS	TRUE	reported	igd	20.13277883
316	16	86733566	-0.13	0.0292	8.57907E-06	finn-b-I9_HYPTENS	rs67493690	A	C	0.02565	|| id:finn-b-I9_HYPTENS	TRUE	reported	igd	19.82079189
317	16	4224292	0.1139	0.0222	2.97201E-07	finn-b-I9_HYPTENS	rs149441293	C	G	0.04471	|| id:finn-b-I9_HYPTENS	TRUE	reported	igd	26.32337067
318	16	81607756	0.0501	0.0093	7.00293E-08	finn-b-I9_HYPTENS	rs12924701	G	A	0.5931	|| id:finn-b-I9_HYPTENS	TRUE	reported	igd	29.02081165
319	16	1881180	0.0841	0.0197	1.99802E-05	finn-b-I9_HYPTENS	rs62038396	A	C	0.05681	|| id:finn-b-I9_HYPTENS	TRUE	reported	igd	18.22466438
320	17	7109995	0.0519	0.0096	6.11899E-08	finn-b-I9_HYPTENS	rs390200	G	A	0.6517	|| id:finn-b-I9_HYPTENS	TRUE	reported	igd	29.22753906
321	17	56404934	-0.1088	0.019	1.10499E-08	finn-b-I9_HYPTENS	rs117515309	A	C	0.06182	|| id:finn-b-I9_HYPTENS	TRUE	reported	igd	32.79069252
322	17	58790106	-0.1306	0.025	1.80302E-07	finn-b-I9_HYPTENS	rs79538876	C	T	0.03508	|| id:finn-b-I9_HYPTENS	TRUE	reported	igd	27.290176
323	17	47357173	-0.1364	0.0215	2.10902E-10	finn-b-I9_HYPTENS	rs79873333	T	C	0.04758	|| id:finn-b-I9_HYPTENS	TRUE	reported	igd	40.24869659
324	17	12265784	-0.1341	0.0308	0.00001344	finn-b-I9_HYPTENS	rs116990006	A	G	0.0228	|| id:finn-b-I9_HYPTENS	TRUE	reported	igd	18.95641128
325	17	45138033	0.0707	0.0115	7.54397E-10	finn-b-I9_HYPTENS	rs145153053	G	A	0.1963	|| id:finn-b-I9_HYPTENS	TRUE	reported	igd	37.7957656
326	17	4315506	-0.0404	0.0098	0.00003395	finn-b-I9_HYPTENS	rs11078506	T	C	0.3221	|| id:finn-b-I9_HYPTENS	TRUE	reported	igd	16.99458559
327	17	33840760	-0.0524	0.0115	5.05103E-06	finn-b-I9_HYPTENS	rs34254270	A	G	0.1954	|| id:finn-b-I9_HYPTENS	TRUE	reported	igd	20.76189036
328	17	79373097	0.2349	0.0506	3.46402E-06	finn-b-I9_HYPTENS	rs571448220	A	C	0.008803	|| id:finn-b-I9_HYPTENS	TRUE	reported	igd	21.55087956
329	17	59482169	0.0614	0.0102	1.65299E-09	finn-b-I9_HYPTENS	rs35619711	G	C	0.7217	|| id:finn-b-I9_HYPTENS	TRUE	reported	igd	36.23567859
330	17	43195981	0.0454	0.01	5.90595E-06	finn-b-I9_HYPTENS	rs3744760	C	T	0.2938	|| id:finn-b-I9_HYPTENS	TRUE	reported	igd	20.6116
331	17	61545486	-0.1068	0.0166	1.409E-10	finn-b-I9_HYPTENS	rs9899012	A	G	0.08387	|| id:finn-b-I9_HYPTENS	TRUE	reported	igd	41.39294528
332	18	23202869	0.2004	0.0437	4.61498E-06	finn-b-I9_HYPTENS	rs142673248	A	G	0.0111	|| id:finn-b-I9_HYPTENS	TRUE	reported	igd	21.02967497
333	18	42117774	0.0438	0.0103	2.08598E-05	finn-b-I9_HYPTENS	rs2063502	G	T	0.7309	|| id:finn-b-I9_HYPTENS	TRUE	reported	igd	18.08313696
334	18	54658566	0.0419	0.01	2.90603E-05	finn-b-I9_HYPTENS	rs62098628	G	A	0.2941	|| id:finn-b-I9_HYPTENS	TRUE	reported	igd	17.5561
335	18	57915448	0.0922	0.0179	2.43198E-07	finn-b-I9_HYPTENS	rs61194872	T	C	0.07146	|| id:finn-b-I9_HYPTENS	TRUE	reported	igd	26.53113199
336	18	24626889	0.0387	0.0092	2.67202E-05	finn-b-I9_HYPTENS	rs1485803	A	G	0.4662	|| id:finn-b-I9_HYPTENS	TRUE	reported	igd	17.69482514
337	18	48886201	-0.1616	0.0338	1.80901E-06	finn-b-I9_HYPTENS	rs117003065	G	A	0.0188	|| id:finn-b-I9_HYPTENS	TRUE	reported	igd	22.85858338
338	18	38766660	0.064	0.0149	1.81602E-05	finn-b-I9_HYPTENS	rs117162894	C	T	0.1051	|| id:finn-b-I9_HYPTENS	TRUE	reported	igd	18.44961939
339	18	70121207	-0.0419	0.0097	1.62002E-05	finn-b-I9_HYPTENS	rs9963829	A	G	0.3256	|| id:finn-b-I9_HYPTENS	TRUE	reported	igd	18.65883728
340	19	13049156	-0.1348	0.0266	3.92998E-07	finn-b-I9_HYPTENS	rs117651954	T	G	0.0317	|| id:finn-b-I9_HYPTENS	TRUE	reported	igd	25.68127085
341	19	40419243	0.0879	0.018	1.10301E-06	finn-b-I9_HYPTENS	rs147500579	A	G	0.06898	|| id:finn-b-I9_HYPTENS	TRUE	reported	igd	23.84694444
342	19	7428651	0.0777	0.0156	6.37206E-07	finn-b-I9_HYPTENS	rs117209069	A	G	0.09657	|| id:finn-b-I9_HYPTENS	TRUE	reported	igd	24.80806213
343	19	41433151	0.1525	0.0375	4.72302E-05	finn-b-I9_HYPTENS	rs149613261	A	G	0.01501	|| id:finn-b-I9_HYPTENS	TRUE	reported	igd	16.53777778
344	19	49632230	0.1862	0.0437	2.00798E-05	finn-b-I9_HYPTENS	rs35298280	G	A	0.0111	|| id:finn-b-I9_HYPTENS	TRUE	reported	igd	18.15500945
345	19	11526765	-0.0833	0.0093	2.66011E-19	finn-b-I9_HYPTENS	rs167479	T	G	0.4249	|| id:finn-b-I9_HYPTENS	TRUE	reported	igd	80.22765638
346	19	50935809	-0.1411	0.0314	6.96402E-06	finn-b-I9_HYPTENS	rs138877676	T	G	0.02214	|| id:finn-b-I9_HYPTENS	TRUE	reported	igd	20.19271573
347	19	2613136	0.0998	0.0215	3.34403E-06	finn-b-I9_HYPTENS	rs62123724	G	A	0.04749	|| id:finn-b-I9_HYPTENS	TRUE	reported	igd	21.54686858
348	19	54382473	-0.0947	0.0187	4.07202E-07	finn-b-I9_HYPTENS	rs113270767	T	G	0.06476	|| id:finn-b-I9_HYPTENS	TRUE	reported	igd	25.64582916
349	19	1866427	-0.0475	0.0114	3.28398E-05	finn-b-I9_HYPTENS	rs10411032	C	T	0.2002	|| id:finn-b-I9_HYPTENS	TRUE	reported	igd	17.36111111
350	19	12018867	-0.2554	0.0436	4.689E-09	finn-b-I9_HYPTENS	rs189498068	G	A	0.01165	|| id:finn-b-I9_HYPTENS	TRUE	reported	igd	34.31379934
351	20	57170004	-0.0579	0.0117	7.59399E-07	finn-b-I9_HYPTENS	rs55819839	T	C	0.1898	|| id:finn-b-I9_HYPTENS	TRUE	reported	igd	24.48980934
352	20	57735448	0.1406	0.0122	1.02589E-30	finn-b-I9_HYPTENS	rs78302204	A	G	0.1674	|| id:finn-b-I9_HYPTENS	TRUE	reported	igd	132.8161784
353	20	57877621	0.1233	0.0256	1.42099E-06	finn-b-I9_HYPTENS	rs9679855	G	A	0.03347	|| id:finn-b-I9_HYPTENS	TRUE	reported	igd	23.19776917
354	20	24524692	0.0712	0.0172	3.47896E-05	finn-b-I9_HYPTENS	rs6049772	T	C	0.07531	|| id:finn-b-I9_HYPTENS	TRUE	reported	igd	17.13574905
355	20	31225355	0.0566	0.0112	4.87798E-07	finn-b-I9_HYPTENS	rs12624715	G	A	0.2088	|| id:finn-b-I9_HYPTENS	TRUE	reported	igd	25.53858418
356	20	46390748	0.0444	0.0105	2.29002E-05	finn-b-I9_HYPTENS	rs1806727	C	T	0.2598	|| id:finn-b-I9_HYPTENS	TRUE	reported	igd	17.88081633
357	20	47048545	-0.1686	0.0397	2.19402E-05	finn-b-I9_HYPTENS	rs111574802	C	T	0.01447	|| id:finn-b-I9_HYPTENS	TRUE	reported	igd	18.03574669
358	20	39942516	-0.0485	0.0101	1.56099E-06	finn-b-I9_HYPTENS	rs142391093	G	A	0.289	|| id:finn-b-I9_HYPTENS	TRUE	reported	igd	23.05901382
359	20	24293262	0.1769	0.0381	3.42302E-06	finn-b-I9_HYPTENS	rs6083485	A	G	0.01432	|| id:finn-b-I9_HYPTENS	TRUE	reported	igd	21.55786334
360	20	62714025	0.0544	0.0114	1.92899E-06	finn-b-I9_HYPTENS	rs6122247	C	T	0.2043	|| id:finn-b-I9_HYPTENS	TRUE	reported	igd	22.77131425
361	20	5854605	0.1065	0.0246	1.52398E-05	finn-b-I9_HYPTENS	rs73598717	A	G	0.03569	|| id:finn-b-I9_HYPTENS	TRUE	reported	igd	18.74256395
362	20	10936029	0.0563	0.0105	8.019E-08	finn-b-I9_HYPTENS	rs1555959	G	T	0.2552	|| id:finn-b-I9_HYPTENS	TRUE	reported	igd	28.75002268
363	20	8617819	-0.0471	0.0115	4.13704E-05	finn-b-I9_HYPTENS	rs6039214	A	G	0.1964	|| id:finn-b-I9_HYPTENS	TRUE	reported	igd	16.77436673
364	21	44793464	0.0488	0.0094	1.94102E-07	finn-b-I9_HYPTENS	rs672929	G	A	0.3948	|| id:finn-b-I9_HYPTENS	TRUE	reported	igd	26.95156179
365	21	40289113	-0.0394	0.0093	2.35201E-05	finn-b-I9_HYPTENS	rs8128155	A	G	0.5976	|| id:finn-b-I9_HYPTENS	TRUE	reported	igd	17.94843334
366	21	45106294	0.0873	0.0193	6.25403E-06	finn-b-I9_HYPTENS	rs11701754	A	C	0.06043	|| id:finn-b-I9_HYPTENS	TRUE	reported	igd	20.4603882
367	21	34121944	-0.2317	0.0482	0.000001508	finn-b-I9_HYPTENS	rs139594626	A	G	0.00925	|| id:finn-b-I9_HYPTENS	TRUE	reported	igd	23.1077676
368	22	31563555	0.0475	0.0091	2.04899E-07	finn-b-I9_HYPTENS	rs5994376	C	A	0.539	|| id:finn-b-I9_HYPTENS	TRUE	reported	igd	27.24610554
369	22	46610999	0.3538	0.0786	6.84699E-06	finn-b-I9_HYPTENS	rs4253734	T	C	0.003499	|| id:finn-b-I9_HYPTENS	TRUE	reported	igd	20.261452
370	22	21290877	0.1719	0.0328	1.65699E-07	finn-b-I9_HYPTENS	rs149642022	C	G	0.02073	|| id:finn-b-I9_HYPTENS	TRUE	reported	igd	27.46654707

Adjust P-values and LD raw data-Obesity

	pval.exposure	beta.exposure	pos.exposure	se.exposure	chr.exposure	samplesize.exposure	id.exposure	SNP	effect_allele.exposure	other_allele.exposure	eaf.exposure	exposure	mr_keep.exposure	pval_origin.exposure	data_source.exposure	F statistics
1	2.39999E-06	0.11	219798632	0.023	1	70145	ieu-a-91	rs1415991	G	A	0.217	|| id:ieu-a-91	TRUE	reported	igd	22.87334594
2	4.79999E-09	0.11	72812440	0.02	1	71717	ieu-a-91	rs2815752	A	G	0.65	|| id:ieu-a-91	TRUE	reported	igd	30.25
3	0.000005	0.09	78349214	0.02	1	71589	ieu-a-91	rs9729667	T	C	0.425	|| id:ieu-a-91	TRUE	reported	igd	20.25
4	2.60016E-11	0.16	177852580	0.024	1	65464	ieu-a-91	rs633715	C	T	0.267	|| id:ieu-a-91	TRUE	reported	igd	44.44444444
5	0.0000089	0.13	229141933	0.029	1	63285	ieu-a-91	rs644850	G	A	0.833	|| id:ieu-a-91	TRUE	reported	igd	20.09512485
6	0.0000004	-0.1	49566557	0.02	1	71251	ieu-a-91	rs7527364	G	A	0.658	|| id:ieu-a-91	TRUE	reported	igd	25
7	1.89998E-07	0.12	202845785	0.024	1	67960	ieu-a-91	rs7538571	A	T	0.237	|| id:ieu-a-91	TRUE	reported	igd	25
8	4.09996E-05	0.079	207121329	0.019	2	71893	ieu-a-91	rs1198541	T	C	0.544	|| id:ieu-a-91	TRUE	reported	igd	17.28808864
9	5.80003E-07	-0.1	59305625	0.021	2	70866	ieu-a-91	rs1016287	C	T	0.689	|| id:ieu-a-91	TRUE	reported	igd	22.67573696
10	1.29987E-19	-0.24	650519	0.027	2	65303	ieu-a-91	rs13401686	G	A	0.125	|| id:ieu-a-91	TRUE	reported	igd	79.01234568
11	1.79999E-05	-0.088	211988980	0.021	2	70850	ieu-a-91	rs10804189	G	A	0.725	|| id:ieu-a-91	TRUE	reported	igd	17.5600907
12	0.0000015	0.094	227770727	0.02	2	71759	ieu-a-91	rs16822905	C	G	0.39	|| id:ieu-a-91	TRUE	reported	igd	22.09
13	1.79999E-07	0.16	58685318	0.031	2	59288	ieu-a-91	rs3732138	A	C	0.15	|| id:ieu-a-91	TRUE	reported	igd	26.63891779
14	1.09999E-06	0.093	25131986	0.019	2	71902	ieu-a-91	rs11900505	C	A	0.44	|| id:ieu-a-91	TRUE	reported	igd	23.95844875
15	2.39999E-05	0.083	42708405	0.02	2	71602	ieu-a-91	rs1979755	C	G	0.533	|| id:ieu-a-91	TRUE	reported	igd	17.2225
16	0.00002	0.097	164908052	0.023	2	67973	ieu-a-91	rs10497247	A	G	0.208	|| id:ieu-a-91	TRUE	reported	igd	17.78638941
17	1.2E-10	0.19	185824004	0.03	3	61014	ieu-a-91	rs1516725	C	T	0.908	|| id:ieu-a-91	TRUE	reported	igd	40.11111111
18	3.79997E-06	0.091	85683600	0.02	3	71072	ieu-a-91	rs9829032	G	A	0.432	|| id:ieu-a-91	TRUE	reported	igd	20.7025
19	3.50002E-05	0.08	80407900	0.019	3	71944	ieu-a-91	rs17386402	T	A	0.343	|| id:ieu-a-91	TRUE	reported	igd	17.72853186
20	2.90001E-05	0.091	143799966	0.022	4	70277	ieu-a-91	rs4690731	T	A	0.233	|| id:ieu-a-91	TRUE	reported	igd	17.10950413
21	2.19999E-05	0.088	171057186	0.021	4	70992	ieu-a-91	rs9992050	T	C	0.333	|| id:ieu-a-91	TRUE	reported	igd	17.5600907
22	1.6E-09	0.12	45175691	0.02	4	71761	ieu-a-91	rs13130484	T	C	0.424	|| id:ieu-a-91	TRUE	reported	igd	36
23	4.79999E-05	-0.088	4895135	0.022	4	70062	ieu-a-91	rs6823800	C	T	0.27	|| id:ieu-a-91	TRUE	reported	igd	16
24	3.29997E-05	0.079	38592867	0.019	4	72377	ieu-a-91	rs7678850	C	T	0.542	|| id:ieu-a-91	TRUE	reported	igd	17.28808864
25	1.79999E-05	0.22	103188709	0.052	4	42203	ieu-a-91	rs13107325	T	C	0.117	|| id:ieu-a-91	TRUE	reported	igd	17.89940828
26	0.0000025	0.13	77129568	0.028	4	61993	ieu-a-91	rs17001654	G	C	0.158	|| id:ieu-a-91	TRUE	reported	igd	21.55612245
27	3.69999E-07	-0.1	75015242	0.02	5	71951	ieu-a-91	rs2112347	G	T	0.381	|| id:ieu-a-91	TRUE	reported	igd	25
28	9.40005E-06	-0.088	31236051	0.02	6	71985	ieu-a-91	rs2524099	A	G	0.464	|| id:ieu-a-91	TRUE	reported	igd	19.36
29	8.19974E-12	0.17	50845490	0.025	6	62999	ieu-a-91	rs2207139	G	A	0.102	|| id:ieu-a-91	TRUE	reported	igd	46.24
30	4.90004E-05	0.081	164030800	0.02	6	71567	ieu-a-91	rs6941513	G	A	0.259	|| id:ieu-a-91	TRUE	reported	igd	16.4025
31	0.0000064	0.12	131897278	0.026	6	66800	ieu-a-91	rs2781668	T	C	0.108	|| id:ieu-a-91	TRUE	reported	igd	21.30177515
32	3.79997E-05	0.086	39973445	0.021	6	69568	ieu-a-91	rs2984433	G	T	0.642	|| id:ieu-a-91	TRUE	reported	igd	16.77097506
33	2.59998E-05	0.1	97414110	0.024	7	66270	ieu-a-91	rs460	T	C	0.142	|| id:ieu-a-91	TRUE	reported	igd	17.36111111
34	1.29999E-05	0.089	76721610	0.021	8	71127	ieu-a-91	rs2977321	T	C	0.625	|| id:ieu-a-91	TRUE	reported	igd	17.96145125
35	0.000017	0.099	106409193	0.023	8	69123	ieu-a-91	rs2343405	A	C	0.233	|| id:ieu-a-91	TRUE	reported	igd	18.52741021
36	3.59998E-06	0.089	79318921	0.019	9	71978	ieu-a-91	rs620985	G	A	0.627	|| id:ieu-a-91	TRUE	reported	igd	21.94182825
37	0.0000016	0.099	28425515	0.021	9	69704	ieu-a-91	rs1412239	G	C	0.292	|| id:ieu-a-91	TRUE	reported	igd	22.2244898
38	0.000017	-0.083	129460914	0.019	9	72355	ieu-a-91	rs10733682	G	A	0.568	|| id:ieu-a-91	TRUE	reported	igd	19.08310249
39	0.00002	-0.081	114768783	0.019	10	72343	ieu-a-91	rs6585201	A	G	0.408	|| id:ieu-a-91	TRUE	reported	igd	18.17451524
40	4.79999E-05	-0.077	102840235	0.019	10	71963	ieu-a-91	rs10786618	T	C	0.45	|| id:ieu-a-91	TRUE	reported	igd	16.42382271
41	1.79999E-06	-0.091	8522182	0.019	11	72289	ieu-a-91	rs10840063	C	T	0.424	|| id:ieu-a-91	TRUE	reported	igd	22.93905817
42	5.19996E-08	0.13	27728539	0.024	11	66556	ieu-a-91	rs2030323	C	A	0.783	|| id:ieu-a-91	TRUE	reported	igd	29.34027778
43	3.79997E-05	-0.088	47811309	0.021	11	70674	ieu-a-91	rs7114011	C	A	0.325	|| id:ieu-a-91	TRUE	reported	igd	17.5600907
44	4.79999E-05	-0.077	92444811	0.019	12	72356	ieu-a-91	rs2035950	G	A	0.525	|| id:ieu-a-91	TRUE	reported	igd	16.42382271
45	1.99986E-11	0.13	50247468	0.019	12	71541	ieu-a-91	rs7138803	A	G	0.442	|| id:ieu-a-91	TRUE	reported	igd	46.81440443
46	2.90001E-05	0.12	102640056	0.029	12	61155	ieu-a-91	rs6539029	A	G	0.125	|| id:ieu-a-91	TRUE	reported	igd	17.12247325
47	1.09999E-06	-0.12	122613000	0.025	12	66891	ieu-a-91	rs10847689	C	T	0.225	|| id:ieu-a-91	TRUE	reported	igd	23.04
48	3.09999E-05	-0.089	1075536	0.021	12	70579	ieu-a-91	rs11614523	C	T	0.425	|| id:ieu-a-91	TRUE	reported	igd	17.96145125
49	7.49998E-06	0.13	54107352	0.03	13	61777	ieu-a-91	rs9568867	A	G	0.1	|| id:ieu-a-91	TRUE	reported	igd	18.77777778
50	2.99999E-06	0.21	28416179	0.046	13	47813	ieu-a-91	rs9512893	A	G	0.025	|| id:ieu-a-91	TRUE	reported	igd	20.84120983
51	5.89997E-09	0.11	97017548	0.019	13	71938	ieu-a-91	rs7989336	A	G	0.425	|| id:ieu-a-91	TRUE	reported	igd	33.51800554
52	6.80002E-06	0.085	79899454	0.019	14	72326	ieu-a-91	rs7141420	T	C	0.621	|| id:ieu-a-91	TRUE	reported	igd	20.01385042
53	2.30001E-05	0.086	33302882	0.02	14	68661	ieu-a-91	rs17522122	T	G	0.467	|| id:ieu-a-91	TRUE	reported	igd	18.49
54	2.30001E-05	-0.18	84042347	0.042	14	51270	ieu-a-91	rs9635272	T	C	0.102	|| id:ieu-a-91	TRUE	reported	igd	18.36734694
55	4.30002E-05	0.084	59813702	0.021	14	70552	ieu-a-91	rs8008151	T	C	0.708	|| id:ieu-a-91	TRUE	reported	igd	16
56	0.000002	-0.092	95257956	0.019	15	71943	ieu-a-91	rs7176675	C	T	0.583	|| id:ieu-a-91	TRUE	reported	igd	23.44598338
57	4.70002E-05	0.078	24236088	0.019	15	71944	ieu-a-91	rs1459968	C	A	0.586	|| id:ieu-a-91	TRUE	reported	igd	16.8531856
58	2.39999E-07	0.099	68019958	0.019	15	71919	ieu-a-91	rs12914773	A	G	0.562	|| id:ieu-a-91	TRUE	reported	igd	27.14958449
59	3.80014E-51	0.29	53816752	0.019	16	71976	ieu-a-91	rs8051591	G	A	0.448	|| id:ieu-a-91	TRUE	reported	igd	232.9639889
60	3.29997E-05	-0.17	83014746	0.04	16	46906	ieu-a-91	rs12931405	A	T	0.069	|| id:ieu-a-91	TRUE	reported	igd	18.0625
61	0.00000051	-0.11	28921809	0.022	16	70459	ieu-a-91	rs7184597	C	T	0.729	|| id:ieu-a-91	TRUE	reported	igd	25
62	4.60002E-05	0.081	72997747	0.02	16	72336	ieu-a-91	rs4788683	T	G	0.425	|| id:ieu-a-91	TRUE	reported	igd	16.4025
63	0.000032	0.081	31131614	0.02	16	71976	ieu-a-91	rs4527034	G	A	0.612	|| id:ieu-a-91	TRUE	reported	igd	16.4025
64	6.80002E-09	-0.17	19933600	0.03	16	62287	ieu-a-91	rs12444979	T	C	0.06	|| id:ieu-a-91	TRUE	reported	igd	32.11111111
65	1.40001E-05	0.1	3627358	0.023	16	68762	ieu-a-91	rs758747	T	C	0.263	|| id:ieu-a-91	TRUE	reported	igd	18.90359168
66	5.30005E-06	-0.13	46080233	0.028	17	64431	ieu-a-91	rs8071778	C	G	0.125	|| id:ieu-a-91	TRUE	reported	igd	21.55612245
67	6.90001E-07	0.1	45356561	0.02	17	71289	ieu-a-91	rs1000232	C	T	0.337	|| id:ieu-a-91	TRUE	reported	igd	25
68	1.09999E-05	-0.13	1843865	0.029	17	63649	ieu-a-91	rs4790845	C	G	0.158	|| id:ieu-a-91	TRUE	reported	igd	20.09512485
69	2.99999E-05	-0.078	34946547	0.019	17	72349	ieu-a-91	rs12603276	C	T	0.467	|| id:ieu-a-91	TRUE	reported	igd	16.8531856
70	5.30029E-14	0.17	57851763	0.023	18	68784	ieu-a-91	rs10871777	G	A	0.283	|| id:ieu-a-91	TRUE	reported	igd	54.63137996
71	3.40001E-06	-0.18	58049656	0.04	18	48793	ieu-a-91	rs17066856	C	T	0.133	|| id:ieu-a-91	TRUE	reported	igd	20.25
72	4.20001E-05	-0.089	40761976	0.022	18	69819	ieu-a-91	rs7231852	A	G	0.25	|| id:ieu-a-91	TRUE	reported	igd	16.36570248
73	2.39999E-05	0.089	71482929	0.021	18	70440	ieu-a-91	rs12455621	T	C	0.375	|| id:ieu-a-91	TRUE	reported	igd	17.96145125
74	0.0000021	0.096	34311481	0.02	19	71108	ieu-a-91	rs29938	C	T	0.653	|| id:ieu-a-91	TRUE	reported	igd	23.04
75	1.09999E-06	-0.13	46182304	0.027	19	62395	ieu-a-91	rs10423928	A	T	0.176	|| id:ieu-a-91	TRUE	reported	igd	23.1824417
76	3.69999E-05	0.41	3012883	0.1	19	33994	ieu-a-91	rs216269	A	G	0.025	|| id:ieu-a-91	TRUE	reported	igd	16.81
77	1.29999E-05	0.16	21451139	0.037	20	46965	ieu-a-91	rs813393	A	G	0.108	|| id:ieu-a-91	TRUE	reported	igd	18.69978086

Adjust P-values and LD raw data-OSA

	pos.exposure	pval.exposure	se.exposure	chr.exposure	beta.exposure	id.exposure	SNP	effect_allele.exposure	other_allele.exposure	eaf.exposure	exposure	mr_keep.exposure	pval_origin.exposure	data_source.exposure	F statistic
1	21045726	3.33803E-05	0.0125	1	-0.0521	finn-b-G6_SLEEPAPNO	rs667071	C	T	0.4435	|| id:finn-b-G6_SLEEPAPNO	TRUE	reported	igd	17.372224
2	44544264	1.62099E-05	0.0418	1	0.18	finn-b-G6_SLEEPAPNO	rs140929616	C	T	0.02298	|| id:finn-b-G6_SLEEPAPNO	TRUE	reported	igd	18.54353151
3	165919377	1.62798E-05	0.0314	1	0.1353	finn-b-G6_SLEEPAPNO	rs72701937	A	G	0.04162	|| id:finn-b-G6_SLEEPAPNO	TRUE	reported	igd	18.56676741
4	243295818	7.48307E-06	0.0301	1	-0.1347	finn-b-G6_SLEEPAPNO	rs139889222	G	T	0.04761	|| id:finn-b-G6_SLEEPAPNO	TRUE	reported	igd	20.02636836
5	42139393	1.74699E-05	0.0153	1	-0.0656	finn-b-G6_SLEEPAPNO	rs17372813	T	C	0.217	|| id:finn-b-G6_SLEEPAPNO	TRUE	reported	igd	18.38335683
6	4829795	3.14797E-05	0.021	1	0.0873	finn-b-G6_SLEEPAPNO	rs3950181	T	C	0.09792	|| id:finn-b-G6_SLEEPAPNO	TRUE	reported	igd	17.28183673
7	51585052	2.76898E-05	0.024	1	0.1005	finn-b-G6_SLEEPAPNO	rs56073003	G	A	0.07291	|| id:finn-b-G6_SLEEPAPNO	TRUE	reported	igd	17.53515625
8	7167673	4.39704E-05	0.0267	1	-0.109	finn-b-G6_SLEEPAPNO	rs72640096	T	C	0.05914	|| id:finn-b-G6_SLEEPAPNO	TRUE	reported	igd	16.6659653
9	203027240	4.03302E-05	0.0157	1	0.0644	finn-b-G6_SLEEPAPNO	rs10920556	G	A	0.1968	|| id:finn-b-G6_SLEEPAPNO	TRUE	reported	igd	16.82567244
10	209253452	0.000007335	0.04	1	0.1795	finn-b-G6_SLEEPAPNO	rs61819581	C	G	0.02473	|| id:finn-b-G6_SLEEPAPNO	TRUE	reported	igd	20.13765625
11	88064857	8.89201E-06	0.0159	1	-0.0706	finn-b-G6_SLEEPAPNO	rs72953990	A	G	0.1932	|| id:finn-b-G6_SLEEPAPNO	TRUE	reported	igd	19.71583403
12	23169503	2.93799E-05	0.0165	1	0.0691	finn-b-G6_SLEEPAPNO	rs4655128	C	T	0.8269	|| id:finn-b-G6_SLEEPAPNO	TRUE	reported	igd	17.53832874
13	213891373	0.00002524	0.0306	1	0.129	finn-b-G6_SLEEPAPNO	rs72751562	T	C	0.04249	|| id:finn-b-G6_SLEEPAPNO	TRUE	reported	igd	17.77201077
14	225630413	0.00002613	0.0135	1	-0.0568	finn-b-G6_SLEEPAPNO	rs12127040	T	C	0.311	|| id:finn-b-G6_SLEEPAPNO	TRUE	reported	igd	17.70227709
15	237655267	4.26599E-05	0.05	1	-0.2045	finn-b-G6_SLEEPAPNO	rs188369875	C	T	0.01673	|| id:finn-b-G6_SLEEPAPNO	TRUE	reported	igd	16.7281
16	152838914	1.20099E-05	0.0425	1	0.1858	finn-b-G6_SLEEPAPNO	rs115867607	T	G	0.02241	|| id:finn-b-G6_SLEEPAPNO	TRUE	reported	igd	19.11232664
17	2394648	4.12601E-06	0.0348	1	0.1601	finn-b-G6_SLEEPAPNO	rs10910079	T	C	0.03324	|| id:finn-b-G6_SLEEPAPNO	TRUE	reported	igd	21.16528769
18	53977292	1.536E-07	0.0238	1	0.1246	finn-b-G6_SLEEPAPNO	rs527014	T	C	0.07433	|| id:finn-b-G6_SLEEPAPNO	TRUE	reported	igd	27.4083045
19	59772059	8.688E-07	0.0163	2	-0.0801	finn-b-G6_SLEEPAPNO	rs996762	G	C	0.8239	|| id:finn-b-G6_SLEEPAPNO	TRUE	reported	igd	24.14848131
20	102597447	3.26603E-05	0.0469	2	0.1947	finn-b-G6_SLEEPAPNO	rs72817832	C	T	0.01732	|| id:finn-b-G6_SLEEPAPNO	TRUE	reported	igd	17.23400512
21	62766723	4.08197E-05	0.0231	2	0.0948	finn-b-G6_SLEEPAPNO	rs11904315	A	C	0.07858	|| id:finn-b-G6_SLEEPAPNO	TRUE	reported	igd	16.84196323
22	137163357	4.08696E-05	0.0126	2	0.0517	finn-b-G6_SLEEPAPNO	rs1430357	C	A	0.5217	|| id:finn-b-G6_SLEEPAPNO	TRUE	reported	igd	16.83604182
23	173720268	3.68604E-05	0.0226	2	0.0933	finn-b-G6_SLEEPAPNO	rs956752	G	A	0.08183	|| id:finn-b-G6_SLEEPAPNO	TRUE	reported	igd	17.04301433
24	37557873	3.89601E-05	0.0972	2	0.3998	finn-b-G6_SLEEPAPNO	rs137945153	T	A	0.003997	|| id:finn-b-G6_SLEEPAPNO	TRUE	reported	igd	16.91815695
25	104387855	2.27002E-05	0.0126	2	-0.0532	finn-b-G6_SLEEPAPNO	rs671722	A	G	0.5719	|| id:finn-b-G6_SLEEPAPNO	TRUE	reported	igd	17.82716049
26	136991807	2.80201E-08	0.0158	2	-0.0878	finn-b-G6_SLEEPAPNO	rs10928560	T	C	0.1949	|| id:finn-b-G6_SLEEPAPNO	TRUE	reported	igd	30.87982695
27	56337991	1.62301E-05	0.026	2	0.1121	finn-b-G6_SLEEPAPNO	rs7608469	T	A	0.06	|| id:finn-b-G6_SLEEPAPNO	TRUE	reported	igd	18.58936391
28	174094145	4.11197E-05	0.0165	2	0.0675	finn-b-G6_SLEEPAPNO	rs10182247	C	T	0.1737	|| id:finn-b-G6_SLEEPAPNO	TRUE	reported	igd	16.73553719
29	18626280	2.36499E-05	0.0306	2	-0.1294	finn-b-G6_SLEEPAPNO	rs13393878	C	T	0.04414	|| id:finn-b-G6_SLEEPAPNO	TRUE	reported	igd	17.88239566
30	88754727	3.90697E-06	0.0475	3	-0.2193	finn-b-G6_SLEEPAPNO	rs181190675	T	C	0.01815	|| id:finn-b-G6_SLEEPAPNO	TRUE	reported	igd	21.31523102
31	146780513	7.45796E-06	0.0795	3	0.3561	finn-b-G6_SLEEPAPNO	rs77998028	T	C	0.006318	|| id:finn-b-G6_SLEEPAPNO	TRUE	reported	igd	20.06363831
32	17996736	8.63197E-06	0.0272	3	0.1211	finn-b-G6_SLEEPAPNO	rs116497662	A	C	0.05604	|| id:finn-b-G6_SLEEPAPNO	TRUE	reported	igd	19.82213722
33	132697086	6.34805E-06	0.0129	3	0.0581	finn-b-G6_SLEEPAPNO	rs4622901	G	A	0.6328	|| id:finn-b-G6_SLEEPAPNO	TRUE	reported	igd	20.28489874
34	168616977	8.67901E-06	0.0127	3	-0.0565	finn-b-G6_SLEEPAPNO	rs6791437	G	A	0.4097	|| id:finn-b-G6_SLEEPAPNO	TRUE	reported	igd	19.79198958
35	60977414	3.85097E-05	0.0197	3	-0.0809	finn-b-G6_SLEEPAPNO	rs9819332	C	T	0.1138	|| id:finn-b-G6_SLEEPAPNO	TRUE	reported	igd	16.86415522
36	188945423	0.000009621	0.0136	3	-0.06	finn-b-G6_SLEEPAPNO	rs6773697	G	A	0.3171	|| id:finn-b-G6_SLEEPAPNO	TRUE	reported	igd	19.46366782
37	119062265	3.78103E-05	0.0403	3	0.1662	finn-b-G6_SLEEPAPNO	rs115777037	G	A	0.02406	|| id:finn-b-G6_SLEEPAPNO	TRUE	reported	igd	17.00794907
38	6221580	0.00001213	0.0895	3	0.3914	finn-b-G6_SLEEPAPNO	rs149473233	A	G	0.005354	|| id:finn-b-G6_SLEEPAPNO	TRUE	reported	igd	19.12474143
39	44663014	2.54601E-05	0.0149	3	-0.0625	finn-b-G6_SLEEPAPNO	rs9815769	T	A	0.2253	|| id:finn-b-G6_SLEEPAPNO	TRUE	reported	igd	17.59492816
40	132178290	0.00001335	0.0237	3	-0.1033	finn-b-G6_SLEEPAPNO	rs9858475	C	T	0.07681	|| id:finn-b-G6_SLEEPAPNO	TRUE	reported	igd	18.99782798
41	136752590	1.42099E-05	0.0151	3	0.0657	finn-b-G6_SLEEPAPNO	rs13099657	A	G	0.2172	|| id:finn-b-G6_SLEEPAPNO	TRUE	reported	igd	18.93114337
42	30274135	2.69501E-05	0.033	3	0.1384	finn-b-G6_SLEEPAPNO	rs62242228	T	C	0.03731	|| id:finn-b-G6_SLEEPAPNO	TRUE	reported	igd	17.58912764
43	87858977	2.83302E-05	0.0137	3	0.0576	finn-b-G6_SLEEPAPNO	rs2932270	T	C	0.2851	|| id:finn-b-G6_SLEEPAPNO	TRUE	reported	igd	17.6768075
44	158973533	1.56801E-05	0.0674	4	-0.2909	finn-b-G6_SLEEPAPNO	rs72689944	A	G	0.009086	|| id:finn-b-G6_SLEEPAPNO	TRUE	reported	igd	18.62806091
45	185546497	8.36007E-07	0.0544	4	0.2679	finn-b-G6_SLEEPAPNO	rs182846984	T	G	0.01414	|| id:finn-b-G6_SLEEPAPNO	TRUE	reported	igd	24.25200381
46	135390801	4.37603E-05	0.0463	4	-0.1892	finn-b-G6_SLEEPAPNO	rs75318986	G	A	0.01909	|| id:finn-b-G6_SLEEPAPNO	TRUE	reported	igd	16.69860847
47	92350342	3.53199E-06	0.0127	4	0.0588	finn-b-G6_SLEEPAPNO	rs6845679	T	C	0.5898	|| id:finn-b-G6_SLEEPAPNO	TRUE	reported	igd	21.43617087
48	88934955	3.99503E-06	0.0166	4	-0.0766	finn-b-G6_SLEEPAPNO	rs2725231	G	A	0.8336	|| id:finn-b-G6_SLEEPAPNO	TRUE	reported	igd	21.29322108
49	45850116	4.56899E-05	0.0987	4	0.4022	finn-b-G6_SLEEPAPNO	rs7684745	G	A	0.003935	|| id:finn-b-G6_SLEEPAPNO	TRUE	reported	igd	16.60541857
50	122268332	3.77199E-05	0.0335	4	0.1379	finn-b-G6_SLEEPAPNO	rs62319031	G	A	0.03592	|| id:finn-b-G6_SLEEPAPNO	TRUE	reported	igd	16.94489641
51	26468820	0.00001557	0.0202	4	-0.0873	finn-b-G6_SLEEPAPNO	rs716073	A	C	0.1073	|| id:finn-b-G6_SLEEPAPNO	TRUE	reported	igd	18.6778012
52	37761713	7.43105E-06	0.137	4	0.6137	finn-b-G6_SLEEPAPNO	rs80245059	A	C	0.002313	|| id:finn-b-G6_SLEEPAPNO	TRUE	reported	igd	20.0664761
53	45186139	3.63898E-06	0.0125	4	0.0577	finn-b-G6_SLEEPAPNO	rs10938398	A	G	0.4731	|| id:finn-b-G6_SLEEPAPNO	TRUE	reported	igd	21.307456
54	125311740	4.82003E-06	0.0251	4	-0.1146	finn-b-G6_SLEEPAPNO	rs78730556	T	C	0.06738	|| id:finn-b-G6_SLEEPAPNO	TRUE	reported	igd	20.84595483
55	34674725	0.00001344	0.0139	5	-0.0605	finn-b-G6_SLEEPAPNO	rs256291	T	C	0.7106	|| id:finn-b-G6_SLEEPAPNO	TRUE	reported	igd	18.94441282
56	170859228	3.94903E-06	0.0125	5	-0.0577	finn-b-G6_SLEEPAPNO	rs10475978	C	G	0.4904	|| id:finn-b-G6_SLEEPAPNO	TRUE	reported	igd	21.307456
57	115082642	1.37899E-05	0.0931	5	0.4049	finn-b-G6_SLEEPAPNO	rs150153139	A	G	0.004463	|| id:finn-b-G6_SLEEPAPNO	TRUE	reported	igd	18.91455776
58	4839617	1.29999E-05	0.0423	5	0.1846	finn-b-G6_SLEEPAPNO	rs10512749	T	G	0.0222	|| id:finn-b-G6_SLEEPAPNO	TRUE	reported	igd	19.04507374
59	154685918	7.92994E-06	0.0734	5	-0.3277	finn-b-G6_SLEEPAPNO	rs115708455	T	C	0.007741	|| id:finn-b-G6_SLEEPAPNO	TRUE	reported	igd	19.93245365
60	155474649	2.17801E-05	0.0374	5	-0.1588	finn-b-G6_SLEEPAPNO	rs62380697	T	C	0.03001	|| id:finn-b-G6_SLEEPAPNO	TRUE	reported	igd	18.02842518
61	73570263	0.00004689	0.0378	5	-0.154	finn-b-G6_SLEEPAPNO	rs7709654	T	C	0.02888	|| id:finn-b-G6_SLEEPAPNO	TRUE	reported	igd	16.59807956
62	168027543	6.15602E-06	0.0415	5	-0.1875	finn-b-G6_SLEEPAPNO	rs62385672	C	G	0.02366	|| id:finn-b-G6_SLEEPAPNO	TRUE	reported	igd	20.41297721
63	168353247	1.69801E-05	0.0128	5	0.0551	finn-b-G6_SLEEPAPNO	rs891959	C	T	0.408	|| id:finn-b-G6_SLEEPAPNO	TRUE	reported	igd	18.53033447
64	140749326	0.00001895	0.0211	5	-0.0904	finn-b-G6_SLEEPAPNO	rs6873480	A	G	0.09707	|| id:finn-b-G6_SLEEPAPNO	TRUE	reported	igd	18.35574223
65	95865500	2.13801E-05	0.0125	5	-0.0532	finn-b-G6_SLEEPAPNO	rs13186522	C	T	0.4455	|| id:finn-b-G6_SLEEPAPNO	TRUE	reported	igd	18.113536
66	132128872	0.00001065	0.071	5	-0.3129	finn-b-G6_SLEEPAPNO	rs138749219	T	A	0.008208	|| id:finn-b-G6_SLEEPAPNO	TRUE	reported	igd	19.42202142
67	7746663	0.00001022	0.015	6	0.0664	finn-b-G6_SLEEPAPNO	rs60700772	C	T	0.222	|| id:finn-b-G6_SLEEPAPNO	TRUE	reported	igd	19.59537778
68	4482102	3.90904E-05	0.0137	6	-0.0565	finn-b-G6_SLEEPAPNO	rs6914063	T	C	0.2902	|| id:finn-b-G6_SLEEPAPNO	TRUE	reported	igd	17.00809846
69	43813586	2.97797E-06	0.0129	6	0.0602	finn-b-G6_SLEEPAPNO	rs11758441	T	C	0.378	|| id:finn-b-G6_SLEEPAPNO	TRUE	reported	igd	21.77777778
70	31312664	9.42302E-06	0.0241	6	-0.1068	finn-b-G6_SLEEPAPNO	rs9265860	A	G	0.07355	|| id:finn-b-G6_SLEEPAPNO	TRUE	reported	igd	19.63850485
71	96762759	8.90902E-06	0.0164	6	0.073	finn-b-G6_SLEEPAPNO	rs75901551	T	C	0.1741	|| id:finn-b-G6_SLEEPAPNO	TRUE	reported	igd	19.81335515
72	155346272	0.00003992	0.0131	6	-0.0538	finn-b-G6_SLEEPAPNO	rs9371849	T	C	0.3504	|| id:finn-b-G6_SLEEPAPNO	TRUE	reported	igd	16.86638308
73	160832905	1.61399E-05	0.0546	6	-0.2355	finn-b-G6_SLEEPAPNO	rs189839402	A	G	0.01345	|| id:finn-b-G6_SLEEPAPNO	TRUE	reported	igd	18.60358049
74	42367754	1.30101E-05	0.164	6	0.7152	finn-b-G6_SLEEPAPNO	rs114457753	C	G	0.001608	|| id:finn-b-G6_SLEEPAPNO	TRUE	reported	igd	19.01810827
75	113984567	8.18597E-06	0.0198	6	-0.0883	finn-b-G6_SLEEPAPNO	rs12205015	T	A	0.1124	|| id:finn-b-G6_SLEEPAPNO	TRUE	reported	igd	19.88799612
76	143725481	2.09498E-05	0.0235	6	0.1001	finn-b-G6_SLEEPAPNO	rs223592	T	C	0.07919	|| id:finn-b-G6_SLEEPAPNO	TRUE	reported	igd	18.14397465
77	30105154	3.80803E-05	0.0752	6	0.3099	finn-b-G6_SLEEPAPNO	rs28780086	G	A	0.006725	|| id:finn-b-G6_SLEEPAPNO	TRUE	reported	igd	16.98272868
78	90661576	0.00003182	0.0128	6	0.0531	finn-b-G6_SLEEPAPNO	rs2236181	C	T	0.3964	|| id:finn-b-G6_SLEEPAPNO	TRUE	reported	igd	17.20953369
79	150533765	4.49697E-05	0.0128	7	0.0523	finn-b-G6_SLEEPAPNO	rs10952291	A	C	0.3778	|| id:finn-b-G6_SLEEPAPNO	TRUE	reported	igd	16.69488525
80	25907560	2.37799E-05	0.0168	7	0.0708	finn-b-G6_SLEEPAPNO	rs7804889	G	A	0.1667	|| id:finn-b-G6_SLEEPAPNO	TRUE	reported	igd	17.76020408
81	140330281	1.91501E-05	0.0128	7	-0.0545	finn-b-G6_SLEEPAPNO	rs269264	T	G	0.6051	|| id:finn-b-G6_SLEEPAPNO	TRUE	reported	igd	18.12896729
82	154199573	1.01599E-05	0.0124	7	-0.0549	finn-b-G6_SLEEPAPNO	rs2337376	G	A	0.4836	|| id:finn-b-G6_SLEEPAPNO	TRUE	reported	igd	19.60204214
83	83829199	9.62011E-06	0.043	7	0.1903	finn-b-G6_SLEEPAPNO	rs76701535	A	G	0.02188	|| id:finn-b-G6_SLEEPAPNO	TRUE	reported	igd	19.58577069
84	14616970	1.11599E-05	0.0129	7	0.0569	finn-b-G6_SLEEPAPNO	rs12699649	T	C	0.3786	|| id:finn-b-G6_SLEEPAPNO	TRUE	reported	igd	19.45562166
85	2018870	2.90797E-07	0.0148	7	-0.0757	finn-b-G6_SLEEPAPNO	rs3996329	A	G	0.2366	|| id:finn-b-G6_SLEEPAPNO	TRUE	reported	igd	26.16184259
86	108054342	0.00001862	0.1139	7	0.4878	finn-b-G6_SLEEPAPNO	rs148251400	T	G	0.003196	|| id:finn-b-G6_SLEEPAPNO	TRUE	reported	igd	18.34155463
87	94786567	0.00002594	0.0422	7	-0.1776	finn-b-G6_SLEEPAPNO	rs79856499	A	C	0.02277	|| id:finn-b-G6_SLEEPAPNO	TRUE	reported	igd	17.71173154
88	54115304	7.48204E-06	0.0139	8	0.0622	finn-b-G6_SLEEPAPNO	rs1425898	G	A	0.6756	|| id:finn-b-G6_SLEEPAPNO	TRUE	reported	igd	20.02401532
89	41213526	2.16701E-05	0.0598	8	-0.2538	finn-b-G6_SLEEPAPNO	rs149045173	T	C	0.01142	|| id:finn-b-G6_SLEEPAPNO	TRUE	reported	igd	18.01278509
90	19935084	3.22003E-05	0.0211	8	0.0875	finn-b-G6_SLEEPAPNO	rs13281248	A	G	0.09682	|| id:finn-b-G6_SLEEPAPNO	TRUE	reported	igd	17.19694077
91	110101683	2.27898E-05	0.0127	8	-0.0539	finn-b-G6_SLEEPAPNO	rs3134105	G	A	0.6089	|| id:finn-b-G6_SLEEPAPNO	TRUE	reported	igd	18.01233802
92	133465431	3.36698E-05	0.0305	8	0.1264	finn-b-G6_SLEEPAPNO	rs10105036	C	G	0.0442	|| id:finn-b-G6_SLEEPAPNO	TRUE	reported	igd	17.17490997
93	76799375	1.03001E-05	0.0327	8	0.1443	finn-b-G6_SLEEPAPNO	rs141847080	T	C	0.03746	|| id:finn-b-G6_SLEEPAPNO	TRUE	reported	igd	19.47319249
94	101473404	2.09802E-06	0.0146	9	-0.0693	finn-b-G6_SLEEPAPNO	rs405430	T	G	0.7625	|| id:finn-b-G6_SLEEPAPNO	TRUE	reported	igd	22.52997748
95	28410996	2.09098E-05	0.0129	9	0.0547	finn-b-G6_SLEEPAPNO	rs1412235	C	G	0.3759	|| id:finn-b-G6_SLEEPAPNO	TRUE	reported	igd	17.98022955
96	128141809	1.527E-08	0.0125	9	-0.0706	finn-b-G6_SLEEPAPNO	rs4837016	A	G	0.4662	|| id:finn-b-G6_SLEEPAPNO	TRUE	reported	igd	31.899904
97	91235248	2.64399E-05	0.0143	9	-0.0599	finn-b-G6_SLEEPAPNO	rs28393561	C	A	0.2572	|| id:finn-b-G6_SLEEPAPNO	TRUE	reported	igd	17.54613918
98	119330060	0.00001793	0.0124	9	-0.0532	finn-b-G6_SLEEPAPNO	rs10983236	G	T	0.5085	|| id:finn-b-G6_SLEEPAPNO	TRUE	reported	igd	18.40686785
99	16683543	1.62401E-06	0.02	9	0.0959	finn-b-G6_SLEEPAPNO	rs4961731	T	C	0.8894	|| id:finn-b-G6_SLEEPAPNO	TRUE	reported	igd	22.992025
100	110379567	0.000001494	0.0262	9	-0.1262	finn-b-G6_SLEEPAPNO	rs12682930	T	G	0.0609	|| id:finn-b-G6_SLEEPAPNO	TRUE	reported	igd	23.20150341
101	453964	2.12501E-05	0.0541	10	-0.2302	finn-b-G6_SLEEPAPNO	rs79900977	C	A	0.01385	|| id:finn-b-G6_SLEEPAPNO	TRUE	reported	igd	18.10573286
102	91067438	7.52991E-06	0.0155	10	0.0695	finn-b-G6_SLEEPAPNO	rs4934470	C	T	0.7976	|| id:finn-b-G6_SLEEPAPNO	TRUE	reported	igd	20.10509886
103	95759405	9.20005E-06	0.0151	10	-0.0672	finn-b-G6_SLEEPAPNO	rs1223589	A	G	0.2159	|| id:finn-b-G6_SLEEPAPNO	TRUE	reported	igd	19.80544713
104	78186463	3.87998E-05	0.0125	10	0.0513	finn-b-G6_SLEEPAPNO	rs9664134	A	G	0.4717	|| id:finn-b-G6_SLEEPAPNO	TRUE	reported	igd	16.842816
105	94899739	4.81704E-05	0.0166	10	-0.0673	finn-b-G6_SLEEPAPNO	rs4918428	A	G	0.8306	|| id:finn-b-G6_SLEEPAPNO	TRUE	reported	igd	16.43667441
106	25227403	1.68399E-05	0.0139	10	-0.0599	finn-b-G6_SLEEPAPNO	rs12246876	C	T	0.2837	|| id:finn-b-G6_SLEEPAPNO	TRUE	reported	igd	18.57051912
107	14805937	7.33196E-06	0.0142	10	-0.0638	finn-b-G6_SLEEPAPNO	rs61842991	C	T	0.2607	|| id:finn-b-G6_SLEEPAPNO	TRUE	reported	igd	20.18666931
108	57381308	8.78901E-06	0.1223	10	-0.5435	finn-b-G6_SLEEPAPNO	rs116528678	A	G	0.002939	|| id:finn-b-G6_SLEEPAPNO	TRUE	reported	igd	19.74904879
109	28887992	3.04698E-05	0.0501	10	-0.2089	finn-b-G6_SLEEPAPNO	rs75114110	G	A	0.0162	|| id:finn-b-G6_SLEEPAPNO	TRUE	reported	igd	17.38607018
110	74057678	2.16601E-05	0.0287	11	0.1218	finn-b-G6_SLEEPAPNO	rs148567465	T	C	0.04918	|| id:finn-b-G6_SLEEPAPNO	TRUE	reported	igd	18.01070791
111	80372727	2.58702E-05	0.0553	11	-0.2326	finn-b-G6_SLEEPAPNO	rs72944941	A	C	0.0135	|| id:finn-b-G6_SLEEPAPNO	TRUE	reported	igd	17.69168337
112	28457737	9.13293E-06	0.0124	11	0.0551	finn-b-G6_SLEEPAPNO	rs4244535	T	C	0.4989	|| id:finn-b-G6_SLEEPAPNO	TRUE	reported	igd	19.74512227
113	27385916	2.10499E-05	0.0201	11	0.0854	finn-b-G6_SLEEPAPNO	rs78081623	C	G	0.1072	|| id:finn-b-G6_SLEEPAPNO	TRUE	reported	igd	18.05192941
114	100364301	0.000003539	0.0141	11	0.0655	finn-b-G6_SLEEPAPNO	rs11530654	C	A	0.2633	|| id:finn-b-G6_SLEEPAPNO	TRUE	reported	igd	21.57964891
115	106911624	3.24698E-05	0.0215	11	-0.0894	finn-b-G6_SLEEPAPNO	rs12806895	A	G	0.09425	|| id:finn-b-G6_SLEEPAPNO	TRUE	reported	igd	17.29012439
116	82886079	1.29799E-05	0.1109	11	-0.4837	finn-b-G6_SLEEPAPNO	rs113947253	G	T	0.003621	|| id:finn-b-G6_SLEEPAPNO	TRUE	reported	igd	19.02344129
117	133552291	3.34503E-05	0.0191	11	-0.079	finn-b-G6_SLEEPAPNO	rs11608087	A	G	0.1256	|| id:finn-b-G6_SLEEPAPNO	TRUE	reported	igd	17.10753543
118	16653971	1.87202E-06	0.0241	11	0.1148	finn-b-G6_SLEEPAPNO	rs72632980	C	T	0.07221	|| id:finn-b-G6_SLEEPAPNO	TRUE	reported	igd	22.69079389
119	47384420	2.18499E-05	0.0159	12	0.0677	finn-b-G6_SLEEPAPNO	rs2522262	T	C	0.1847	|| id:finn-b-G6_SLEEPAPNO	TRUE	reported	igd	18.1293857
120	57146069	2.16501E-05	0.0187	12	-0.0796	finn-b-G6_SLEEPAPNO	rs2277339	G	T	0.1292	|| id:finn-b-G6_SLEEPAPNO	TRUE	reported	igd	18.11936286
121	66885712	4.04697E-05	0.0535	12	0.2197	finn-b-G6_SLEEPAPNO	rs76364222	C	G	0.01369	|| id:finn-b-G6_SLEEPAPNO	TRUE	reported	igd	16.86368766
122	124802638	1.69801E-05	0.0127	12	0.0548	finn-b-G6_SLEEPAPNO	rs4765544	A	G	0.3997	|| id:finn-b-G6_SLEEPAPNO	TRUE	reported	igd	18.61888524
123	97753152	2.79705E-11	0.0163	12	0.1085	finn-b-G6_SLEEPAPNO	rs10507084	T	C	0.1793	|| id:finn-b-G6_SLEEPAPNO	TRUE	reported	igd	44.30821634
124	25534041	3.32598E-05	0.0136	12	-0.0565	finn-b-G6_SLEEPAPNO	rs1472036	T	C	0.6996	|| id:finn-b-G6_SLEEPAPNO	TRUE	reported	igd	17.25913711
125	102931304	5.10799E-06	0.0138	12	-0.063	finn-b-G6_SLEEPAPNO	rs7312617	G	A	0.7158	|| id:finn-b-G6_SLEEPAPNO	TRUE	reported	igd	20.84120983
126	55963016	9.23996E-07	0.0126	12	0.062	finn-b-G6_SLEEPAPNO	rs1896039	A	G	0.5349	|| id:finn-b-G6_SLEEPAPNO	TRUE	reported	igd	24.21264802
127	77859299	3.95804E-05	0.0511	12	-0.2101	finn-b-G6_SLEEPAPNO	rs73142209	C	T	0.0156	|| id:finn-b-G6_SLEEPAPNO	TRUE	reported	igd	16.90481041
128	97671568	2.01201E-06	0.0137	12	-0.0653	finn-b-G6_SLEEPAPNO	rs10860169	G	A	0.2906	|| id:finn-b-G6_SLEEPAPNO	TRUE	reported	igd	22.71879162
129	33017043	5.24204E-06	0.0256	12	0.1164	finn-b-G6_SLEEPAPNO	rs1454933	C	A	0.06345	|| id:finn-b-G6_SLEEPAPNO	TRUE	reported	igd	20.67407227
130	109015760	0.0000253	0.0171	13	0.0722	finn-b-G6_SLEEPAPNO	rs17381815	C	T	0.1555	|| id:finn-b-G6_SLEEPAPNO	TRUE	reported	igd	17.82716049
131	82181923	4.98597E-06	0.2164	13	0.9882	finn-b-G6_SLEEPAPNO	rs9318788	G	A	0.001152	|| id:finn-b-G6_SLEEPAPNO	TRUE	reported	igd	20.85331897
132	23181364	3.08198E-06	0.0176	13	0.0821	finn-b-G6_SLEEPAPNO	rs9510253	T	A	0.1468	|| id:finn-b-G6_SLEEPAPNO	TRUE	reported	igd	21.7601046
133	51365968	0.00001035	0.0163	13	-0.0721	finn-b-G6_SLEEPAPNO	rs61962166	A	G	0.1786	|| id:finn-b-G6_SLEEPAPNO	TRUE	reported	igd	19.56569686
134	20270158	1.83202E-06	0.0232	13	-0.1107	finn-b-G6_SLEEPAPNO	rs1023230	T	C	0.9223	|| id:finn-b-G6_SLEEPAPNO	TRUE	reported	igd	22.76770586
135	33691082	3.83398E-06	0.0166	13	0.0769	finn-b-G6_SLEEPAPNO	rs770267	G	A	0.831	|| id:finn-b-G6_SLEEPAPNO	TRUE	reported	igd	21.46033532
136	71256254	2.46899E-05	0.0434	13	-0.183	finn-b-G6_SLEEPAPNO	rs55633590	A	G	0.02148	|| id:finn-b-G6_SLEEPAPNO	TRUE	reported	igd	17.77963006
137	78954371	4.80795E-05	0.0127	13	0.0515	finn-b-G6_SLEEPAPNO	rs7994008	C	A	0.4149	|| id:finn-b-G6_SLEEPAPNO	TRUE	reported	igd	16.44398289
138	89994898	1.88999E-05	0.0537	14	-0.2299	finn-b-G6_SLEEPAPNO	rs187578918	T	G	0.01472	|| id:finn-b-G6_SLEEPAPNO	TRUE	reported	igd	18.32860328
139	72660200	2.266E-07	0.0143	14	0.0739	finn-b-G6_SLEEPAPNO	rs193546	A	G	0.7438	|| id:finn-b-G6_SLEEPAPNO	TRUE	reported	igd	26.70648931
140	84112949	2.58202E-06	0.0182	14	0.0856	finn-b-G6_SLEEPAPNO	rs1959185	A	G	0.1344	|| id:finn-b-G6_SLEEPAPNO	TRUE	reported	igd	22.12099988
141	29068403	1.08901E-05	0.0125	14	0.0548	finn-b-G6_SLEEPAPNO	rs12890119	T	C	0.4509	|| id:finn-b-G6_SLEEPAPNO	TRUE	reported	igd	19.219456
142	35806769	3.29003E-05	0.0125	14	0.052	finn-b-G6_SLEEPAPNO	rs4982255	A	G	0.5084	|| id:finn-b-G6_SLEEPAPNO	TRUE	reported	igd	17.3056
143	39709405	4.90095E-05	0.0129	14	0.0526	finn-b-G6_SLEEPAPNO	rs17092065	T	C	0.3641	|| id:finn-b-G6_SLEEPAPNO	TRUE	reported	igd	16.62616429
144	62269750	8.78193E-06	0.0501	14	-0.2228	finn-b-G6_SLEEPAPNO	rs149210528	C	T	0.0162	|| id:finn-b-G6_SLEEPAPNO	TRUE	reported	igd	19.77674989
145	52234538	0.00003992	0.0162	14	-0.0667	finn-b-G6_SLEEPAPNO	rs36031408	T	C	0.1821	|| id:finn-b-G6_SLEEPAPNO	TRUE	reported	igd	16.95202713
146	51964865	9.56291E-06	0.0177	15	-0.0783	finn-b-G6_SLEEPAPNO	rs1378892	C	T	0.8563	|| id:finn-b-G6_SLEEPAPNO	TRUE	reported	igd	19.56937662
147	75736354	2.20699E-05	0.0554	15	0.235	finn-b-G6_SLEEPAPNO	rs143989464	G	A	0.01321	|| id:finn-b-G6_SLEEPAPNO	TRUE	reported	igd	17.99352266
148	36607697	4.05695E-05	0.0643	15	0.2637	finn-b-G6_SLEEPAPNO	rs142381302	G	T	0.01024	|| id:finn-b-G6_SLEEPAPNO	TRUE	reported	igd	16.81892809
149	83894350	4.37703E-05	0.0864	16	0.353	finn-b-G6_SLEEPAPNO	rs138193504	C	G	0.005281	|| id:finn-b-G6_SLEEPAPNO	TRUE	reported	igd	16.69252079
150	73312006	0.00002117	0.0127	16	0.0539	finn-b-G6_SLEEPAPNO	rs11149844	T	G	0.5976	|| id:finn-b-G6_SLEEPAPNO	TRUE	reported	igd	18.01233802
151	5008079	4.20998E-05	0.0508	16	0.2082	finn-b-G6_SLEEPAPNO	rs145484783	C	G	0.01524	|| id:finn-b-G6_SLEEPAPNO	TRUE	reported	igd	16.79708909
152	53799507	4.31917E-16	0.0125	16	0.102	finn-b-G6_SLEEPAPNO	rs9937053	A	G	0.4298	|| id:finn-b-G6_SLEEPAPNO	TRUE	reported	igd	66.5856
153	74651733	4.81305E-08	0.0327	16	0.1783	finn-b-G6_SLEEPAPNO	rs142006783	C	T	0.03778	|| id:finn-b-G6_SLEEPAPNO	TRUE	reported	igd	29.73084009
154	52710282	0.00003755	0.0253	16	-0.1041	finn-b-G6_SLEEPAPNO	rs77700051	C	T	0.06588	|| id:finn-b-G6_SLEEPAPNO	TRUE	reported	igd	16.93013482
155	14681005	0.00002715	0.0148	17	-0.0622	finn-b-G6_SLEEPAPNO	rs11655337	C	T	0.2278	|| id:finn-b-G6_SLEEPAPNO	TRUE	reported	igd	17.66271001
156	6783828	4.10696E-05	0.0137	18	0.0562	finn-b-G6_SLEEPAPNO	rs8083583	T	C	0.7029	|| id:finn-b-G6_SLEEPAPNO	TRUE	reported	igd	16.827961
157	24156018	2.08099E-05	0.0129	18	0.0549	finn-b-G6_SLEEPAPNO	rs7240205	T	C	0.6296	|| id:finn-b-G6_SLEEPAPNO	TRUE	reported	igd	18.11195241
158	39496337	3.64998E-05	0.0335	18	-0.1384	finn-b-G6_SLEEPAPNO	rs62083921	A	G	0.03577	|| id:finn-b-G6_SLEEPAPNO	TRUE	reported	igd	17.06799733
159	38872987	3.94403E-06	0.0192	18	-0.0885	finn-b-G6_SLEEPAPNO	rs72892016	A	G	0.1204	|| id:finn-b-G6_SLEEPAPNO	TRUE	reported	igd	21.24633789
160	39148124	4.83905E-05	0.0305	18	0.124	finn-b-G6_SLEEPAPNO	rs1942083	C	G	0.04377	|| id:finn-b-G6_SLEEPAPNO	TRUE	reported	igd	16.52889008
161	33544771	1.06601E-05	0.0404	19	0.1779	finn-b-G6_SLEEPAPNO	rs4805847	C	T	0.9743	|| id:finn-b-G6_SLEEPAPNO	TRUE	reported	igd	19.39050706
162	57676371	5.22998E-06	0.0553	19	0.2517	finn-b-G6_SLEEPAPNO	rs80079566	C	T	0.01352	|| id:finn-b-G6_SLEEPAPNO	TRUE	reported	igd	20.7164897
163	57778255	3.34904E-05	0.0447	19	0.1854	finn-b-G6_SLEEPAPNO	rs75434830	A	T	0.02011	|| id:finn-b-G6_SLEEPAPNO	TRUE	reported	igd	17.20300887
164	7744877	2.25902E-05	0.0205	19	0.0868	finn-b-G6_SLEEPAPNO	rs72996471	A	T	0.1032	|| id:finn-b-G6_SLEEPAPNO	TRUE	reported	igd	17.92799524
165	13786428	1.16899E-05	0.0263	20	-0.1152	finn-b-G6_SLEEPAPNO	rs117670722	T	A	0.06117	|| id:finn-b-G6_SLEEPAPNO	TRUE	reported	igd	19.18639853
166	35062664	3.41201E-05	0.0146	20	0.0606	finn-b-G6_SLEEPAPNO	rs1275392	A	G	0.7595	|| id:finn-b-G6_SLEEPAPNO	TRUE	reported	igd	17.2281854
167	50890662	3.47496E-06	0.0136	20	-0.0632	finn-b-G6_SLEEPAPNO	rs6021831	G	C	0.2973	|| id:finn-b-G6_SLEEPAPNO	TRUE	reported	igd	21.59515571
168	38820805	4.71498E-05	0.0197	20	0.0803	finn-b-G6_SLEEPAPNO	rs6028945	T	G	0.1118	|| id:finn-b-G6_SLEEPAPNO	TRUE	reported	igd	16.61493468
169	25090710	2.54302E-05	0.0134	22	-0.0566	finn-b-G6_SLEEPAPNO	rs5760589	C	G	0.3216	|| id:finn-b-G6_SLEEPAPNO	TRUE	reported	igd	17.8411673

Adjust P-values and LD raw data-T2DM

	chr.exposure	pos.exposure	beta.exposure	se.exposure	pval.exposure	id.exposure	SNP	effect_allele.exposure	other_allele.exposure	eaf.exposure	exposure	mr_keep.exposure	pval_origin.exposure	data_source.exposure	F statistics
1	1	65979280	0.081	0.0192	2.43299E-05	finn-b-E4_DM2_STRICT	rs11208659	C	T	0.08567	|| id:finn-b-E4_DM2_STRICT	TRUE	reported	igd	17.79785156
2	1	39652764	-0.0598	0.0124	0.000001357	finn-b-E4_DM2_STRICT	rs7538300	A	C	0.7413	|| id:finn-b-E4_DM2_STRICT	TRUE	reported	igd	23.25728408
3	1	120418499	0.0646	0.0137	2.48399E-06	finn-b-E4_DM2_STRICT	rs6683791	T	G	0.1933	|| id:finn-b-E4_DM2_STRICT	TRUE	reported	igd	22.23432255
4	1	248166173	-0.2016	0.0484	3.15798E-05	finn-b-E4_DM2_STRICT	rs142728151	A	G	0.01303	|| id:finn-b-E4_DM2_STRICT	TRUE	reported	igd	17.34963459
5	1	203518873	-0.0583	0.0109	9.24209E-08	finn-b-E4_DM2_STRICT	rs13303359	C	A	0.4946	|| id:finn-b-E4_DM2_STRICT	TRUE	reported	igd	28.60777712
6	1	166365665	0.2004	0.0475	2.44901E-05	finn-b-E4_DM2_STRICT	rs77958594	A	G	0.01367	|| id:finn-b-E4_DM2_STRICT	TRUE	reported	igd	17.7995169
7	1	108205163	0.1073	0.0258	3.12903E-05	finn-b-E4_DM2_STRICT	rs72703598	T	C	0.0462	|| id:finn-b-E4_DM2_STRICT	TRUE	reported	igd	17.29657172
8	1	40223128	0.0697	0.0157	9.25295E-06	finn-b-E4_DM2_STRICT	rs3820618	C	T	0.1376	|| id:finn-b-E4_DM2_STRICT	TRUE	reported	igd	19.70907542
9	1	120262152	0.0544	0.0122	8.13598E-06	finn-b-E4_DM2_STRICT	rs12057776	T	C	0.2675	|| id:finn-b-E4_DM2_STRICT	TRUE	reported	igd	19.8828272
10	1	117532790	-0.0613	0.0114	6.70301E-08	finn-b-E4_DM2_STRICT	rs1127215	T	C	0.3494	|| id:finn-b-E4_DM2_STRICT	TRUE	reported	igd	28.91420437
11	1	1923217	-0.0468	0.0115	4.89305E-05	finn-b-E4_DM2_STRICT	rs7529662	G	A	0.3706	|| id:finn-b-E4_DM2_STRICT	TRUE	reported	igd	16.56136106
12	1	214150445	0.2242	0.0412	5.48795E-08	finn-b-E4_DM2_STRICT	rs17712208	A	T	0.01774	|| id:finn-b-E4_DM2_STRICT	TRUE	reported	igd	29.61261665
13	1	214452123	-0.1345	0.0251	8.04192E-08	finn-b-E4_DM2_STRICT	rs72649996	T	G	0.05053	|| id:finn-b-E4_DM2_STRICT	TRUE	reported	igd	28.71422676
14	1	26383125	-0.0563	0.0138	4.49904E-05	finn-b-E4_DM2_STRICT	rs11247852	C	G	0.1907	|| id:finn-b-E4_DM2_STRICT	TRUE	reported	igd	16.64403487
15	1	147313509	0.1187	0.0254	2.95202E-06	finn-b-E4_DM2_STRICT	rs146788179	T	C	0.04774	|| id:finn-b-E4_DM2_STRICT	TRUE	reported	igd	21.83906318
16	1	189347039	0.1471	0.0345	0.00002014	finn-b-E4_DM2_STRICT	rs16830485	C	T	0.02562	|| id:finn-b-E4_DM2_STRICT	TRUE	reported	igd	18.17971855
17	1	5021112	0.0532	0.0108	9.26894E-07	finn-b-E4_DM2_STRICT	rs10753343	T	C	0.4645	|| id:finn-b-E4_DM2_STRICT	TRUE	reported	igd	24.26474623
18	1	20735236	-0.0531	0.0109	1.05801E-06	finn-b-E4_DM2_STRICT	rs1925670	G	T	0.526	|| id:finn-b-E4_DM2_STRICT	TRUE	reported	igd	23.73209326
19	1	229672955	-0.0551	0.0114	0.000001253	finn-b-E4_DM2_STRICT	rs348330	A	G	0.6443	|| id:finn-b-E4_DM2_STRICT	TRUE	reported	igd	23.36111111
20	1	154795389	-0.0575	0.0139	3.57396E-05	finn-b-E4_DM2_STRICT	rs1218598	A	G	0.8133	|| id:finn-b-E4_DM2_STRICT	TRUE	reported	igd	17.11220951
21	1	219671205	0.0476	0.011	1.54401E-05	finn-b-E4_DM2_STRICT	rs11118319	T	C	0.5818	|| id:finn-b-E4_DM2_STRICT	TRUE	reported	igd	18.72528926
22	1	146747153	-0.0586	0.014	2.79898E-05	finn-b-E4_DM2_STRICT	rs6680778	C	T	0.8182	|| id:finn-b-E4_DM2_STRICT	TRUE	reported	igd	17.52020408
23	1	9486622	0.0575	0.0139	3.39899E-05	finn-b-E4_DM2_STRICT	rs67798200	A	C	0.1859	|| id:finn-b-E4_DM2_STRICT	TRUE	reported	igd	17.11220951
24	1	54700707	0.0528	0.0111	0.000002168	finn-b-E4_DM2_STRICT	rs686930	G	A	0.6127	|| id:finn-b-E4_DM2_STRICT	TRUE	reported	igd	22.62673484
25	1	16015986	-0.073	0.0177	3.52201E-05	finn-b-E4_DM2_STRICT	rs12090337	A	C	0.1054	|| id:finn-b-E4_DM2_STRICT	TRUE	reported	igd	17.00979923
26	2	192386522	0.06	0.0148	4.89204E-05	finn-b-E4_DM2_STRICT	rs10432509	C	T	0.1589	|| id:finn-b-E4_DM2_STRICT	TRUE	reported	igd	16.43535427
27	2	198422141	0.197	0.0438	6.86104E-06	finn-b-E4_DM2_STRICT	rs111474510	T	C	0.01633	|| id:finn-b-E4_DM2_STRICT	TRUE	reported	igd	20.22945727
28	2	59316130	-0.0588	0.0111	1.13999E-07	finn-b-E4_DM2_STRICT	rs148465164	A	G	0.3917	|| id:finn-b-E4_DM2_STRICT	TRUE	reported	igd	28.06135866
29	2	128602802	-0.1665	0.0372	7.40491E-06	finn-b-E4_DM2_STRICT	rs77940289	G	A	0.02227	|| id:finn-b-E4_DM2_STRICT	TRUE	reported	igd	20.03284339
30	2	101734925	-0.0571	0.013	0.00001035	finn-b-E4_DM2_STRICT	rs13426934	G	A	0.2256	|| id:finn-b-E4_DM2_STRICT	TRUE	reported	igd	19.29236686
31	2	121347612	-0.0605	0.0126	0.000001689	finn-b-E4_DM2_STRICT	rs11688682	C	G	0.251	|| id:finn-b-E4_DM2_STRICT	TRUE	reported	igd	23.05524061
32	2	1810721	0.1532	0.0353	0.0000138	finn-b-E4_DM2_STRICT	rs79762333	G	T	0.02364	|| id:finn-b-E4_DM2_STRICT	TRUE	reported	igd	18.83510822
33	2	227122216	0.0764	0.0111	6.14469E-12	finn-b-E4_DM2_STRICT	rs1515110	T	G	0.6172	|| id:finn-b-E4_DM2_STRICT	TRUE	reported	igd	47.37407678
34	2	86628744	-0.3562	0.0851	2.82501E-05	finn-b-E4_DM2_STRICT	rs115664987	G	A	0.004186	|| id:finn-b-E4_DM2_STRICT	TRUE	reported	igd	17.51978249
35	2	167246517	0.0681	0.0154	9.90399E-06	finn-b-E4_DM2_STRICT	rs11887519	T	C	0.1441	|| id:finn-b-E4_DM2_STRICT	TRUE	reported	igd	19.55477315
36	2	43252334	-0.0498	0.0117	2.02502E-05	finn-b-E4_DM2_STRICT	rs34347854	G	C	0.3181	|| id:finn-b-E4_DM2_STRICT	TRUE	reported	igd	18.11702827
37	2	230743390	-0.0825	0.0182	6.14101E-06	finn-b-E4_DM2_STRICT	rs143970234	A	G	0.09764	|| id:finn-b-E4_DM2_STRICT	TRUE	reported	igd	20.54779012
38	2	55275560	0.1764	0.0417	2.29699E-05	finn-b-E4_DM2_STRICT	rs115020553	A	G	0.01702	|| id:finn-b-E4_DM2_STRICT	TRUE	reported	igd	17.89472595
39	2	177551886	0.0456	0.0112	4.38904E-05	finn-b-E4_DM2_STRICT	rs1554011	G	A	0.6084	|| id:finn-b-E4_DM2_STRICT	TRUE	reported	igd	16.57653061
40	2	218866486	-0.1263	0.03	2.51102E-05	finn-b-E4_DM2_STRICT	rs76480788	T	C	0.03451	|| id:finn-b-E4_DM2_STRICT	TRUE	reported	igd	17.7241
41	2	9167402	-0.2263	0.0487	0.000003304	finn-b-E4_DM2_STRICT	rs12476966	C	G	0.01346	|| id:finn-b-E4_DM2_STRICT	TRUE	reported	igd	21.59291054
42	2	43480221	0.1402	0.0249	1.79498E-08	finn-b-E4_DM2_STRICT	rs62137406	T	C	0.04888	|| id:finn-b-E4_DM2_STRICT	TRUE	reported	igd	31.70277899
43	2	26982567	0.2727	0.0633	0.00001639	finn-b-E4_DM2_STRICT	rs141791890	T	C	0.007549	|| id:finn-b-E4_DM2_STRICT	TRUE	reported	igd	18.55935401
44	2	165528624	-0.0705	0.0114	6.11406E-10	finn-b-E4_DM2_STRICT	rs1128249	T	G	0.3478	|| id:finn-b-E4_DM2_STRICT	TRUE	reported	igd	38.24445983
45	2	199079789	0.1476	0.0356	3.46402E-05	finn-b-E4_DM2_STRICT	rs140025888	C	T	0.02401	|| id:finn-b-E4_DM2_STRICT	TRUE	reported	igd	17.18987502
46	2	27598097	0.0517	0.0112	3.72598E-06	finn-b-E4_DM2_STRICT	rs4665972	C	T	0.6231	|| id:finn-b-E4_DM2_STRICT	TRUE	reported	igd	21.30811543
47	2	202962022	0.069	0.0169	4.58596E-05	finn-b-E4_DM2_STRICT	rs66578258	G	A	0.1165	|| id:finn-b-E4_DM2_STRICT	TRUE	reported	igd	16.6695844
48	2	220101974	0.1464	0.031	2.32097E-06	finn-b-E4_DM2_STRICT	rs61733623	G	A	0.03172	|| id:finn-b-E4_DM2_STRICT	TRUE	reported	igd	22.30276795
49	2	43453721	-0.1807	0.0306	3.70399E-09	finn-b-E4_DM2_STRICT	rs112694524	A	G	0.03348	|| id:finn-b-E4_DM2_STRICT	TRUE	reported	igd	34.87172669
50	2	60584700	0.0749	0.0162	3.69598E-06	finn-b-E4_DM2_STRICT	rs6545805	G	A	0.1277	|| id:finn-b-E4_DM2_STRICT	TRUE	reported	igd	21.37635269
51	2	99498761	0.2722	0.0668	4.62104E-05	finn-b-E4_DM2_STRICT	rs144635879	A	G	0.006975	|| id:finn-b-E4_DM2_STRICT	TRUE	reported	igd	16.60440496
52	2	69183278	0.3389	0.0777	0.00001295	finn-b-E4_DM2_STRICT	rs78205786	G	C	0.005314	|| id:finn-b-E4_DM2_STRICT	TRUE	reported	igd	19.02396771
53	2	111912729	-0.0542	0.0122	8.87197E-06	finn-b-E4_DM2_STRICT	rs6727356	G	A	0.2796	|| id:finn-b-E4_DM2_STRICT	TRUE	reported	igd	19.73689868
54	3	34654788	-0.071	0.0156	5.13606E-06	finn-b-E4_DM2_STRICT	rs12490975	C	A	0.1404	|| id:finn-b-E4_DM2_STRICT	TRUE	reported	igd	20.71416831
55	3	44920124	-0.0607	0.0138	1.04501E-05	finn-b-E4_DM2_STRICT	rs9683370	A	G	0.1929	|| id:finn-b-E4_DM2_STRICT	TRUE	reported	igd	19.34724848
56	3	172024832	0.0656	0.0156	2.63202E-05	finn-b-E4_DM2_STRICT	rs3733027	T	G	0.141	|| id:finn-b-E4_DM2_STRICT	TRUE	reported	igd	17.68310322
57	3	170629884	0.0733	0.0116	2.87501E-10	finn-b-E4_DM2_STRICT	rs6786846	A	G	0.681	|| id:finn-b-E4_DM2_STRICT	TRUE	reported	igd	39.92932521
58	3	151415928	-0.1499	0.0366	4.15403E-05	finn-b-E4_DM2_STRICT	rs73164654	T	C	0.02285	|| id:finn-b-E4_DM2_STRICT	TRUE	reported	igd	16.77417212
59	3	123124513	-0.0928	0.0139	2.45697E-11	finn-b-E4_DM2_STRICT	rs71330995	A	G	0.1879	|| id:finn-b-E4_DM2_STRICT	TRUE	reported	igd	44.57243414
60	3	12344730	-0.1091	0.0144	3.75837E-14	finn-b-E4_DM2_STRICT	rs11712037	G	C	0.1708	|| id:finn-b-E4_DM2_STRICT	TRUE	reported	igd	57.4016686
61	3	172187534	-0.0513	0.0112	4.51596E-06	finn-b-E4_DM2_STRICT	rs62281225	C	T	0.3821	|| id:finn-b-E4_DM2_STRICT	TRUE	reported	igd	20.97967156
62	3	185503456	0.094	0.0117	9.79716E-16	finn-b-E4_DM2_STRICT	rs6780171	A	T	0.3063	|| id:finn-b-E4_DM2_STRICT	TRUE	reported	igd	64.54817737
63	3	186665645	0.0609	0.0109	2.133E-08	finn-b-E4_DM2_STRICT	rs3887925	T	C	0.4629	|| id:finn-b-E4_DM2_STRICT	TRUE	reported	igd	31.21631176
64	3	23289892	-0.056	0.0135	3.55402E-05	finn-b-E4_DM2_STRICT	rs6792370	T	C	0.1994	|| id:finn-b-E4_DM2_STRICT	TRUE	reported	igd	17.20713306
65	3	23407658	-0.0762	0.0141	7.057E-08	finn-b-E4_DM2_STRICT	rs6550758	C	A	0.8218	|| id:finn-b-E4_DM2_STRICT	TRUE	reported	igd	29.20597555
66	3	105198374	-0.048	0.0116	3.26498E-05	finn-b-E4_DM2_STRICT	rs7431531	C	T	0.324	|| id:finn-b-E4_DM2_STRICT	TRUE	reported	igd	17.12247325
67	3	150213072	0.2029	0.046	1.05201E-05	finn-b-E4_DM2_STRICT	rs141614385	T	C	0.01467	|| id:finn-b-E4_DM2_STRICT	TRUE	reported	igd	19.45577032
68	3	60949804	0.2037	0.039	1.81999E-07	finn-b-E4_DM2_STRICT	rs145908337	T	A	0.02003	|| id:finn-b-E4_DM2_STRICT	TRUE	reported	igd	27.28053254
69	3	48225509	0.055	0.0115	1.85802E-06	finn-b-E4_DM2_STRICT	rs3731503	C	T	0.3299	|| id:finn-b-E4_DM2_STRICT	TRUE	reported	igd	22.87334594
70	3	1074942	-0.4538	0.1051	1.57899E-05	finn-b-E4_DM2_STRICT	rs142351278	T	G	0.002801	|| id:finn-b-E4_DM2_STRICT	TRUE	reported	igd	18.64333275
71	3	152480946	-0.0733	0.0168	0.00001245	finn-b-E4_DM2_STRICT	rs79747397	T	C	0.1197	|| id:finn-b-E4_DM2_STRICT	TRUE	reported	igd	19.03660006
72	3	77671721	-0.0477	0.0114	2.70302E-05	finn-b-E4_DM2_STRICT	rs2272163	A	C	0.3484	|| id:finn-b-E4_DM2_STRICT	TRUE	reported	igd	17.50761773
73	3	63719024	0.2131	0.0523	4.53503E-05	finn-b-E4_DM2_STRICT	rs137986118	A	G	0.01085	|| id:finn-b-E4_DM2_STRICT	TRUE	reported	igd	16.60211897
74	3	137720585	-0.0492	0.0117	2.77102E-05	finn-b-E4_DM2_STRICT	rs57902744	T	G	0.313	|| id:finn-b-E4_DM2_STRICT	TRUE	reported	igd	17.68310322
75	3	138104635	0.0934	0.0174	8.7271E-08	finn-b-E4_DM2_STRICT	rs34905952	A	G	0.1076	|| id:finn-b-E4_DM2_STRICT	TRUE	reported	igd	28.8134496
76	3	16111068	-0.1854	0.0423	1.19201E-05	finn-b-E4_DM2_STRICT	rs79486613	A	G	0.01686	|| id:finn-b-E4_DM2_STRICT	TRUE	reported	igd	19.21050249
77	4	57644286	-0.0748	0.0176	2.19099E-05	finn-b-E4_DM2_STRICT	rs116906920	T	C	0.1104	|| id:finn-b-E4_DM2_STRICT	TRUE	reported	igd	18.0625
78	4	23879277	-0.189	0.0434	1.33101E-05	finn-b-E4_DM2_STRICT	rs115179278	T	C	0.01671	|| id:finn-b-E4_DM2_STRICT	TRUE	reported	igd	18.96462019
79	4	3416880	0.0932	0.0218	1.91298E-05	finn-b-E4_DM2_STRICT	rs77399690	A	G	0.06605	|| id:finn-b-E4_DM2_STRICT	TRUE	reported	igd	18.27758606
80	4	85314016	0.1142	0.0235	0.000001185	finn-b-E4_DM2_STRICT	rs387955	T	C	0.9429	|| id:finn-b-E4_DM2_STRICT	TRUE	reported	igd	23.61546401
81	4	161217268	0.0458	0.0112	4.51804E-05	finn-b-E4_DM2_STRICT	rs56154990	G	A	0.3598	|| id:finn-b-E4_DM2_STRICT	TRUE	reported	igd	16.72225765
82	4	96152313	0.0573	0.0109	1.38599E-07	finn-b-E4_DM2_STRICT	rs2865383	C	T	0.445	|| id:finn-b-E4_DM2_STRICT	TRUE	reported	igd	27.63479505
83	4	103679117	-0.0523	0.0111	2.42399E-06	finn-b-E4_DM2_STRICT	rs223490	T	G	0.3913	|| id:finn-b-E4_DM2_STRICT	TRUE	reported	igd	22.20022725
84	4	54521821	0.143	0.0283	4.26904E-07	finn-b-E4_DM2_STRICT	rs77563431	A	C	0.03886	|| id:finn-b-E4_DM2_STRICT	TRUE	reported	igd	25.53284471
85	4	6304242	0.0842	0.0111	3.96004E-14	finn-b-E4_DM2_STRICT	rs1046317	C	T	0.6105	|| id:finn-b-E4_DM2_STRICT	TRUE	reported	igd	57.54110868
86	4	11942411	-0.5506	0.1235	8.18295E-06	finn-b-E4_DM2_STRICT	rs75196907	G	C	0.001969	|| id:finn-b-E4_DM2_STRICT	TRUE	reported	igd	19.87643528
87	4	25408838	-0.0662	0.013	3.18999E-07	finn-b-E4_DM2_STRICT	rs34811474	A	G	0.2299	|| id:finn-b-E4_DM2_STRICT	TRUE	reported	igd	25.93159763
88	4	119650768	0.0813	0.0194	2.89101E-05	finn-b-E4_DM2_STRICT	rs116030177	C	T	0.08405	|| id:finn-b-E4_DM2_STRICT	TRUE	reported	igd	17.56214794
89	4	117184812	0.1305	0.0322	4.93299E-05	finn-b-E4_DM2_STRICT	rs114398530	A	G	0.02979	|| id:finn-b-E4_DM2_STRICT	TRUE	reported	igd	16.42514756
90	4	37256513	0.0515	0.0109	2.36701E-06	finn-b-E4_DM2_STRICT	rs1865537	C	A	0.5034	|| id:finn-b-E4_DM2_STRICT	TRUE	reported	igd	22.32345762
91	4	185360041	-0.0577	0.0135	1.84901E-05	finn-b-E4_DM2_STRICT	rs3775566	T	C	0.2272	|| id:finn-b-E4_DM2_STRICT	TRUE	reported	igd	18.26770919
92	4	185718132	-0.083	0.0152	4.64098E-08	finn-b-E4_DM2_STRICT	rs745805	T	A	0.1503	|| id:finn-b-E4_DM2_STRICT	TRUE	reported	igd	29.81734765
93	4	165725642	-0.0666	0.0157	2.12701E-05	finn-b-E4_DM2_STRICT	rs17486393	A	C	0.1406	|| id:finn-b-E4_DM2_STRICT	TRUE	reported	igd	17.99488823
94	4	63338612	0.2264	0.0508	8.40601E-06	finn-b-E4_DM2_STRICT	rs140296079	C	T	0.01161	|| id:finn-b-E4_DM2_STRICT	TRUE	reported	igd	19.86211172
95	4	45182527	0.0742	0.0108	7.51796E-12	finn-b-E4_DM2_STRICT	rs10938397	G	A	0.4736	|| id:finn-b-E4_DM2_STRICT	TRUE	reported	igd	47.20198903
96	4	106718253	0.0809	0.0185	1.23001E-05	finn-b-E4_DM2_STRICT	rs72673821	A	G	0.09712	|| id:finn-b-E4_DM2_STRICT	TRUE	reported	igd	19.12289262
97	4	153518990	-0.0456	0.0109	3.05598E-05	finn-b-E4_DM2_STRICT	rs4696138	G	T	0.4474	|| id:finn-b-E4_DM2_STRICT	TRUE	reported	igd	17.50155711
98	4	143675717	0.0464	0.0112	3.61701E-05	finn-b-E4_DM2_STRICT	rs331966	C	A	0.3654	|| id:finn-b-E4_DM2_STRICT	TRUE	reported	igd	17.16326531
99	4	81981434	0.0488	0.0109	7.48893E-06	finn-b-E4_DM2_STRICT	rs4693810	T	C	0.46	|| id:finn-b-E4_DM2_STRICT	TRUE	reported	igd	20.04410403
100	5	2627952	0.1065	0.0256	3.23899E-05	finn-b-E4_DM2_STRICT	rs116593349	T	C	0.05088	|| id:finn-b-E4_DM2_STRICT	TRUE	reported	igd	17.30690002
101	5	44708378	0.0482	0.0111	1.33801E-05	finn-b-E4_DM2_STRICT	rs6861560	G	C	0.4019	|| id:finn-b-E4_DM2_STRICT	TRUE	reported	igd	18.85593702
102	5	102143311	0.1386	0.0232	2.28802E-09	finn-b-E4_DM2_STRICT	rs76177300	A	G	0.05776	|| id:finn-b-E4_DM2_STRICT	TRUE	reported	igd	35.69032402
103	5	55884741	0.1875	0.0447	2.77498E-05	finn-b-E4_DM2_STRICT	rs6897971	G	T	0.01538	|| id:finn-b-E4_DM2_STRICT	TRUE	reported	igd	17.59492816
104	5	57383449	0.2055	0.0486	2.30999E-05	finn-b-E4_DM2_STRICT	rs7717056	A	G	0.01246	|| id:finn-b-E4_DM2_STRICT	TRUE	reported	igd	17.8793248
105	5	39047251	-0.1298	0.0319	4.68803E-05	finn-b-E4_DM2_STRICT	rs75681807	C	G	0.03064	|| id:finn-b-E4_DM2_STRICT	TRUE	reported	igd	16.55648038
106	5	14845667	-0.0496	0.011	6.04603E-06	finn-b-E4_DM2_STRICT	rs826356	C	G	0.4207	|| id:finn-b-E4_DM2_STRICT	TRUE	reported	igd	20.33190083
107	5	141760416	0.0554	0.0121	4.32603E-06	finn-b-E4_DM2_STRICT	rs1209696	C	T	0.7081	|| id:finn-b-E4_DM2_STRICT	TRUE	reported	igd	20.96277577
108	5	150464590	0.1227	0.0274	7.69006E-06	finn-b-E4_DM2_STRICT	rs71586196	A	C	0.04077	|| id:finn-b-E4_DM2_STRICT	TRUE	reported	igd	20.05339922
109	5	74919409	-0.0524	0.0127	3.62602E-05	finn-b-E4_DM2_STRICT	rs6861279	A	G	0.2396	|| id:finn-b-E4_DM2_STRICT	TRUE	reported	igd	17.02374605
110	5	76435004	-0.0671	0.0129	1.92398E-07	finn-b-E4_DM2_STRICT	rs7732130	A	G	0.7725	|| id:finn-b-E4_DM2_STRICT	TRUE	reported	igd	27.05612643
111	5	133858719	0.0499	0.0114	1.11599E-05	finn-b-E4_DM2_STRICT	rs329115	C	T	0.3482	|| id:finn-b-E4_DM2_STRICT	TRUE	reported	igd	19.15981841
112	5	167389006	-0.0588	0.0137	1.84302E-05	finn-b-E4_DM2_STRICT	rs6865920	T	A	0.1946	|| id:finn-b-E4_DM2_STRICT	TRUE	reported	igd	18.42101337
113	5	116636706	-0.1318	0.0301	1.20501E-05	finn-b-E4_DM2_STRICT	rs62373955	G	A	0.03469	|| id:finn-b-E4_DM2_STRICT	TRUE	reported	igd	19.17334246
114	5	15980162	0.1451	0.0334	0.0000141	finn-b-E4_DM2_STRICT	rs78233867	A	T	0.02782	|| id:finn-b-E4_DM2_STRICT	TRUE	reported	igd	18.87304134
115	6	32120715	0.0567	0.0126	7.13608E-06	finn-b-E4_DM2_STRICT	rs1053924	C	T	0.6344	|| id:finn-b-E4_DM2_STRICT	TRUE	reported	igd	20.25
116	6	129802971	-0.0588	0.0132	8.28495E-06	finn-b-E4_DM2_STRICT	rs7775030	G	A	0.2181	|| id:finn-b-E4_DM2_STRICT	TRUE	reported	igd	19.84297521
117	6	7232186	0.064	0.011	5.11705E-09	finn-b-E4_DM2_STRICT	rs9505086	C	T	0.4439	|| id:finn-b-E4_DM2_STRICT	TRUE	reported	igd	33.85123967
118	6	127068983	-0.0601	0.0122	7.57007E-07	finn-b-E4_DM2_STRICT	rs853974	C	T	0.7319	|| id:finn-b-E4_DM2_STRICT	TRUE	reported	igd	24.26773717
119	6	43757082	0.0549	0.0109	4.91202E-07	finn-b-E4_DM2_STRICT	rs4711750	A	T	0.4979	|| id:finn-b-E4_DM2_STRICT	TRUE	reported	igd	25.36831917
120	6	31170228	0.0567	0.0119	1.75001E-06	finn-b-E4_DM2_STRICT	rs9366770	C	G	0.5623	|| id:finn-b-E4_DM2_STRICT	TRUE	reported	igd	22.70242215
121	6	88557531	0.1438	0.033	0.00001309	finn-b-E4_DM2_STRICT	rs17490984	G	A	0.02857	|| id:finn-b-E4_DM2_STRICT	TRUE	reported	igd	18.98846648
122	6	153383590	0.0542	0.0122	9.07403E-06	finn-b-E4_DM2_STRICT	rs9397581	A	G	0.2667	|| id:finn-b-E4_DM2_STRICT	TRUE	reported	igd	19.73689868
123	6	140628309	-0.0525	0.0126	3.24302E-05	finn-b-E4_DM2_STRICT	rs4429962	A	G	0.2419	|| id:finn-b-E4_DM2_STRICT	TRUE	reported	igd	17.36111111
124	6	20673880	0.128	0.0115	6.30957E-29	finn-b-E4_DM2_STRICT	rs7451008	C	T	0.3292	|| id:finn-b-E4_DM2_STRICT	TRUE	reported	igd	123.8865784
125	6	131881146	0.0766	0.013	4.25598E-09	finn-b-E4_DM2_STRICT	rs2781655	T	C	0.2218	|| id:finn-b-E4_DM2_STRICT	TRUE	reported	igd	34.71928994
126	6	100105028	0.0446	0.0109	4.49604E-05	finn-b-E4_DM2_STRICT	rs7758796	A	G	0.4264	|| id:finn-b-E4_DM2_STRICT	TRUE	reported	igd	16.74236175
127	6	19821910	-0.0972	0.0195	6.04199E-07	finn-b-E4_DM2_STRICT	rs7747295	G	C	0.0845	|| id:finn-b-E4_DM2_STRICT	TRUE	reported	igd	24.84639053
128	6	48323576	0.0458	0.0112	4.30903E-05	finn-b-E4_DM2_STRICT	rs325033	C	A	0.6195	|| id:finn-b-E4_DM2_STRICT	TRUE	reported	igd	16.72225765
129	6	139824992	0.0556	0.0108	2.81702E-07	finn-b-E4_DM2_STRICT	rs590015	A	G	0.5031	|| id:finn-b-E4_DM2_STRICT	TRUE	reported	igd	26.50342936
130	7	31362485	-0.0545	0.0127	1.80401E-05	finn-b-E4_DM2_STRICT	rs10237360	G	A	0.24	|| id:finn-b-E4_DM2_STRICT	TRUE	reported	igd	18.41558683
131	7	76517055	-0.0506	0.0111	5.39697E-06	finn-b-E4_DM2_STRICT	rs11981565	T	C	0.3896	|| id:finn-b-E4_DM2_STRICT	TRUE	reported	igd	20.78045613
132	7	103560616	-0.0475	0.011	0.00001532	finn-b-E4_DM2_STRICT	rs802788	G	A	0.4123	|| id:finn-b-E4_DM2_STRICT	TRUE	reported	igd	18.64669421
133	7	119607248	0.3564	0.0811	0.00001102	finn-b-E4_DM2_STRICT	rs191721892	G	A	0.004919	|| id:finn-b-E4_DM2_STRICT	TRUE	reported	igd	19.31228591
134	7	131533816	-0.0531	0.0131	4.83704E-05	finn-b-E4_DM2_STRICT	rs2333896	A	G	0.7808	|| id:finn-b-E4_DM2_STRICT	TRUE	reported	igd	16.43033623
135	7	43411033	0.0492	0.0111	9.72098E-06	finn-b-E4_DM2_STRICT	rs10273324	A	G	0.3836	|| id:finn-b-E4_DM2_STRICT	TRUE	reported	igd	19.64645727
136	7	15065564	0.0556	0.0109	3.38704E-07	finn-b-E4_DM2_STRICT	rs1558317	A	T	0.4561	|| id:finn-b-E4_DM2_STRICT	TRUE	reported	igd	26.01935864
137	7	16939416	-0.2151	0.0409	1.46801E-07	finn-b-E4_DM2_STRICT	rs58135780	G	T	0.01871	|| id:finn-b-E4_DM2_STRICT	TRUE	reported	igd	27.65885546
138	7	102086605	0.0996	0.014	1.21088E-12	finn-b-E4_DM2_STRICT	rs57307671	T	A	0.1834	|| id:finn-b-E4_DM2_STRICT	TRUE	reported	igd	50.61306122
139	7	102341967	-0.1022	0.0208	9.02693E-07	finn-b-E4_DM2_STRICT	rs73714509	C	T	0.07342	|| id:finn-b-E4_DM2_STRICT	TRUE	reported	igd	24.14210429
140	7	150537635	0.0768	0.0114	1.84204E-11	finn-b-E4_DM2_STRICT	rs62492368	A	G	0.3401	|| id:finn-b-E4_DM2_STRICT	TRUE	reported	igd	45.38504155
141	7	156826585	-0.1245	0.0257	1.25701E-06	finn-b-E4_DM2_STRICT	rs13244987	A	G	0.04703	|| id:finn-b-E4_DM2_STRICT	TRUE	reported	igd	23.46780421
142	7	130466854	0.0585	0.0109	6.97108E-08	finn-b-E4_DM2_STRICT	rs972283	G	A	0.543	|| id:finn-b-E4_DM2_STRICT	TRUE	reported	igd	28.80439357
143	7	101513404	0.0487	0.011	9.35793E-06	finn-b-E4_DM2_STRICT	rs10245008	C	T	0.4047	|| id:finn-b-E4_DM2_STRICT	TRUE	reported	igd	19.6007438
144	7	78478887	0.1472	0.034	1.50699E-05	finn-b-E4_DM2_STRICT	rs117668655	C	G	0.02655	|| id:finn-b-E4_DM2_STRICT	TRUE	reported	igd	18.74380623
145	7	51087799	-0.0645	0.0142	5.35501E-06	finn-b-E4_DM2_STRICT	rs735048	G	A	0.8248	|| id:finn-b-E4_DM2_STRICT	TRUE	reported	igd	20.63206705
146	7	146819613	-0.2365	0.0537	1.05801E-05	finn-b-E4_DM2_STRICT	rs117211351	T	C	0.0106	|| id:finn-b-E4_DM2_STRICT	TRUE	reported	igd	19.39606893
147	7	84019825	0.2165	0.0494	1.18201E-05	finn-b-E4_DM2_STRICT	rs75565674	T	C	0.01244	|| id:finn-b-E4_DM2_STRICT	TRUE	reported	igd	19.20710469
148	7	1913270	-0.0563	0.0126	8.67801E-06	finn-b-E4_DM2_STRICT	rs11973114	A	G	0.2415	|| id:finn-b-E4_DM2_STRICT	TRUE	reported	igd	19.96529352
149	7	28142186	0.0666	0.0115	6.54696E-09	finn-b-E4_DM2_STRICT	rs10245867	T	G	0.3307	|| id:finn-b-E4_DM2_STRICT	TRUE	reported	igd	33.53920605
150	7	14898282	0.0587	0.0136	0.00001509	finn-b-E4_DM2_STRICT	rs17168486	T	C	0.198	|| id:finn-b-E4_DM2_STRICT	TRUE	reported	igd	18.62937933
151	7	44255643	0.0894	0.0133	2.05305E-11	finn-b-E4_DM2_STRICT	rs878521	A	G	0.2072	|| id:finn-b-E4_DM2_STRICT	TRUE	reported	igd	45.18265589
152	7	74120370	0.0702	0.0152	3.77598E-06	finn-b-E4_DM2_STRICT	rs34997674	A	G	0.1506	|| id:finn-b-E4_DM2_STRICT	TRUE	reported	igd	21.32981302
153	7	99550801	-0.0607	0.0141	1.71498E-05	finn-b-E4_DM2_STRICT	rs10953292	G	C	0.1801	|| id:finn-b-E4_DM2_STRICT	TRUE	reported	igd	18.53271968
154	7	129838400	-0.0986	0.0233	0.00002256	finn-b-E4_DM2_STRICT	rs138275353	A	G	0.05866	|| id:finn-b-E4_DM2_STRICT	TRUE	reported	igd	17.90778979
155	7	157420736	-0.0508	0.011	4.07897E-06	finn-b-E4_DM2_STRICT	rs887004	T	C	0.4226	|| id:finn-b-E4_DM2_STRICT	TRUE	reported	igd	21.32760331
156	8	95934907	0.0462	0.0111	3.00898E-05	finn-b-E4_DM2_STRICT	rs2515236	G	A	0.3892	|| id:finn-b-E4_DM2_STRICT	TRUE	reported	igd	17.32359386
157	8	19612052	-0.056	0.0119	2.37602E-06	finn-b-E4_DM2_STRICT	rs7816239	C	T	0.3009	|| id:finn-b-E4_DM2_STRICT	TRUE	reported	igd	22.14532872
158	8	36042550	0.1024	0.0223	4.49697E-06	finn-b-E4_DM2_STRICT	rs78298566	C	A	0.06242	|| id:finn-b-E4_DM2_STRICT	TRUE	reported	igd	21.08580506
159	8	41513531	0.0579	0.0109	9.52994E-08	finn-b-E4_DM2_STRICT	rs17659386	C	T	0.5476	|| id:finn-b-E4_DM2_STRICT	TRUE	reported	igd	28.21656426
160	8	87617188	0.05	0.0114	1.13899E-05	finn-b-E4_DM2_STRICT	rs9297935	C	T	0.3412	|| id:finn-b-E4_DM2_STRICT	TRUE	reported	igd	19.23668821
161	8	80854649	0.1088	0.0267	0.00004492	finn-b-E4_DM2_STRICT	rs111608525	C	T	0.04323	|| id:finn-b-E4_DM2_STRICT	TRUE	reported	igd	16.6048619
162	8	135356050	0.0609	0.0121	4.49397E-07	finn-b-E4_DM2_STRICT	rs11775271	T	C	0.2848	|| id:finn-b-E4_DM2_STRICT	TRUE	reported	igd	25.33167133
163	8	146181151	-0.1155	0.0245	2.39398E-06	finn-b-E4_DM2_STRICT	rs144085411	T	C	0.05402	|| id:finn-b-E4_DM2_STRICT	TRUE	reported	igd	22.2244898
164	8	118185733	-0.0814	0.0112	2.97098E-13	finn-b-E4_DM2_STRICT	rs11558471	G	A	0.3785	|| id:finn-b-E4_DM2_STRICT	TRUE	reported	igd	52.82174745
165	8	77575664	0.0533	0.0123	1.53699E-05	finn-b-E4_DM2_STRICT	rs1433954	G	A	0.2618	|| id:finn-b-E4_DM2_STRICT	TRUE	reported	igd	18.77777778
166	8	1680907	-0.1873	0.0451	3.34904E-05	finn-b-E4_DM2_STRICT	rs142536106	C	A	0.01489	|| id:finn-b-E4_DM2_STRICT	TRUE	reported	igd	17.24735375
167	9	96910815	0.0576	0.0132	0.00001268	finn-b-E4_DM2_STRICT	rs10821304	A	G	0.2139	|| id:finn-b-E4_DM2_STRICT	TRUE	reported	igd	19.04132231
168	9	124272810	-0.1675	0.0358	0.000002929	finn-b-E4_DM2_STRICT	rs113207182	T	C	0.02373	|| id:finn-b-E4_DM2_STRICT	TRUE	reported	igd	21.8908976
169	9	136154867	0.0619	0.0129	1.52198E-06	finn-b-E4_DM2_STRICT	rs495828	T	G	0.2233	|| id:finn-b-E4_DM2_STRICT	TRUE	reported	igd	23.02511868
170	9	4134978	0.1066	0.02	9.47E-08	finn-b-E4_DM2_STRICT	rs78993812	A	C	0.08079	|| id:finn-b-E4_DM2_STRICT	TRUE	reported	igd	28.4089
171	9	4291928	0.0597	0.0112	8.95695E-08	finn-b-E4_DM2_STRICT	rs10974438	C	A	0.3756	|| id:finn-b-E4_DM2_STRICT	TRUE	reported	igd	28.41270727
172	9	139936141	-0.0869	0.0207	2.65797E-05	finn-b-E4_DM2_STRICT	rs13289619	A	G	0.07517	|| id:finn-b-E4_DM2_STRICT	TRUE	reported	igd	17.62377185
173	9	36148968	-0.479	0.1041	4.17398E-06	finn-b-E4_DM2_STRICT	rs118007921	A	C	0.002999	|| id:finn-b-E4_DM2_STRICT	TRUE	reported	igd	21.17237453
174	9	35132441	0.0646	0.0129	6.05397E-07	finn-b-E4_DM2_STRICT	rs10814198	A	G	0.7752	|| id:finn-b-E4_DM2_STRICT	TRUE	reported	igd	25.07757947
175	9	77187780	-0.0775	0.0181	1.86801E-05	finn-b-E4_DM2_STRICT	rs17612183	T	C	0.1006	|| id:finn-b-E4_DM2_STRICT	TRUE	reported	igd	18.33353683
176	9	22132729	-0.125	0.0152	1.6151E-16	finn-b-E4_DM2_STRICT	rs10965247	G	A	0.1519	|| id:finn-b-E4_DM2_STRICT	TRUE	reported	igd	67.62898199
177	9	22137685	0.1144	0.0121	3.17395E-21	finn-b-E4_DM2_STRICT	rs7018475	G	T	0.2788	|| id:finn-b-E4_DM2_STRICT	TRUE	reported	igd	89.38842975
178	9	139248082	0.1004	0.0118	1.5481E-17	finn-b-E4_DM2_STRICT	rs28642213	G	A	0.6971	|| id:finn-b-E4_DM2_STRICT	TRUE	reported	igd	72.39413962
179	9	95723503	0.1422	0.0331	1.73002E-05	finn-b-E4_DM2_STRICT	rs74581767	A	G	0.02745	|| id:finn-b-E4_DM2_STRICT	TRUE	reported	igd	18.45623899
180	9	2622134	-0.0499	0.0114	1.18299E-05	finn-b-E4_DM2_STRICT	rs34881325	T	C	0.3744	|| id:finn-b-E4_DM2_STRICT	TRUE	reported	igd	19.15981841
181	9	103797594	0.0508	0.0112	5.23504E-06	finn-b-E4_DM2_STRICT	rs2480736	A	G	0.6127	|| id:finn-b-E4_DM2_STRICT	TRUE	reported	igd	20.57270408
182	9	117190330	-0.0518	0.012	0.00001495	finn-b-E4_DM2_STRICT	rs4979388	G	A	0.2901	|| id:finn-b-E4_DM2_STRICT	TRUE	reported	igd	18.63361111
183	9	19101350	0.0561	0.0132	2.19498E-05	finn-b-E4_DM2_STRICT	rs12003940	G	A	0.212	|| id:finn-b-E4_DM2_STRICT	TRUE	reported	igd	18.0625
184	10	71335802	-0.0611	0.0122	0.000000518	finn-b-E4_DM2_STRICT	rs12769661	A	G	0.277	|| id:finn-b-E4_DM2_STRICT	TRUE	reported	igd	25.0820344
185	10	12309269	0.0854	0.0123	4.22863E-12	finn-b-E4_DM2_STRICT	rs11257659	T	C	0.2647	|| id:finn-b-E4_DM2_STRICT	TRUE	reported	igd	48.20649085
186	10	77096354	-0.0591	0.0123	1.60299E-06	finn-b-E4_DM2_STRICT	rs2488707	T	C	0.2635	|| id:finn-b-E4_DM2_STRICT	TRUE	reported	igd	23.08685306
187	10	102758344	-0.1707	0.0391	0.00001238	finn-b-E4_DM2_STRICT	rs144167520	A	G	0.0199	|| id:finn-b-E4_DM2_STRICT	TRUE	reported	igd	19.05958883
188	10	54928586	0.0463	0.0111	2.91602E-05	finn-b-E4_DM2_STRICT	rs12763047	A	G	0.3871	|| id:finn-b-E4_DM2_STRICT	TRUE	reported	igd	17.39866894
189	10	88030441	0.0466	0.0109	0.00001907	finn-b-E4_DM2_STRICT	rs10887571	T	C	0.4499	|| id:finn-b-E4_DM2_STRICT	TRUE	reported	igd	18.27758606
190	10	33573150	-0.2362	0.058	4.67197E-05	finn-b-E4_DM2_STRICT	rs150206691	G	T	0.009371	|| id:finn-b-E4_DM2_STRICT	TRUE	reported	igd	16.5845541
191	10	68463784	0.0694	0.0136	3.661E-07	finn-b-E4_DM2_STRICT	rs4745916	A	T	0.1954	|| id:finn-b-E4_DM2_STRICT	TRUE	reported	igd	26.04000865
192	10	114737633	-0.2166	0.036	1.754E-09	finn-b-E4_DM2_STRICT	rs144155527	T	C	0.0236	|| id:finn-b-E4_DM2_STRICT	TRUE	reported	igd	36.20027778
193	10	135235890	0.0579	0.0135	0.00001859	finn-b-E4_DM2_STRICT	rs1056521	C	T	0.2002	|| id:finn-b-E4_DM2_STRICT	TRUE	reported	igd	18.3945679
194	10	114758349	0.3055	0.0138	3.0339E-109	finn-b-E4_DM2_STRICT	rs7903146	T	C	0.1992	|| id:finn-b-E4_DM2_STRICT	TRUE	reported	igd	490.0769271
195	10	120779605	-0.2316	0.0449	2.46099E-07	finn-b-E4_DM2_STRICT	rs371034348	G	C	0.01594	|| id:finn-b-E4_DM2_STRICT	TRUE	reported	igd	26.6062966
196	10	121708929	0.0601	0.0132	5.16202E-06	finn-b-E4_DM2_STRICT	rs12571664	C	T	0.2149	|| id:finn-b-E4_DM2_STRICT	TRUE	reported	igd	20.73008494
197	10	133841039	-0.0522	0.0112	3.26701E-06	finn-b-E4_DM2_STRICT	rs2026781	C	G	0.5838	|| id:finn-b-E4_DM2_STRICT	TRUE	reported	igd	21.72225765
198	10	71449878	0.1576	0.0281	1.963E-08	finn-b-E4_DM2_STRICT	rs182788819	T	C	0.03871	|| id:finn-b-E4_DM2_STRICT	TRUE	reported	igd	31.45573131
199	10	114912965	0.1453	0.0299	1.17401E-06	finn-b-E4_DM2_STRICT	rs61872821	G	A	0.03575	|| id:finn-b-E4_DM2_STRICT	TRUE	reported	igd	23.61504905
200	10	45606421	-0.1433	0.0346	3.52201E-05	finn-b-E4_DM2_STRICT	rs146658417	A	G	0.02652	|| id:finn-b-E4_DM2_STRICT	TRUE	reported	igd	17.15300378
201	10	96517572	-0.0949	0.0225	0.00002381	finn-b-E4_DM2_STRICT	rs61886219	T	C	0.0636	|| id:finn-b-E4_DM2_STRICT	TRUE	reported	igd	17.78964938
202	10	94460650	-0.0788	0.0108	3.41271E-13	finn-b-E4_DM2_STRICT	rs10882099	C	T	0.4772	|| id:finn-b-E4_DM2_STRICT	TRUE	reported	igd	53.23593964
203	11	983626	0.1577	0.0296	9.88895E-08	finn-b-E4_DM2_STRICT	rs78721871	A	G	0.03554	|| id:finn-b-E4_DM2_STRICT	TRUE	reported	igd	28.38441609
204	11	28060943	0.058	0.0126	4.37301E-06	finn-b-E4_DM2_STRICT	rs58502944	A	C	0.2495	|| id:finn-b-E4_DM2_STRICT	TRUE	reported	igd	21.18921643
205	11	32520509	0.0682	0.0139	9.84895E-07	finn-b-E4_DM2_STRICT	rs10835932	T	C	0.1845	|| id:finn-b-E4_DM2_STRICT	TRUE	reported	igd	24.07349516
206	11	65485218	0.0502	0.012	2.98497E-05	finn-b-E4_DM2_STRICT	rs3372	A	G	0.2854	|| id:finn-b-E4_DM2_STRICT	TRUE	reported	igd	17.50027778
207	11	3875411	-0.0497	0.0118	2.56898E-05	finn-b-E4_DM2_STRICT	rs4910858	G	A	0.3039	|| id:finn-b-E4_DM2_STRICT	TRUE	reported	igd	17.73980178
208	11	127993631	-0.1559	0.0339	4.35001E-06	finn-b-E4_DM2_STRICT	rs79606992	G	A	0.02718	|| id:finn-b-E4_DM2_STRICT	TRUE	reported	igd	21.14914594
209	11	60525462	-0.1909	0.0399	1.69699E-06	finn-b-E4_DM2_STRICT	rs61898833	C	A	0.02089	|| id:finn-b-E4_DM2_STRICT	TRUE	reported	igd	22.89106852
210	11	72463435	-0.094	0.0128	1.81092E-13	finn-b-E4_DM2_STRICT	rs7109575	A	G	0.2376	|| id:finn-b-E4_DM2_STRICT	TRUE	reported	igd	53.93066406
211	11	2729947	0.0786	0.0117	1.68694E-11	finn-b-E4_DM2_STRICT	rs7925578	T	G	0.3213	|| id:finn-b-E4_DM2_STRICT	TRUE	reported	igd	45.13083498
212	11	113838928	0.0894	0.0212	0.00002398	finn-b-E4_DM2_STRICT	rs76168892	T	C	0.07165	|| id:finn-b-E4_DM2_STRICT	TRUE	reported	igd	17.78292987
213	11	133677074	0.0536	0.0128	2.98696E-05	finn-b-E4_DM2_STRICT	rs77882697	T	C	0.2397	|| id:finn-b-E4_DM2_STRICT	TRUE	reported	igd	17.53515625
214	11	78729723	0.3746	0.0857	1.24701E-05	finn-b-E4_DM2_STRICT	rs34843570	T	C	0.004206	|| id:finn-b-E4_DM2_STRICT	TRUE	reported	igd	19.10618164
215	11	92708710	0.1316	0.0113	2.34909E-31	finn-b-E4_DM2_STRICT	rs10830963	G	C	0.3567	|| id:finn-b-E4_DM2_STRICT	TRUE	reported	igd	135.6297282
216	11	93255014	0.0543	0.0108	5.36402E-07	finn-b-E4_DM2_STRICT	rs897558	G	A	0.4766	|| id:finn-b-E4_DM2_STRICT	TRUE	reported	igd	25.27854938
217	11	2184848	-0.0628	0.0126	6.91194E-07	finn-b-E4_DM2_STRICT	rs3842727	T	G	0.7521	|| id:finn-b-E4_DM2_STRICT	TRUE	reported	igd	24.84152179
218	11	17408630	-0.0619	0.0108	1.11501E-08	finn-b-E4_DM2_STRICT	rs5215	T	C	0.5286	|| id:finn-b-E4_DM2_STRICT	TRUE	reported	igd	32.84987997
219	11	8520890	-0.0623	0.0146	0.00001902	finn-b-E4_DM2_STRICT	rs143681192	C	G	0.1666	|| id:finn-b-E4_DM2_STRICT	TRUE	reported	igd	18.20834115
220	11	61893741	-0.0494	0.012	4.05005E-05	finn-b-E4_DM2_STRICT	rs4963464	T	C	0.2857	|| id:finn-b-E4_DM2_STRICT	TRUE	reported	igd	16.94694444
221	11	2858546	-0.1982	0.0201	6.75927E-23	finn-b-E4_DM2_STRICT	rs2237897	T	C	0.08138	|| id:finn-b-E4_DM2_STRICT	TRUE	reported	igd	97.23333581
222	11	113426669	-0.3803	0.0828	4.40099E-06	finn-b-E4_DM2_STRICT	rs78562438	T	C	0.004903	|| id:finn-b-E4_DM2_STRICT	TRUE	reported	igd	21.09560462
223	11	116376584	0.1189	0.0274	1.40501E-05	finn-b-E4_DM2_STRICT	rs77368797	T	G	0.04318	|| id:finn-b-E4_DM2_STRICT	TRUE	reported	igd	18.83053173
224	12	4384844	-0.5069	0.0339	1.51496E-50	finn-b-E4_DM2_STRICT	rs76895963	G	T	0.03124	|| id:finn-b-E4_DM2_STRICT	TRUE	reported	igd	223.5862984
225	12	4365572	-0.258	0.0405	1.852E-10	finn-b-E4_DM2_STRICT	rs74862545	T	C	0.01953	|| id:finn-b-E4_DM2_STRICT	TRUE	reported	igd	40.58161866
226	12	4883540	-0.1065	0.0248	1.72898E-05	finn-b-E4_DM2_STRICT	rs80312557	T	A	0.05124	|| id:finn-b-E4_DM2_STRICT	TRUE	reported	igd	18.44148348
227	12	66179054	0.2548	0.0359	1.21507E-12	finn-b-E4_DM2_STRICT	rs73113806	T	C	0.02331	|| id:finn-b-E4_DM2_STRICT	TRUE	reported	igd	50.3744074
228	12	4671429	-0.0575	0.0115	5.66304E-07	finn-b-E4_DM2_STRICT	rs34206253	T	G	0.344	|| id:finn-b-E4_DM2_STRICT	TRUE	reported	igd	25
229	12	30149533	0.4259	0.1025	3.24302E-05	finn-b-E4_DM2_STRICT	rs12367836	G	C	0.002855	|| id:finn-b-E4_DM2_STRICT	TRUE	reported	igd	17.26503843
230	12	4271088	-0.3709	0.038	1.74502E-22	finn-b-E4_DM2_STRICT	rs112108223	A	G	0.02242	|| id:finn-b-E4_DM2_STRICT	TRUE	reported	igd	95.26787396
231	12	4372100	0.133	0.031	1.82301E-05	finn-b-E4_DM2_STRICT	rs117380042	T	C	0.03168	|| id:finn-b-E4_DM2_STRICT	TRUE	reported	igd	18.40686785
232	12	32690857	0.0569	0.0118	1.27101E-06	finn-b-E4_DM2_STRICT	rs4931017	G	A	0.6923	|| id:finn-b-E4_DM2_STRICT	TRUE	reported	igd	23.25201092
233	12	50248052	0.0564	0.0108	1.98999E-07	finn-b-E4_DM2_STRICT	rs11169182	T	C	0.4934	|| id:finn-b-E4_DM2_STRICT	TRUE	reported	igd	27.27160494
234	12	50996381	-0.1344	0.0322	3.01502E-05	finn-b-E4_DM2_STRICT	rs74719891	T	C	0.02966	|| id:finn-b-E4_DM2_STRICT	TRUE	reported	igd	17.42155009
235	12	133088165	0.0561	0.0121	3.37101E-06	finn-b-E4_DM2_STRICT	rs3751297	A	G	0.2891	|| id:finn-b-E4_DM2_STRICT	TRUE	reported	igd	21.49586777
236	12	3594524	-0.0802	0.0179	7.56606E-06	finn-b-E4_DM2_STRICT	rs35507930	A	T	0.1054	|| id:finn-b-E4_DM2_STRICT	TRUE	reported	igd	20.07440467
237	12	4521511	-0.2426	0.0285	1.68694E-17	finn-b-E4_DM2_STRICT	rs78470967	A	T	0.03949	|| id:finn-b-E4_DM2_STRICT	TRUE	reported	igd	72.45892275
238	12	97504719	-0.056	0.0127	1.09099E-05	finn-b-E4_DM2_STRICT	rs7306319	G	A	0.7648	|| id:finn-b-E4_DM2_STRICT	TRUE	reported	igd	19.44323889
239	12	97845419	-0.11	0.0215	3.11401E-07	finn-b-E4_DM2_STRICT	rs113475876	G	A	0.06857	|| id:finn-b-E4_DM2_STRICT	TRUE	reported	igd	26.17631152
240	12	121432117	-0.0785	0.0121	8.3888E-11	finn-b-E4_DM2_STRICT	rs56348580	C	G	0.2829	|| id:finn-b-E4_DM2_STRICT	TRUE	reported	igd	42.08899665
241	12	123913912	0.1605	0.0314	3.27198E-07	finn-b-E4_DM2_STRICT	rs141433349	A	G	0.03137	|| id:finn-b-E4_DM2_STRICT	TRUE	reported	igd	26.12707412
242	12	71550697	-0.0598	0.0109	3.57701E-08	finn-b-E4_DM2_STRICT	rs1798085	C	T	0.5575	|| id:finn-b-E4_DM2_STRICT	TRUE	reported	igd	30.09881323
243	12	27959201	-0.0653	0.0128	3.58501E-07	finn-b-E4_DM2_STRICT	rs10466811	A	G	0.2375	|| id:finn-b-E4_DM2_STRICT	TRUE	reported	igd	26.02593994
244	13	51096095	-0.0557	0.0133	2.82501E-05	finn-b-E4_DM2_STRICT	rs963740	T	A	0.2111	|| id:finn-b-E4_DM2_STRICT	TRUE	reported	igd	17.53909209
245	13	80718654	-0.0769	0.0112	5.55776E-12	finn-b-E4_DM2_STRICT	rs7998259	A	G	0.3896	|| id:finn-b-E4_DM2_STRICT	TRUE	reported	igd	47.14293686
246	13	54655661	-0.2319	0.0568	4.38198E-05	finn-b-E4_DM2_STRICT	rs35036268	G	C	0.009779	|| id:finn-b-E4_DM2_STRICT	TRUE	reported	igd	16.66881881
247	13	37236354	0.0643	0.0119	7.25103E-08	finn-b-E4_DM2_STRICT	rs60472952	T	A	0.2887	|| id:finn-b-E4_DM2_STRICT	TRUE	reported	igd	29.19631382
248	13	108807665	-0.1404	0.0307	4.76705E-06	finn-b-E4_DM2_STRICT	rs9520808	A	C	0.9673	|| id:finn-b-E4_DM2_STRICT	TRUE	reported	igd	20.91498053
249	13	111253617	-0.0697	0.0165	2.39999E-05	finn-b-E4_DM2_STRICT	rs9583513	T	A	0.1221	|| id:finn-b-E4_DM2_STRICT	TRUE	reported	igd	17.84422406
250	13	100718703	0.1177	0.0262	6.78094E-06	finn-b-E4_DM2_STRICT	rs9517966	C	A	0.04634	|| id:finn-b-E4_DM2_STRICT	TRUE	reported	igd	20.18135598
251	13	34801045	0.6051	0.1436	2.50998E-05	finn-b-E4_DM2_STRICT	rs73176438	A	G	0.00151	|| id:finn-b-E4_DM2_STRICT	TRUE	reported	igd	17.75601184
252	13	23813949	0.2457	0.0576	1.99301E-05	finn-b-E4_DM2_STRICT	rs142946996	A	G	0.009289	|| id:finn-b-E4_DM2_STRICT	TRUE	reported	igd	18.19555664
253	14	79703248	0.069	0.0128	7.20493E-08	finn-b-E4_DM2_STRICT	rs12895355	A	C	0.2385	|| id:finn-b-E4_DM2_STRICT	TRUE	reported	igd	29.05883789
254	14	32941522	0.0546	0.0115	0.00000213	finn-b-E4_DM2_STRICT	rs10146289	A	G	0.3425	|| id:finn-b-E4_DM2_STRICT	TRUE	reported	igd	22.54185255
255	14	101154072	-0.0488	0.0109	8.16094E-06	finn-b-E4_DM2_STRICT	rs4905994	C	T	0.5678	|| id:finn-b-E4_DM2_STRICT	TRUE	reported	igd	20.04410403
256	14	24301918	-0.0629	0.0153	3.91003E-05	finn-b-E4_DM2_STRICT	rs4982814	C	T	0.1478	|| id:finn-b-E4_DM2_STRICT	TRUE	reported	igd	16.90123457
257	14	35755737	0.0526	0.0122	1.64699E-05	finn-b-E4_DM2_STRICT	rs4982254	T	C	0.2749	|| id:finn-b-E4_DM2_STRICT	TRUE	reported	igd	18.58882021
258	14	92739145	0.0762	0.0178	1.93299E-05	finn-b-E4_DM2_STRICT	rs4900106	C	T	0.1047	|| id:finn-b-E4_DM2_STRICT	TRUE	reported	igd	18.32609519
259	14	101815371	0.0908	0.0213	1.93999E-05	finn-b-E4_DM2_STRICT	rs117817314	C	T	0.07057	|| id:finn-b-E4_DM2_STRICT	TRUE	reported	igd	18.17240847
260	14	29702590	-0.0529	0.0111	0.000001859	finn-b-E4_DM2_STRICT	rs12887636	G	T	0.398	|| id:finn-b-E4_DM2_STRICT	TRUE	reported	igd	22.71252333
261	15	62291191	-0.1283	0.0229	2.17801E-08	finn-b-E4_DM2_STRICT	rs28553330	C	T	0.06049	|| id:finn-b-E4_DM2_STRICT	TRUE	reported	igd	31.38935184
262	15	77892857	-0.0576	0.011	1.79201E-07	finn-b-E4_DM2_STRICT	rs58102377	G	A	0.4097	|| id:finn-b-E4_DM2_STRICT	TRUE	reported	igd	27.41950413
263	15	91523036	0.0559	0.0124	6.67498E-06	finn-b-E4_DM2_STRICT	rs6496742	T	C	0.2534	|| id:finn-b-E4_DM2_STRICT	TRUE	reported	igd	20.32264568
264	15	93823650	-0.0634	0.0142	8.03508E-06	finn-b-E4_DM2_STRICT	rs8032983	C	G	0.1783	|| id:finn-b-E4_DM2_STRICT	TRUE	reported	igd	19.93433842
265	15	41112425	-0.211	0.0474	8.38205E-06	finn-b-E4_DM2_STRICT	rs191907775	T	C	0.01381	|| id:finn-b-E4_DM2_STRICT	TRUE	reported	igd	19.81564564
266	15	25469752	0.4495	0.0982	4.70901E-06	finn-b-E4_DM2_STRICT	rs140188869	A	G	0.003159	|| id:finn-b-E4_DM2_STRICT	TRUE	reported	igd	20.95252737
267	15	76448796	0.0577	0.0131	9.78408E-06	finn-b-E4_DM2_STRICT	rs2935971	A	G	0.2219	|| id:finn-b-E4_DM2_STRICT	TRUE	reported	igd	19.40032632
268	15	40640632	-0.0536	0.011	1.15601E-06	finn-b-E4_DM2_STRICT	rs4605132	C	T	0.5149	|| id:finn-b-E4_DM2_STRICT	TRUE	reported	igd	23.74347107
269	15	61124235	0.0549	0.0118	3.37901E-06	finn-b-E4_DM2_STRICT	rs341392	A	C	0.6979	|| id:finn-b-E4_DM2_STRICT	TRUE	reported	igd	21.64615053
270	16	77261943	0.1307	0.0232	1.837E-08	finn-b-E4_DM2_STRICT	rs12449219	G	C	0.05833	|| id:finn-b-E4_DM2_STRICT	TRUE	reported	igd	31.73768207
271	16	53818167	0.1185	0.011	3.53021E-27	finn-b-E4_DM2_STRICT	rs9933509	C	T	0.4126	|| id:finn-b-E4_DM2_STRICT	TRUE	reported	igd	116.0516529
272	16	86118845	0.0724	0.0157	0.000004032	finn-b-E4_DM2_STRICT	rs56253896	A	G	0.1388	|| id:finn-b-E4_DM2_STRICT	TRUE	reported	igd	21.26560915
273	16	86131731	-0.0463	0.0111	3.02901E-05	finn-b-E4_DM2_STRICT	rs6540269	C	T	0.6077	|| id:finn-b-E4_DM2_STRICT	TRUE	reported	igd	17.39866894
274	16	88525486	0.0835	0.0173	1.42899E-06	finn-b-E4_DM2_STRICT	rs55767424	A	G	0.1156	|| id:finn-b-E4_DM2_STRICT	TRUE	reported	igd	23.29596712
275	16	1246270	0.0462	0.0109	2.07702E-05	finn-b-E4_DM2_STRICT	rs1005454	G	A	0.4951	|| id:finn-b-E4_DM2_STRICT	TRUE	reported	igd	17.96515445
276	16	75213917	-0.1369	0.0327	2.92503E-05	finn-b-E4_DM2_STRICT	rs144247165	A	G	0.02994	|| id:finn-b-E4_DM2_STRICT	TRUE	reported	igd	17.52715353
277	16	75236763	-0.1646	0.0194	2.10378E-17	finn-b-E4_DM2_STRICT	rs55993634	G	C	0.08714	|| id:finn-b-E4_DM2_STRICT	TRUE	reported	igd	71.98735253
278	17	47090785	-0.0527	0.0119	9.35707E-06	finn-b-E4_DM2_STRICT	rs11079849	T	C	0.299	|| id:finn-b-E4_DM2_STRICT	TRUE	reported	igd	19.6122449
279	17	63009717	-0.1566	0.0359	1.25101E-05	finn-b-E4_DM2_STRICT	rs41300620	C	T	0.02542	|| id:finn-b-E4_DM2_STRICT	TRUE	reported	igd	19.02806465
280	17	36047417	0.0729	0.0134	5.22396E-08	finn-b-E4_DM2_STRICT	rs3110641	G	A	0.7866	|| id:finn-b-E4_DM2_STRICT	TRUE	reported	igd	29.59684785
281	17	19208340	0.1401	0.0335	2.83798E-05	finn-b-E4_DM2_STRICT	rs62065871	T	C	0.02725	|| id:finn-b-E4_DM2_STRICT	TRUE	reported	igd	17.48987302
282	17	76496183	0.0694	0.0171	4.86497E-05	finn-b-E4_DM2_STRICT	rs2028733	A	G	0.1145	|| id:finn-b-E4_DM2_STRICT	TRUE	reported	igd	16.47125611
283	17	58037374	0.0631	0.0141	7.46707E-06	finn-b-E4_DM2_STRICT	rs12600680	C	T	0.179	|| id:finn-b-E4_DM2_STRICT	TRUE	reported	igd	20.02721191
284	17	4014384	0.0644	0.0117	4.18996E-08	finn-b-E4_DM2_STRICT	rs7224685	T	G	0.3016	|| id:finn-b-E4_DM2_STRICT	TRUE	reported	igd	30.29702681
285	17	7447064	0.054	0.0124	1.42801E-05	finn-b-E4_DM2_STRICT	rs9900162	G	A	0.2531	|| id:finn-b-E4_DM2_STRICT	TRUE	reported	igd	18.96462019
286	17	9788769	0.055	0.0116	1.95398E-06	finn-b-E4_DM2_STRICT	rs8077150	A	G	0.3223	|| id:finn-b-E4_DM2_STRICT	TRUE	reported	igd	22.48067776
287	17	36103565	-0.0665	0.0113	4.31102E-09	finn-b-E4_DM2_STRICT	rs11263763	A	G	0.646	|| id:finn-b-E4_DM2_STRICT	TRUE	reported	igd	34.63270421
288	18	21103909	-0.0541	0.0109	6.528E-07	finn-b-E4_DM2_STRICT	rs891387	C	T	0.5127	|| id:finn-b-E4_DM2_STRICT	TRUE	reported	igd	24.63437421
289	18	39923815	0.0516	0.0115	7.01795E-06	finn-b-E4_DM2_STRICT	rs428198	G	A	0.3334	|| id:finn-b-E4_DM2_STRICT	TRUE	reported	igd	20.13277883
290	18	68111101	0.1036	0.0253	4.29596E-05	finn-b-E4_DM2_STRICT	rs72952745	A	C	0.049	|| id:finn-b-E4_DM2_STRICT	TRUE	reported	igd	16.76789202
291	18	46119000	0.1819	0.0371	9.31408E-07	finn-b-E4_DM2_STRICT	rs118115683	C	T	0.02215	|| id:finn-b-E4_DM2_STRICT	TRUE	reported	igd	24.03906539
292	18	4889696	0.1155	0.0266	1.45999E-05	finn-b-E4_DM2_STRICT	rs72862560	A	C	0.04344	|| id:finn-b-E4_DM2_STRICT	TRUE	reported	igd	18.85387812
293	18	337406	-0.1129	0.0262	1.69001E-05	finn-b-E4_DM2_STRICT	rs117516062	A	T	0.04714	|| id:finn-b-E4_DM2_STRICT	TRUE	reported	igd	18.56886254
294	18	57826570	0.077	0.014	3.913E-08	finn-b-E4_DM2_STRICT	rs12967878	C	T	0.181	|| id:finn-b-E4_DM2_STRICT	TRUE	reported	igd	30.25
295	18	63348775	0.0741	0.0161	4.42099E-06	finn-b-E4_DM2_STRICT	rs35673518	A	G	0.1286	|| id:finn-b-E4_DM2_STRICT	TRUE	reported	igd	21.18286332
296	18	4766803	-0.177	0.0357	7.25103E-07	finn-b-E4_DM2_STRICT	rs182494976	A	T	0.02569	|| id:finn-b-E4_DM2_STRICT	TRUE	reported	igd	24.58159734
297	19	18824870	-0.0565	0.0108	1.78801E-07	finn-b-E4_DM2_STRICT	rs9304955	G	A	0.5284	|| id:finn-b-E4_DM2_STRICT	TRUE	reported	igd	27.36839849
298	19	21199155	0.2851	0.0688	3.41201E-05	finn-b-E4_DM2_STRICT	rs142104258	G	A	0.006613	|| id:finn-b-E4_DM2_STRICT	TRUE	reported	igd	17.17186866
299	19	45411941	-0.0813	0.0142	1.09401E-08	finn-b-E4_DM2_STRICT	rs429358	C	T	0.1827	|| id:finn-b-E4_DM2_STRICT	TRUE	reported	igd	32.77965681
300	19	7105841	-0.1556	0.0359	0.00001438	finn-b-E4_DM2_STRICT	rs34488330	C	A	0.02392	|| id:finn-b-E4_DM2_STRICT	TRUE	reported	igd	18.78582568
301	19	7161706	-0.1202	0.0252	0.00000178	finn-b-E4_DM2_STRICT	rs16990079	G	A	0.04976	|| id:finn-b-E4_DM2_STRICT	TRUE	reported	igd	22.75138574
302	19	1178121	0.146	0.0359	4.86004E-05	finn-b-E4_DM2_STRICT	rs117434403	A	G	0.02341	|| id:finn-b-E4_DM2_STRICT	TRUE	reported	igd	16.53928818
303	19	40948848	0.2313	0.0481	1.50401E-06	finn-b-E4_DM2_STRICT	rs74595028	C	T	0.0128	|| id:finn-b-E4_DM2_STRICT	TRUE	reported	igd	23.12390161
304	19	46157860	-0.0611	0.0109	1.90599E-08	finn-b-E4_DM2_STRICT	rs7507893	A	G	0.4965	|| id:finn-b-E4_DM2_STRICT	TRUE	reported	igd	31.42168168
305	19	7976529	-0.0675	0.0116	5.75003E-09	finn-b-E4_DM2_STRICT	rs2303700	C	T	0.6741	|| id:finn-b-E4_DM2_STRICT	TRUE	reported	igd	33.86035969
306	19	19393714	0.0975	0.0149	5.73456E-11	finn-b-E4_DM2_STRICT	rs8100204	A	G	0.1597	|| id:finn-b-E4_DM2_STRICT	TRUE	reported	igd	42.81901716
307	19	56509991	-0.2892	0.0687	2.53198E-05	finn-b-E4_DM2_STRICT	rs117108287	T	C	0.006246	|| id:finn-b-E4_DM2_STRICT	TRUE	reported	igd	17.72079098
308	20	57155439	-0.1414	0.0258	4.40798E-08	finn-b-E4_DM2_STRICT	rs117657619	A	G	0.0476	|| id:finn-b-E4_DM2_STRICT	TRUE	reported	igd	30.03719728
309	20	523267	0.0821	0.0187	1.16501E-05	finn-b-E4_DM2_STRICT	rs8122783	A	G	0.09143	|| id:finn-b-E4_DM2_STRICT	TRUE	reported	igd	19.27538677
310	20	43024814	-0.2083	0.0438	1.95299E-06	finn-b-E4_DM2_STRICT	rs149524258	A	G	0.01621	|| id:finn-b-E4_DM2_STRICT	TRUE	reported	igd	22.61675632
311	20	42946966	0.0768	0.0131	4.35101E-09	finn-b-E4_DM2_STRICT	rs6017317	G	T	0.2185	|| id:finn-b-E4_DM2_STRICT	TRUE	reported	igd	34.37002506
312	20	4108058	-0.0528	0.0125	2.26298E-05	finn-b-E4_DM2_STRICT	rs1741330	C	T	0.7455	|| id:finn-b-E4_DM2_STRICT	TRUE	reported	igd	17.842176
313	20	57607363	-0.2295	0.0253	1.17004E-19	finn-b-E4_DM2_STRICT	rs45551238	T	C	0.05006	|| id:finn-b-E4_DM2_STRICT	TRUE	reported	igd	82.2856942
314	20	32819871	-0.0615	0.0116	1.053E-07	finn-b-E4_DM2_STRICT	rs6142129	G	A	0.3299	|| id:finn-b-E4_DM2_STRICT	TRUE	reported	igd	28.10827883
315	20	42011344	0.0885	0.0191	3.65999E-06	finn-b-E4_DM2_STRICT	rs56063675	T	C	0.08884	|| id:finn-b-E4_DM2_STRICT	TRUE	reported	igd	21.46939503
316	20	43050676	0.1758	0.0269	6.69268E-11	finn-b-E4_DM2_STRICT	rs77735929	A	T	0.04278	|| id:finn-b-E4_DM2_STRICT	TRUE	reported	igd	42.71035503
317	21	29485557	0.0589	0.0145	4.68101E-05	finn-b-E4_DM2_STRICT	rs2831514	T	C	0.1705	|| id:finn-b-E4_DM2_STRICT	TRUE	reported	igd	16.50040428
318	21	16850560	0.3269	0.0801	4.51502E-05	finn-b-E4_DM2_STRICT	rs78466584	A	T	0.00484	|| id:finn-b-E4_DM2_STRICT	TRUE	reported	igd	16.6557736
319	22	46576036	0.1204	0.0295	4.49004E-05	finn-b-E4_DM2_STRICT	rs77561714	C	T	0.03583	|| id:finn-b-E4_DM2_STRICT	TRUE	reported	igd	16.65746625
320	22	20796117	-0.0744	0.0118	2.939E-10	finn-b-E4_DM2_STRICT	rs8353	T	G	0.3009	|| id:finn-b-E4_DM2_STRICT	TRUE	reported	igd	39.75409365
321	22	18583014	-0.0513	0.0124	0.00003539	finn-b-E4_DM2_STRICT	rs361569	C	T	0.2564	|| id:finn-b-E4_DM2_STRICT	TRUE	reported	igd	17.11556972
322	22	30045421	-0.1386	0.0294	0.0000025	finn-b-E4_DM2_STRICT	rs145858636	C	G	0.03514	|| id:finn-b-E4_DM2_STRICT	TRUE	reported	igd	22.2244898
