# Supplementary material for: Association between obstructive sleep apnea and chronic kidney disease: A cross-sectional and Mendelian randomization study
Source: Medicine (Baltimore). 2025 Feb 7;104(6):e41437. doi: 10.1097/MD.0000000000041437 (PMC11812998; doi:10.1097/MD.0000000000041437)

Supplementary document 2

The result of the leave-one-out method in OSA to eGFR (cystatin c)

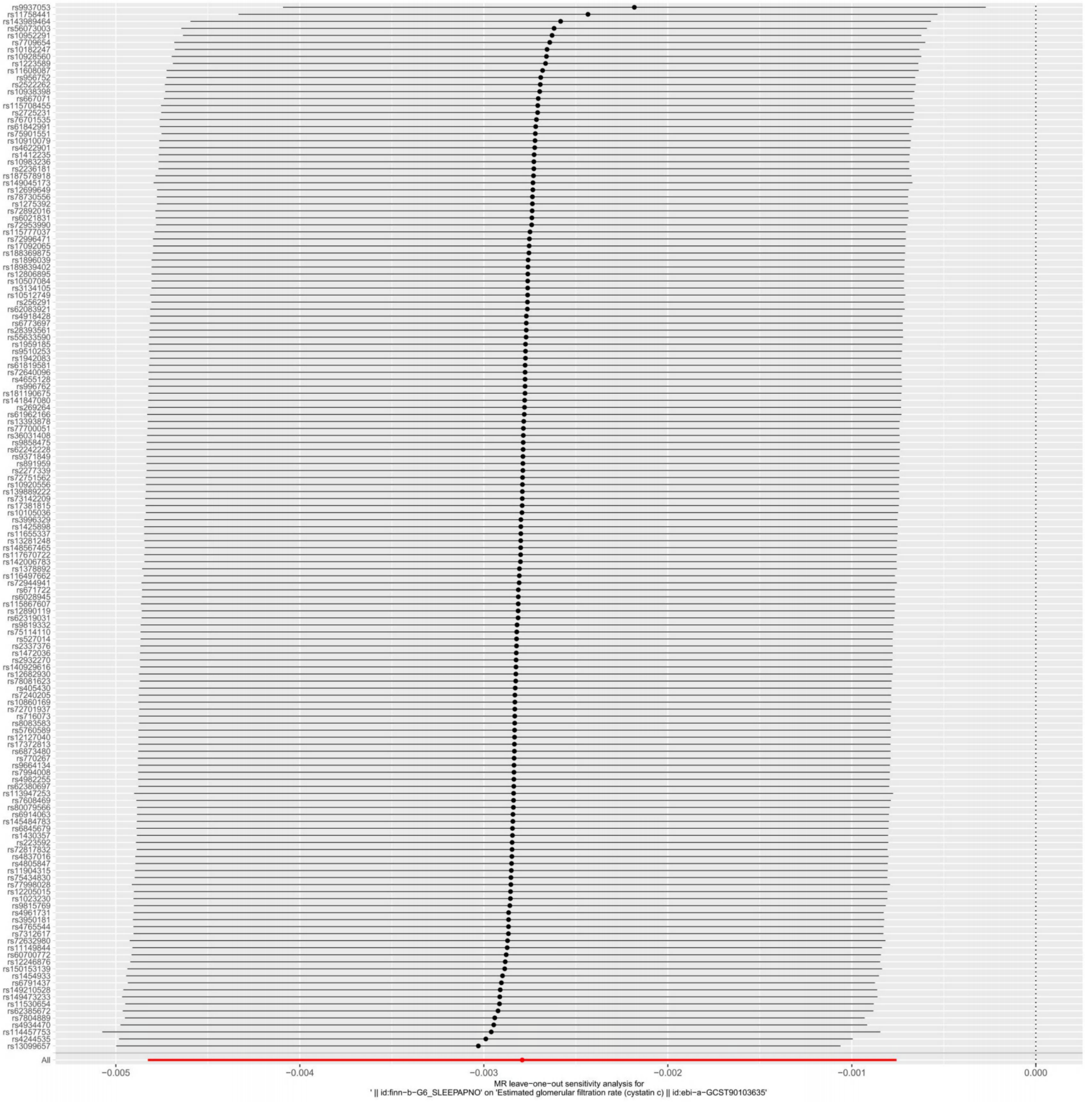

The result of the leave-one-out method in OSA to BUN

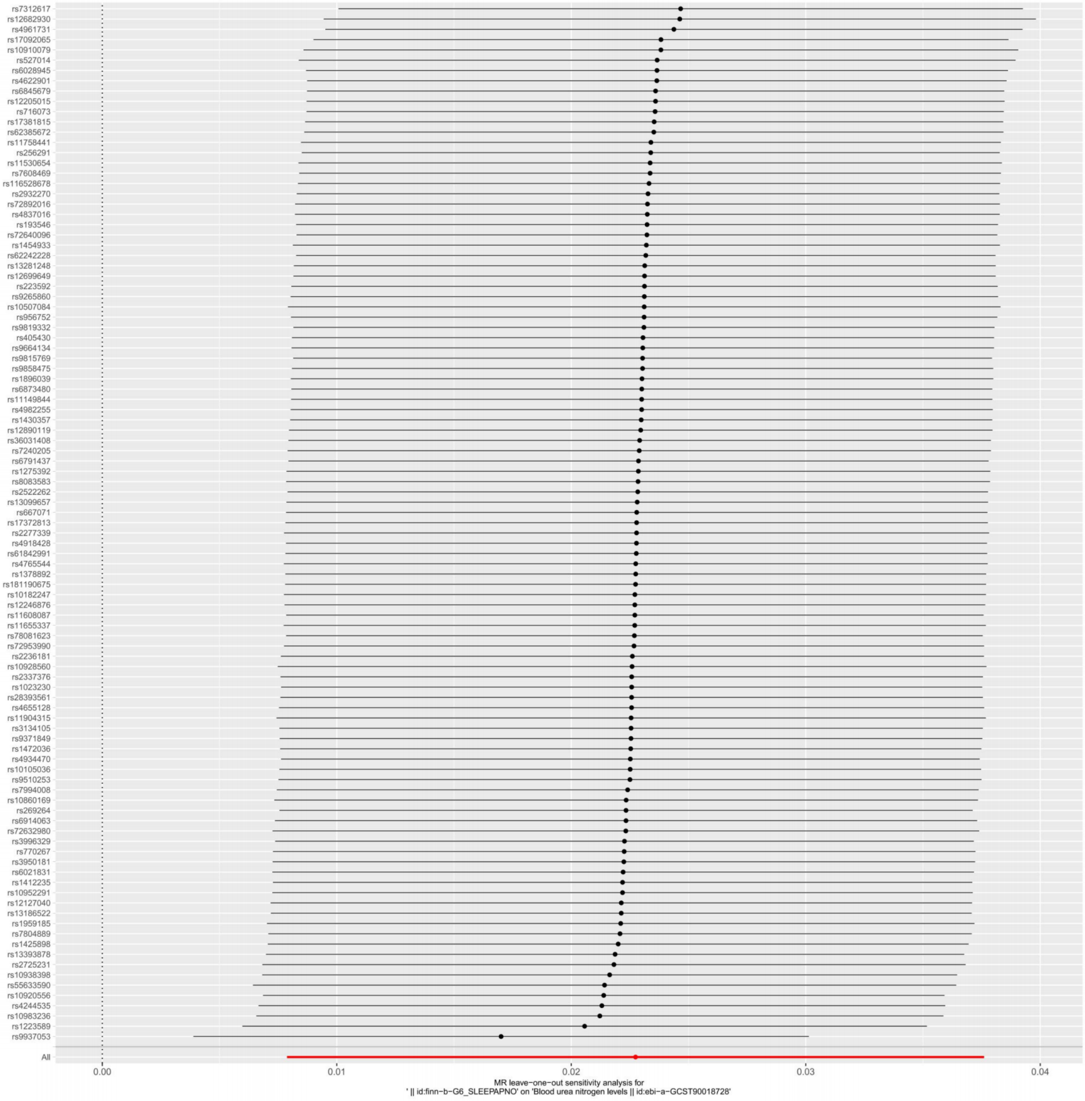

The result of the leave-one-out method in OSA to creatinine levels

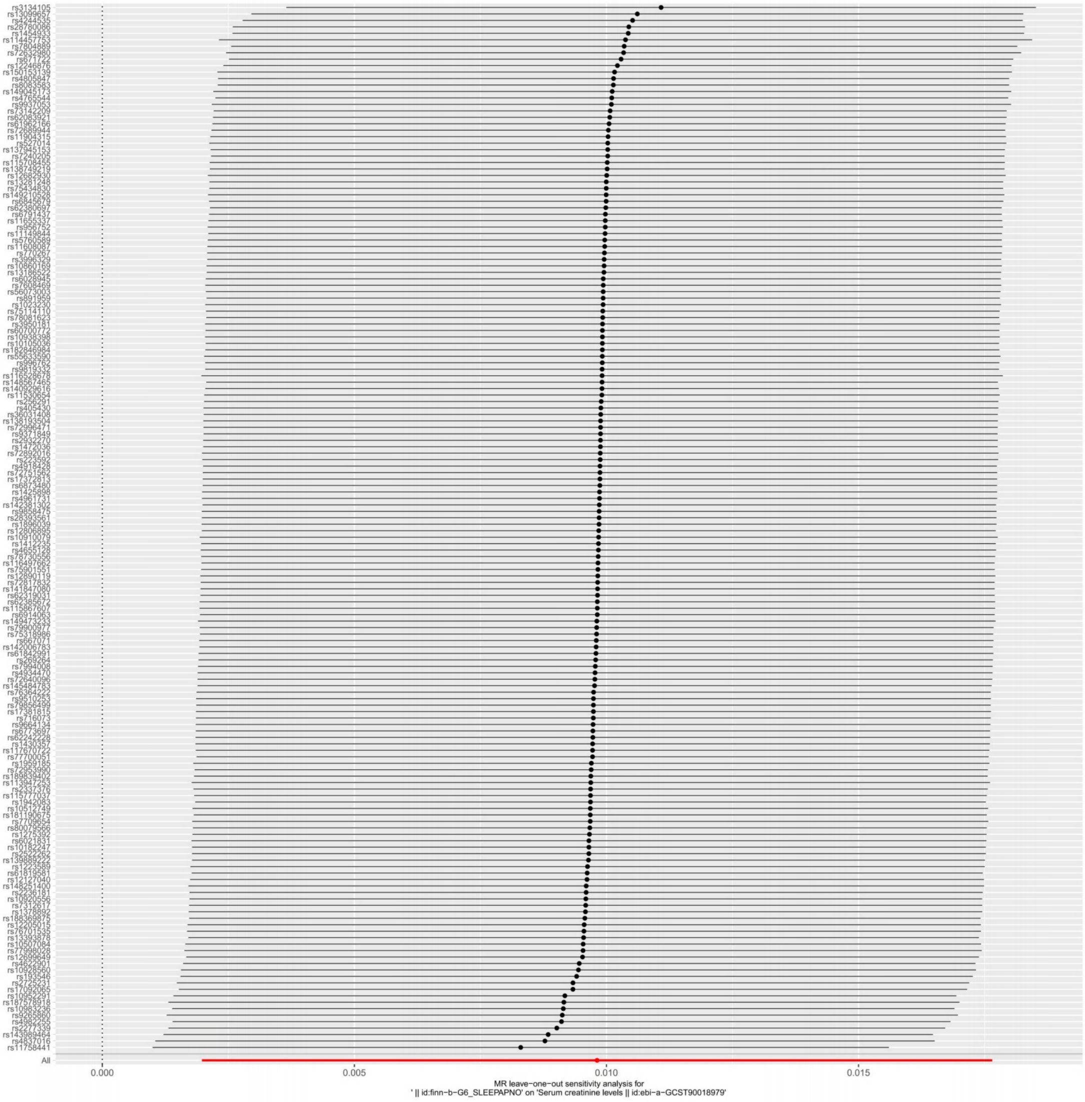

The result of the leave-one-out method in OSA to cystatin C levels

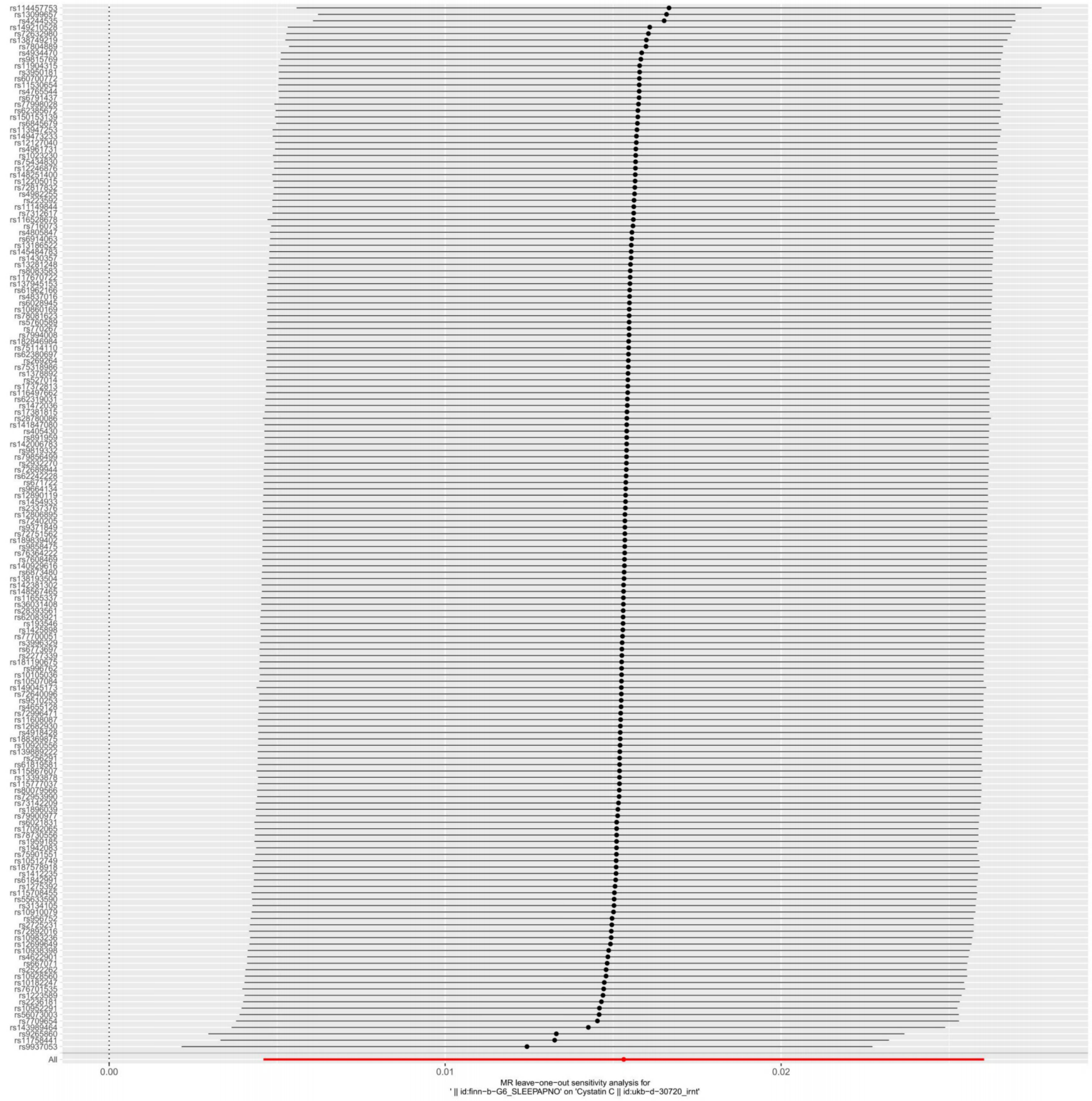

The result of the leave-one-out method in OSA to obesity

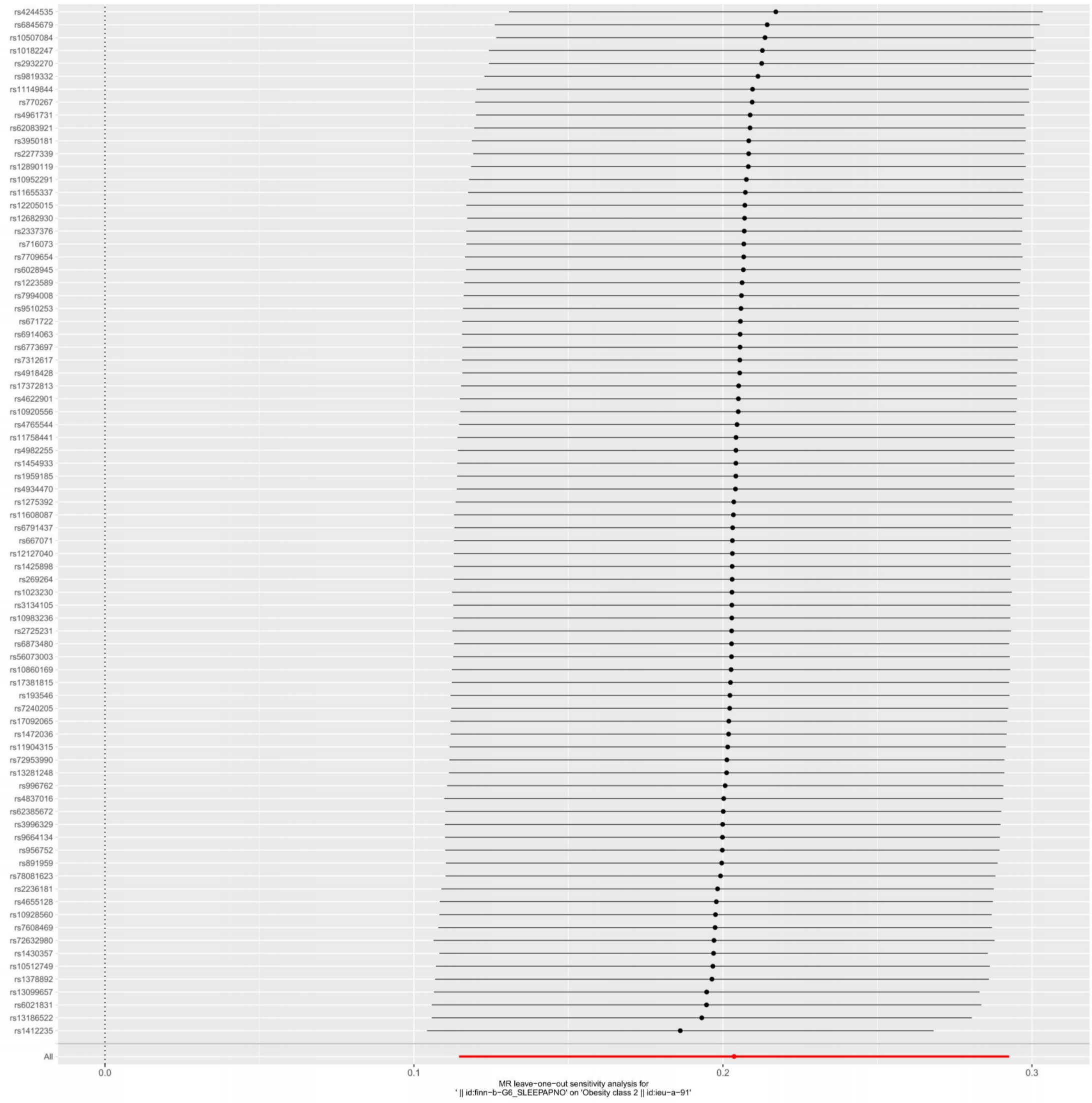

The result of the leave-one-out method in OSA to hypertension

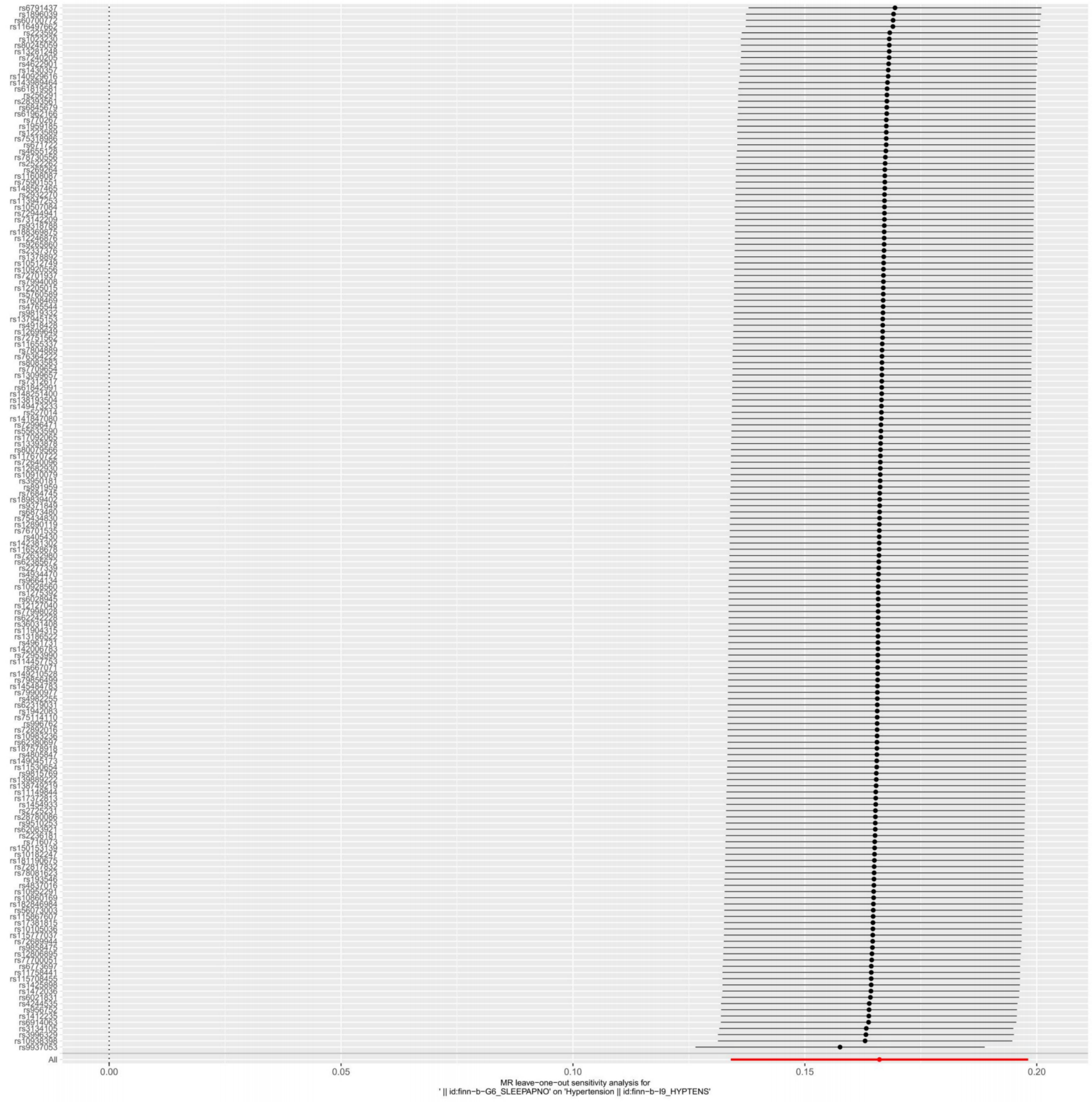

The result of the leave-one-out method in OSA to T2DM

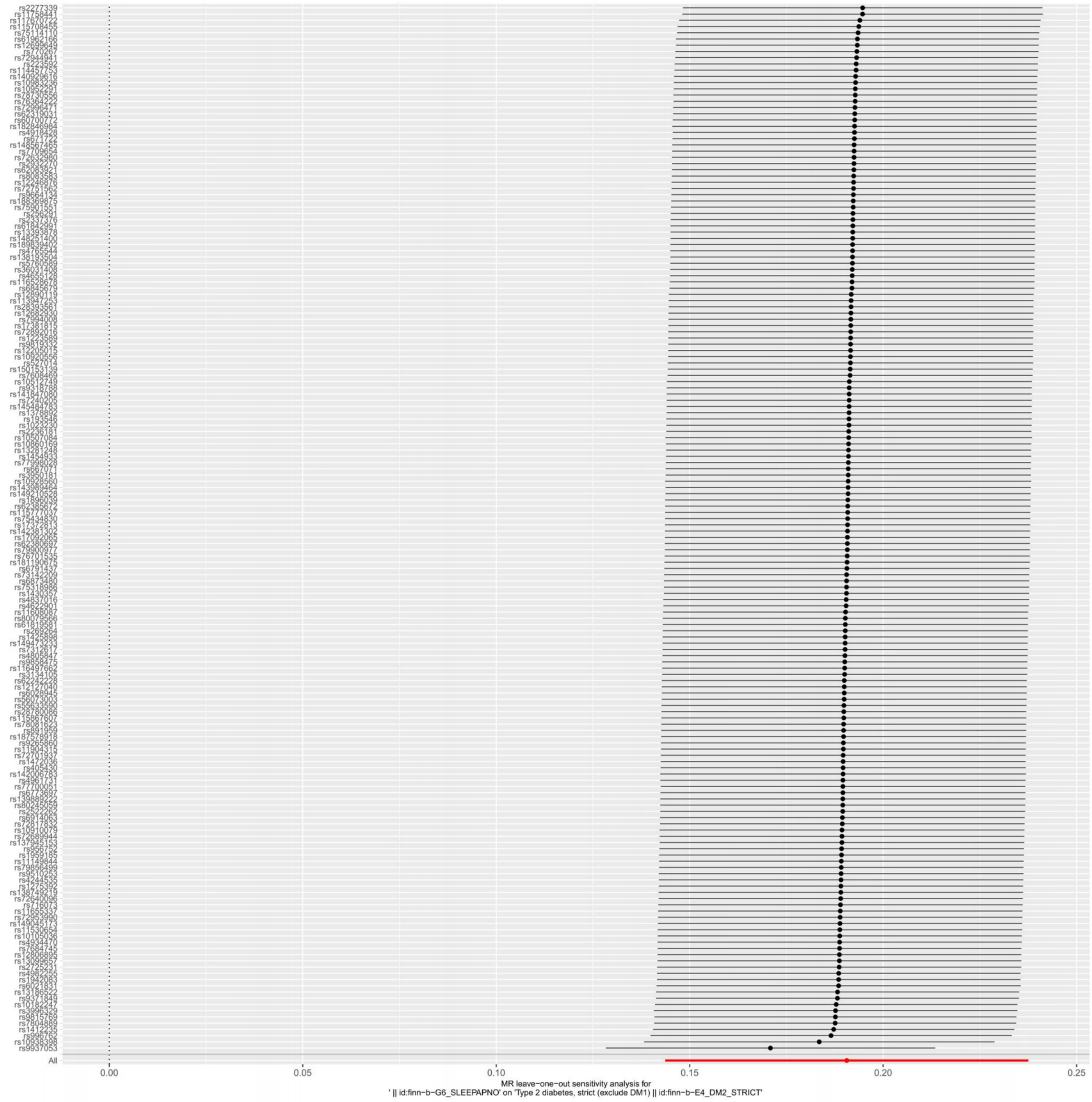

The result of the leave-one-out method in obesity to eGFR(cystatin c)

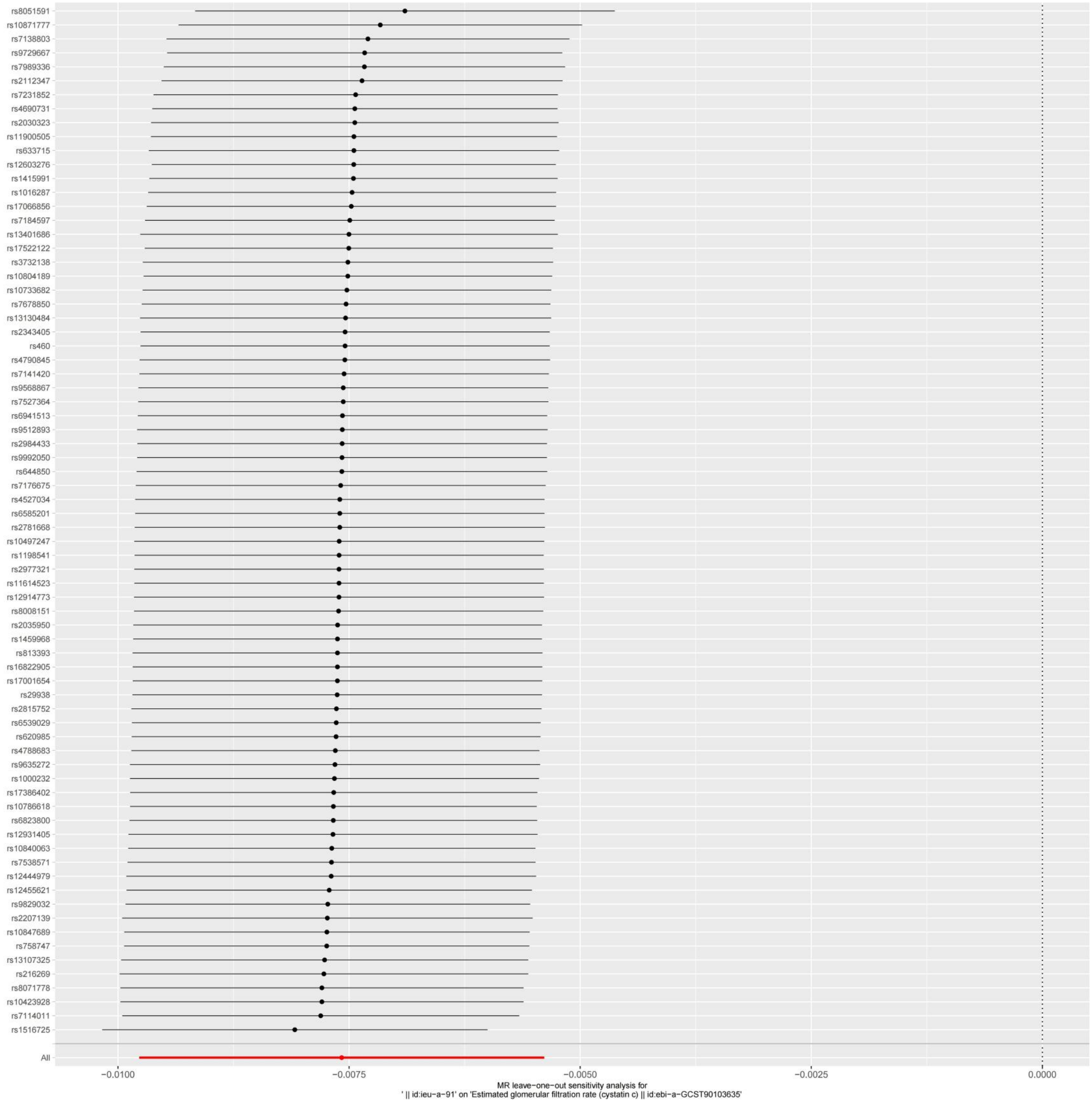

The result of the leave-one-out method in obesity to BUN

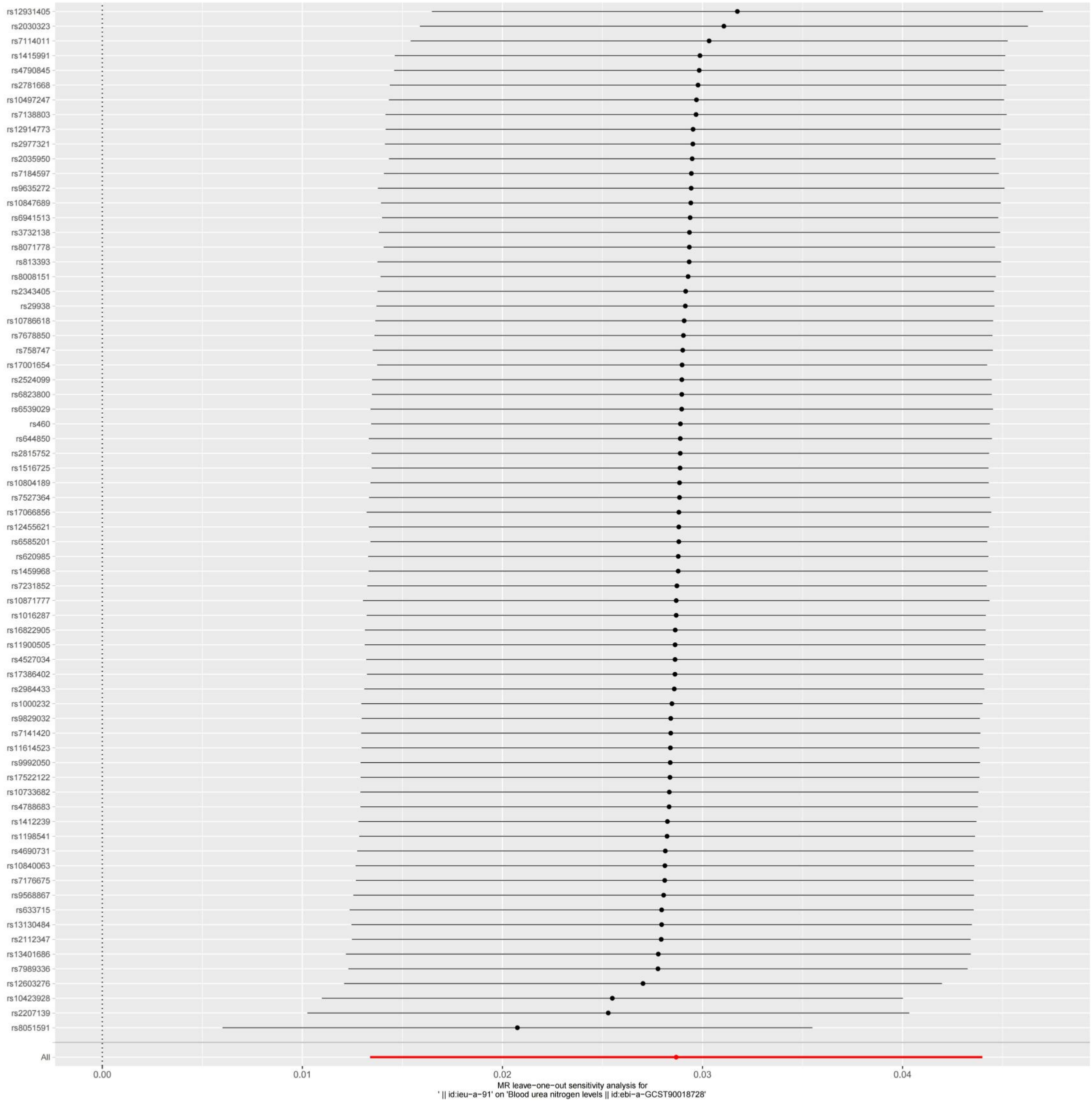

The result of the leave-one-out method in obesity to cystatin C levels

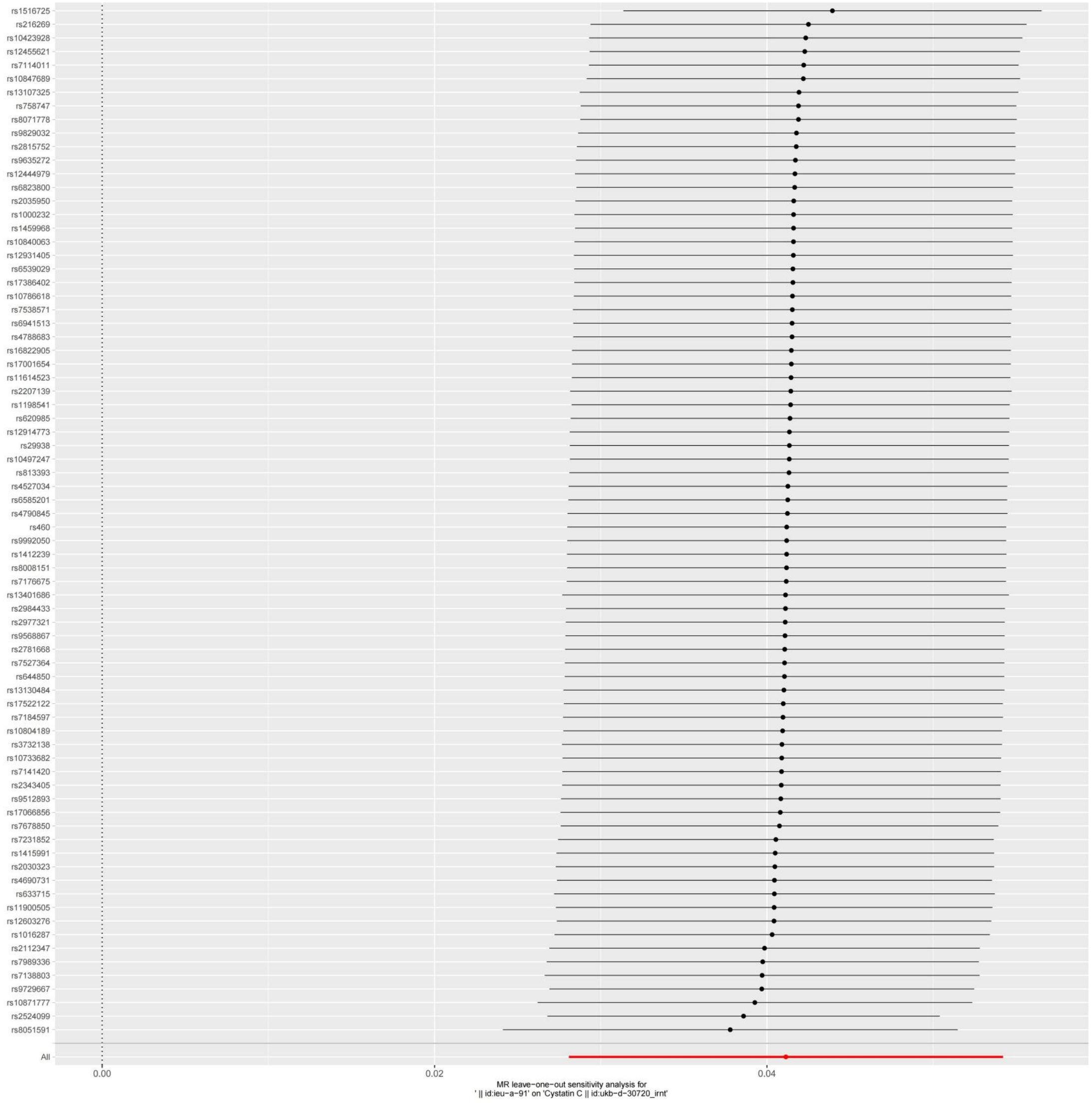

The result of the leave-one-out method in hypertension to eGFR(cystatin c)

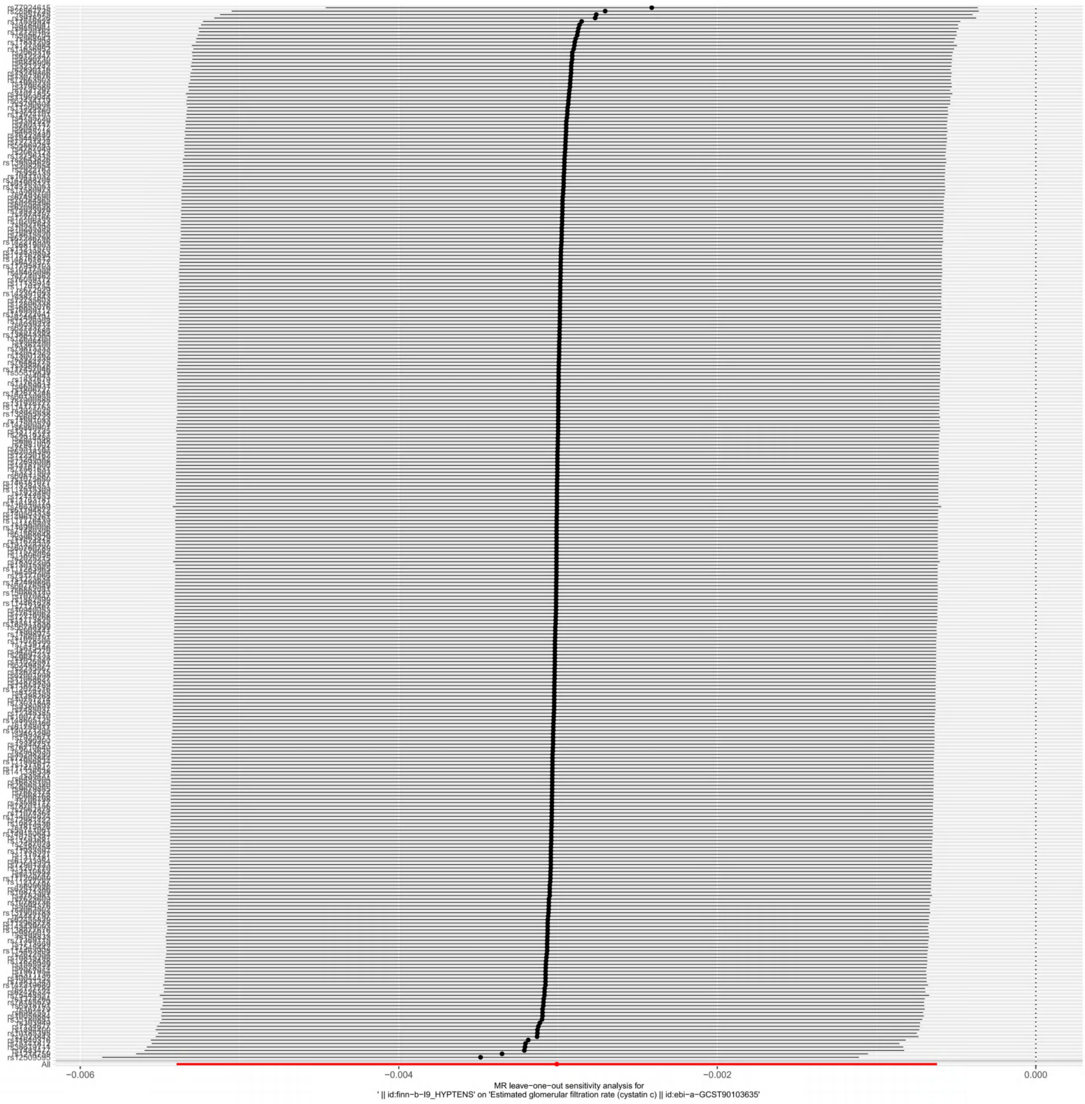

The result of the leave-one-out method in hypertension to cystatin C levels

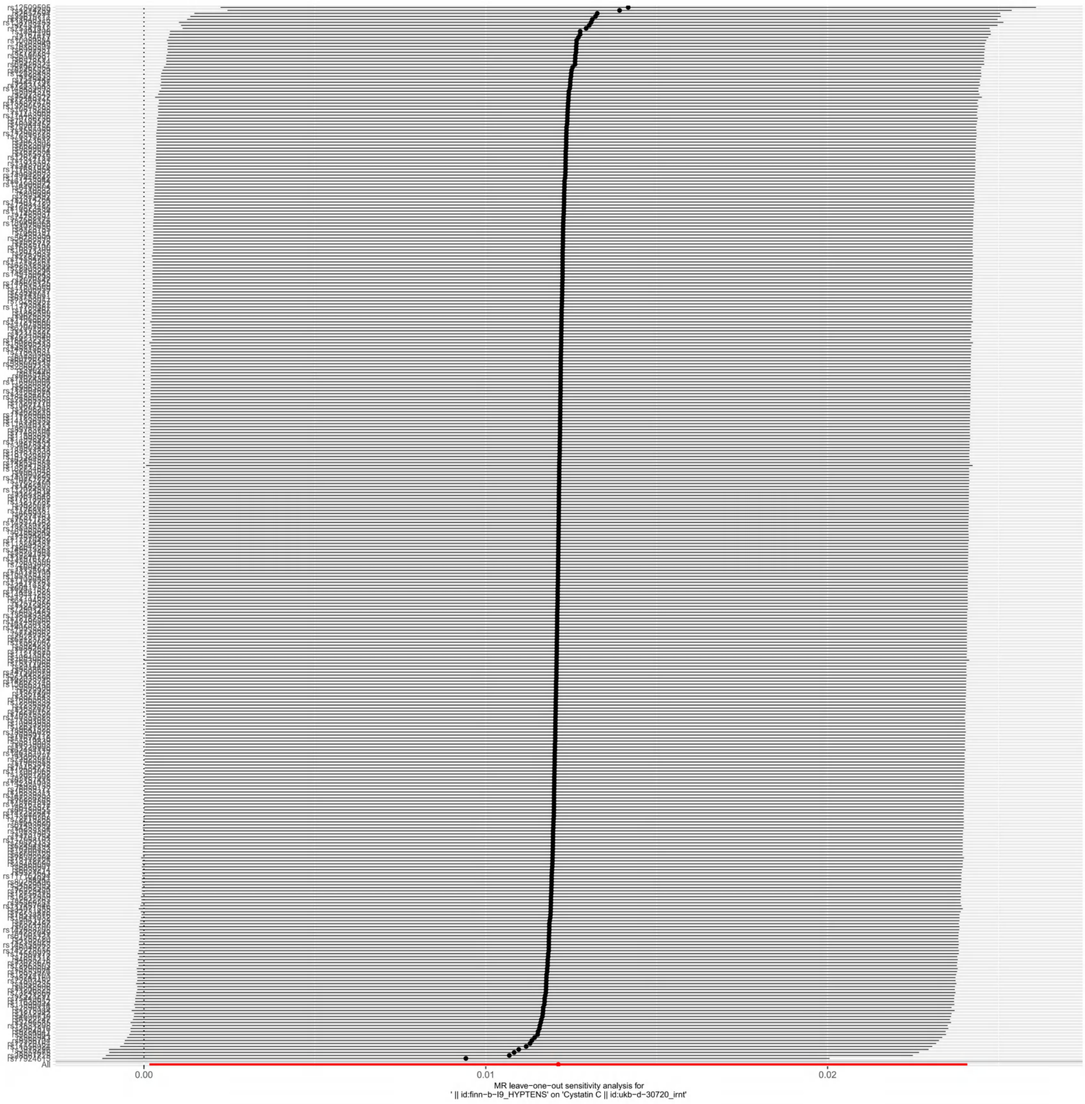

The result of the leave-one-out method in T2DM to eGFR(cystatin c)

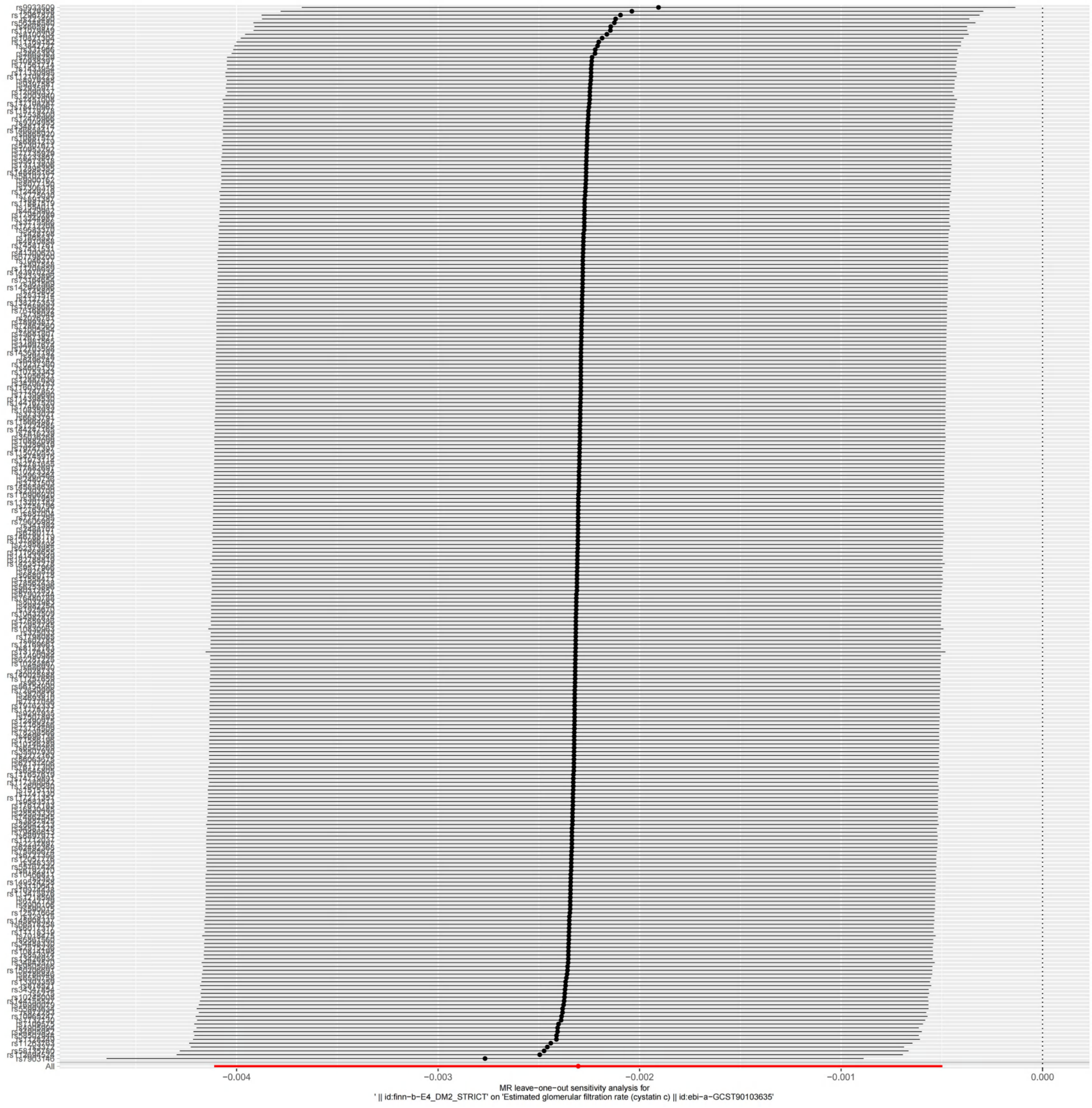

The result of the leave-one-out method in T2DM to BUN

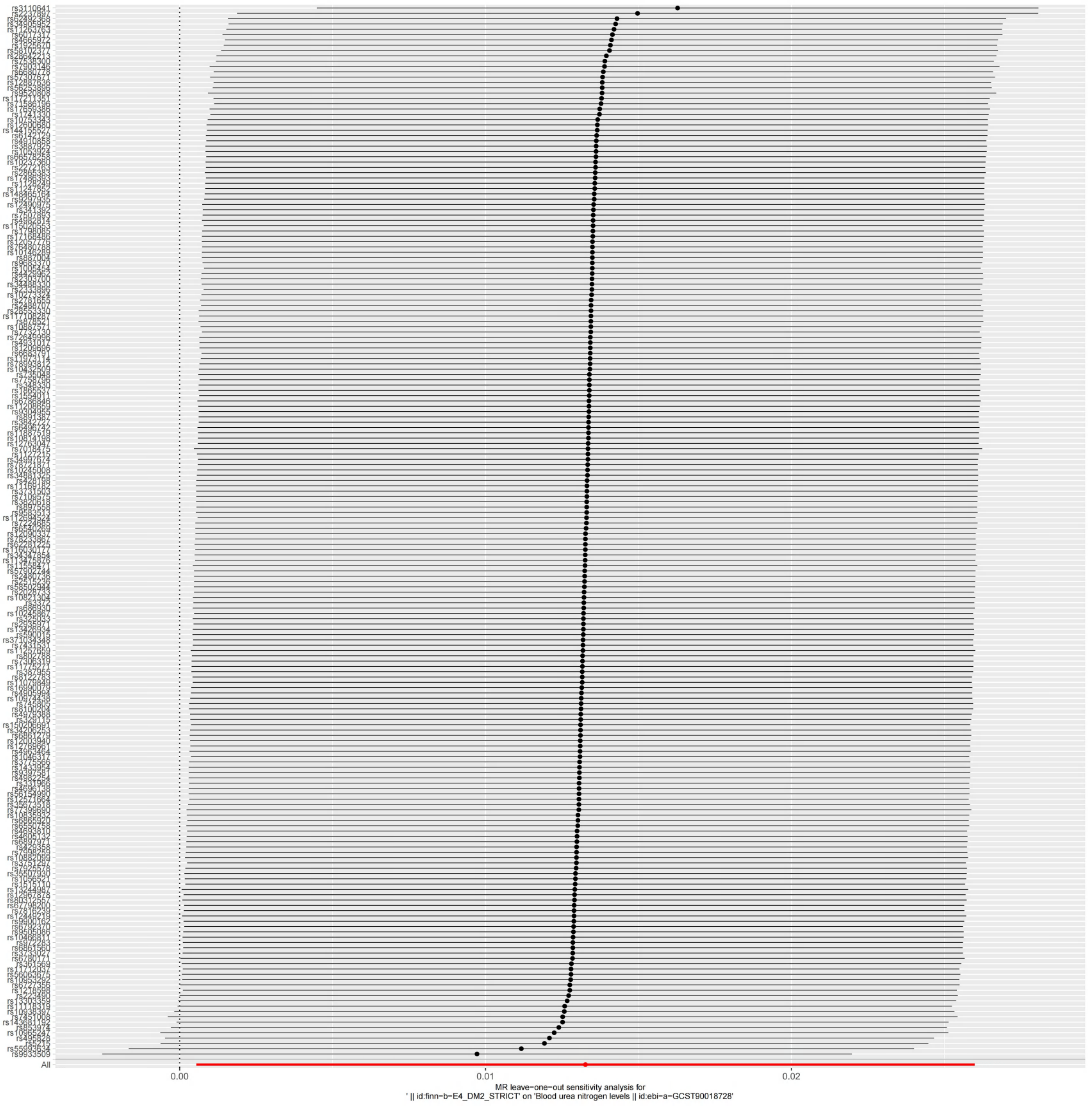

Supplement: Supplementary file 2 [file medi-104-e41437-s002.pdf]
