# Supplementary material for: Association between obstructive sleep apnea and chronic kidney disease: A cross-sectional and Mendelian randomization study
Source: Medicine (Baltimore). 2025 Feb 7;104(6):e41437. doi: 10.1097/MD.0000000000041437 (PMC11812998; doi:10.1097/MD.0000000000041437)
Supplement: Supplementary file 3 [file medi-104-e41437-s003.docx]

**Supplementary document 3**

**Results of MRPRESSO by R software**

**Sleep apnoea to Estimated glomerular filtration rate (cystatin c)**

$`Main MR results`

Exposure MR Analysis Causal Estimate Sd T-stat

1 beta.exposure Raw -0.0009567323 0.0007334620 -1.3044062

2 beta.exposure Outlier-corrected -0.0004675886 0.0005237161 -0.8928284

P-value

1 0.1940668

2 0.3734387

$`MR-PRESSO results`

$`MR-PRESSO results`$`Global Test`

$`MR-PRESSO results`$`Global Test`$RSSobs

[1] 430.4317

$`MR-PRESSO results`$`Global Test`$Pvalue

1. "<0.001"

$`MR-PRESSO results`$`Distortion Test`

$`MR-PRESSO results`$`Distortion Test`$`Outliers Indices`

[1] 2 12 24 36 47 81 136 152

$`MR-PRESSO results`$`Distortion Test`$`Distortion Coefficient`

beta.exposure

-104.6099

$`MR-PRESSO results`$`Distortion Test`$Pvalue

[1] 0.192

**Sleep apnoea to Blood urea nitrogen levels**

$`Main MR results`

Exposure MR Analysis Causal Estimate Sd T-stat

1 beta.exposure Raw 0.007779982 0.004253747 1.828971

2 beta.exposure Outlier-corrected 0.017434021 0.005523811 3.156158

P-value

1 0.070242106

2 0.002096671

$`MR-PRESSO results`

$`MR-PRESSO results`$`Global Test`

$`MR-PRESSO results`$`Global Test`$RSSobs

[1] 212.0401

$`MR-PRESSO results`$`Global Test`$Pvalue

[1] "<0.001"

$`MR-PRESSO results`$`Distortion Test`

$`MR-PRESSO results`$`Distortion Test`$`Outliers Indices`

[1] 98 106

$`MR-PRESSO results`$`Distortion Test`$`Distortion Coefficient`

beta.exposure

-55.37471

$`MR-PRESSO results`$`Distortion Test`$Pvalue

1. 0.107

**Sleep apnoea to Serum creatinine levels**

$`Main MR results`

Exposure MR Analysis Causal Estimate Sd T-stat

1 beta.exposure Raw 0.003467351 0.002720015 1.274754

2 beta.exposure Outlier-corrected 0.004445012 0.002015674 2.205224

P-value

1 0.20418836

2 0.02895032

$`MR-PRESSO results`

$`MR-PRESSO results`$`Global Test`

$`MR-PRESSO results`$`Global Test`$RSSobs

[1] 551.8573

$`MR-PRESSO results`$`Global Test`$Pvalue

[1] "<0.001"

$`MR-PRESSO results`$`Distortion Test`

$`MR-PRESSO results`$`Distortion Test`$`Outliers Indices`

[1] 12 13 25 37 53 77 86 91 96 100 117 147 157 158

$`MR-PRESSO results`$`Distortion Test`$`Distortion Coefficient`

beta.exposure

-21.99457

$`MR-PRESSO results`$`Distortion Test`$Pvalue

[1] 0.512

**Sleep apnoea to Cystatin C**

$`Main MR results`

Exposure MR Analysis Causal Estimate Sd T-stat P-value

1 beta.exposure Raw 0.005458550 0.004008119 1.361873 0.1750832

2 beta.exposure Outlier-corrected 0.002961103 0.002866824 1.032886 0.3032870

$`MR-PRESSO results`

$`MR-PRESSO results`$`Global Test`

$`MR-PRESSO results`$`Global Test`$RSSobs

[1] 670.9733

$`MR-PRESSO results`$`Global Test`$Pvalue

1. "<0.001"

$`MR-PRESSO results`$`Distortion Test`

$`MR-PRESSO results`$`Distortion Test`$`Outliers Indices`

[1] 12 25 30 37 53 76 91 103 147 157 158 166

$`MR-PRESSO results`$`Distortion Test`$`Distortion Coefficient`

beta.exposure

[1] 84.34179

$`MR-PRESSO results`$`Distortion Test`$Pvalue

[1] 0.109

**Sleep apnoea to Hypertension**

$`Main MR results`

Exposure MR Analysis Causal Estimate Sd T-stat

1 beta.exposure Raw 0.08525414 0.01323077 6.443629

2 beta.exposure Outlier-corrected 0.10039058 0.01323229 7.586789

P-value

1 1.104497e-09

2 2.095405e-12

$`MR-PRESSO results`

$`MR-PRESSO results`$`Global Test`

$`MR-PRESSO results`$`Global Test`$RSSobs

[1] 385.8865

$`MR-PRESSO results`$`Global Test`$Pvalue

[1] "<0.001"

$`MR-PRESSO results`$`Distortion Test`

$`MR-PRESSO results`$`Distortion Test`$`Outliers Indices`

[1] 11 88 124 166 174

$`MR-PRESSO results`$`Distortion Test`$`Distortion Coefficient`

beta.exposure

-15.07755

$`MR-PRESSO results`$`Distortion Test`$Pvalue

[1] 0.486

**Sleep apnoea to obesity**

$`Main MR results`

Exposure MR Analysis Causal Estimate Sd T-stat

1 beta.exposure Raw 0.11441981 0.04414747 2.591764

2 beta.exposure Outlier-corrected 0.07027923 0.02579333 2.724706

P-value

1 0.011258397

2 0.007864561

$`MR-PRESSO results`

$`MR-PRESSO results`$`Global Test`

$`MR-PRESSO results`$`Global Test`$RSSobs

[1] 316.573

$`MR-PRESSO results`$`Global Test`$Pvalue

1. "<0.001"

$`MR-PRESSO results`$`Distortion Test`

$`MR-PRESSO results`$`Distortion Test`$`Outliers Indices`

[1] 26 84

$`MR-PRESSO results`$`Distortion Test`$`Distortion Coefficient`

beta.exposure

[1] 62.80742

$`MR-PRESSO results`$`Distortion Test`$Pvalue

[1] 0.103

**Sleep apnoea to T2DM**

$`Main MR results`

Exposure MR Analysis Causal Estimate Sd T-stat

1 beta.exposure Raw 0.1145899 0.01798822 6.370272

2 beta.exposure Outlier-corrected 0.1215436 0.01677688 7.244707

P-value

1 1.629768e-09

2 1.443262e-11

$`MR-PRESSO results`

$`MR-PRESSO results`$`Global Test`

$`MR-PRESSO results`$`Global Test`$RSSobs

[1] 495.0906

$`MR-PRESSO results`$`Global Test`$Pvalue

[1] "<0.001"

$`MR-PRESSO results`$`Distortion Test`

$`MR-PRESSO results`$`Distortion Test`$`Outliers Indices`

[1] 11 165 174 175

$`MR-PRESSO results`$`Distortion Test`$`Distortion Coefficient`

beta.exposure

[1]-5.721159

$`MR-PRESSO results`$`Distortion Test`$Pvalue

[1] 0.696

**Obesity to Estimated glomerular filtration rate (cystatin c)**

$`Main MR results`

Exposure MR Analysis Causal Estimate Sd T-stat

1 beta.exposure Raw -0.007509046 0.0011090863 -6.770479

2 beta.exposure Outlier-corrected -0.006250065 0.0009649016 -6.477412

P-value

1 2.625036e-09

2 1.289562e-08

$`MR-PRESSO results`

$`MR-PRESSO results`$`Global Test`

$`MR-PRESSO results`$`Global Test`$RSSobs

[1] 323.2091

$`MR-PRESSO results`$`Global Test`$Pvalue

[1] "<0.001"

$`MR-PRESSO results`$`Distortion Test`

$`MR-PRESSO results`$`Distortion Test`$`Outliers Indices`

[1] 10 24 33 55 65 67 73

$`MR-PRESSO results`$`Distortion Test`$`Distortion Coefficient`

beta.exposure

[1] -20.14347

$`MR-PRESSO results`$`Distortion Test`$Pvalue

[1] 0.057

**Obesity to Blood urea nitrogen levels**

$`Main MR results`

Exposure MR Analysis Causal Estimate Sd T-stat

1 beta.exposure Raw 0.02720065 0.007668099 3.547248

2 beta.exposure Outlier-corrected 0.01354891 0.005773947 2.346560

P-value

1 0.0006944021

2 0.0220064271

$`MR-PRESSO results`

$`MR-PRESSO results`$`Global Test`

$`MR-PRESSO results`$`Global Test`$RSSobs

[1] 260.974

$`MR-PRESSO results`$`Global Test`$Pvalue

1. "<0.001"

$`MR-PRESSO results`$`Distortion Test`

$`MR-PRESSO results`$`Distortion Test`$`Outliers Indices`

[1] 3 15 30 33 54 66

$`MR-PRESSO results`$`Distortion Test`$`Distortion Coefficient`

beta.exposure

100.7589

$`MR-PRESSO results`$`Distortion Test`$Pvalue

[1] 0.002

**Obesity to Cystatin C levels**

$`Main MR results`

Exposure MR Analysis Causal Estimate Sd T-stat

1 beta.exposure Raw 0.04071349 0.006609878 6.159492

2 beta.exposure Outlier-corrected 0.03542345 0.004705932 7.527405

P-value

1 3.203927e-08

2 2.395301e-10

$`MR-PRESSO results`

$`MR-PRESSO results`$`Global Test`

$`MR-PRESSO results`$`Global Test`$RSSobs

[1] 565.4579

$`MR-PRESSO results`$`Global Test`$Pvalue

1. "<0.001"

$`MR-PRESSO results`$`Distortion Test`

$`MR-PRESSO results`$`Distortion Test`$`Outliers Indices`

[1] 2 3 9 10 15 25 34 35 38 57 67 69 75

$`MR-PRESSO results`$`Distortion Test`$`Distortion Coefficient`

beta.exposure

14.93371

$`MR-PRESSO results`$`Distortion Test`$Pvalue

[1] 0.089

**Hypertension to Estimated glomerular filtration rate (cystatin c)**

$`Main MR results`

Exposure MR Analysis Causal Estimate Sd T-stat

1 beta.exposure Raw -0.001826609 0.0010402410 -1.755948

2 beta.exposure Outlier-corrected -0.002010043 0.0007427957 -2.706051

P-value

1 0.08007230

2 0.00721358

$`MR-PRESSO results`

$`MR-PRESSO results`$`Global Test`

$`MR-PRESSO results`$`Global Test`$RSSobs

[1] 1973.974

$`MR-PRESSO results`$`Global Test`$Pvalue

[1] "<0.001"

$`MR-PRESSO results`$`Distortion Test`

$`MR-PRESSO results`$`Distortion Test`$`Outliers Indices`

[1] 4 39 45 62 68 69 76 90 157 159 173 181 190 195 199 209 247 259 262

[20] 264 285 299 308 312 313

$`MR-PRESSO results`$`Distortion Test`$`Distortion Coefficient`

beta.exposure

[1] 9.125886

$`MR-PRESSO results`$`Distortion Test`$Pvalue

[1] 0.669

**Hypertension to Cystatin C levels**

$`Main MR results`

Exposure MR Analysis Causal Estimate Sd T-stat

1 beta.exposure Raw 0.008091696 0.005300277 1.526655

2 beta.exposure Outlier-corrected 0.008786451 0.003589239 2.447999

P-value

1 0.12771994

2 0.01489204

$`MR-PRESSO results`

$`MR-PRESSO results`$`Global Test`

$`MR-PRESSO results`$`Global Test`$RSSobs

[1] 3039.653

$`MR-PRESSO results`$`Global Test`$Pvalue

[1] "<0.001"

$`MR-PRESSO results`$`Distortion Test`

$`MR-PRESSO results`$`Distortion Test`$`Outliers Indices`

[1] 5 26 42 49 70 76 77 84 98 121 168 183 187 188 190 196 197 205 208

[20] 214 224 231 235 247 259 268 275 300 305 307 329 343 346 356 360 361

$`MR-PRESSO results`$`Distortion Test`$`Distortion Coefficient`

beta.exposure

-7.907116

$`MR-PRESSO results`$`Distortion Test`$Pvalue

[1] 0.734

**Type 2 diabetes to Estimated glomerular filtration rate (cystatin c)**

$`Main MR results`

Exposure MR Analysis Causal Estimate Sd T-stat

1 beta.exposure Raw -0.0022174726 0.0009174315 -2.417044

2 beta.exposure Outlier-corrected -0.0008767935 0.0006356832 -1.379293

P-value

1 0.01629331

2 0.16900341

$`MR-PRESSO results`

$`MR-PRESSO results`$`Global Test`

$`MR-PRESSO results`$`Global Test`$RSSobs

[1] 1092.846

$`MR-PRESSO results`$`Global Test`$Pvalue

1. "<0.001"

$`MR-PRESSO results`$`Distortion Test`

$`MR-PRESSO results`$`Distortion Test`$`Outliers Indices`

[1] 13 22 24 29 30 32 55 66 108 120 125 126 135 150 153 159 172 177 259

[20] 278

$`MR-PRESSO results`$`Distortion Test`$`Distortion Coefficient`

beta.exposure

[1] -152.9071

$`MR-PRESSO results`$`Distortion Test`$Pvalue

[1] 0.016

**Type 2 diabetes to Estimated glomerular filtration rate (cystatin c)**

$`Main MR results`

Exposure MR Analysis Causal Estimate Sd T-stat P-value

1 beta.exposure Raw 0.01579659 0.006465467 2.443225 0.01540289

2 beta.exposure Outlier-corrected 0.01145106 0.005352609 2.139341 0.03363235

$`MR-PRESSO results`

$`MR-PRESSO results`$`Global Test`

$`MR-PRESSO results`$`Global Test`$RSSobs

[1] 508.5755

$`MR-PRESSO results`$`Global Test`$Pvalue

[1] "<0.001"

$`MR-PRESSO results`$`Distortion Test`

$`MR-PRESSO results`$`Distortion Test`$`Outliers Indices`

[1] 89 100 125 130 186 202 206

$`MR-PRESSO results`$`Distortion Test`$`Distortion Coefficient`

beta.exposure

[1] 37.94874

$`MR-PRESSO results`$`Distortion Test`$Pvalue

[1] 0.217
